# Development of a High-Throughput Pipeline to Characterize Microglia Morphological States at a Single-Cell Resolution

**DOI:** 10.1523/ENEURO.0014-24.2024

**Published:** 2024-07-26

**Authors:** Jennifer Kim, Paul Pavlidis, Annie Vogel Ciernia

**Affiliations:** ^1^Graduate Program in Neuroscience, University of British Columbia, Vancouver, British Columbia V6T 1Z4, Canada; ^2^Djavad Mowafaghian Centre for Brain Health, Vancouver, British Columbia V6T 1Z3, Canada; ^3^Department of Psychiatry, University of British Columbia, Vancouver, British Columbia V6T 1Z4, Canada; ^4^Michael Smith Laboratories, Vancouver, British Columbia V6T 1Z4, Canada; ^5^Department of Biochemistry and Molecular Biology, University of British Columbia, Vancouver, British Columbia V6T 1Z3, Canada

**Keywords:** ImageJ, microglia, morphology, open science, R, tool development

## Abstract

As rapid responders to their environments, microglia engage in functions that are mirrored by their cellular morphology. Microglia are classically thought to exhibit a ramified morphology under homeostatic conditions which switches to an ameboid form during inflammatory conditions. However, microglia display a wide spectrum of morphologies outside of this dichotomy, including rod-like, ramified, ameboid, and hypertrophic states, which have been observed across brain regions, neurodevelopmental timepoints, and various pathological contexts. We applied dimensionality reduction and clustering to consider contributions of multiple morphology measures together to define a spectrum of microglial morphological states in a mouse dataset that we used to demonstrate the utility of our toolset. Using ImageJ, we first developed a semiautomated approach to characterize 27 morphology features from hundreds to thousands of individual microglial cells in a brain region-specific manner. Within this pool of features, we defined distinct sets of highly correlated features that describe different aspects of morphology, including branch length, branching complexity, territory span, and circularity. When considered together, these sets of features drove different morphological clusters. Our tools captured morphological states similarly and robustly when applied to independent datasets and using different immunofluorescent markers for microglia. We have compiled our morphology analysis pipeline into an accessible, easy-to-use, and fully open-source ImageJ macro and R package that the neuroscience community can expand upon and directly apply to their own analyses. Outcomes from this work will supply the field with new tools to systematically evaluate the heterogeneity of microglia morphological states across various experimental models and research questions.

## Graphical abstract

**Figure:**
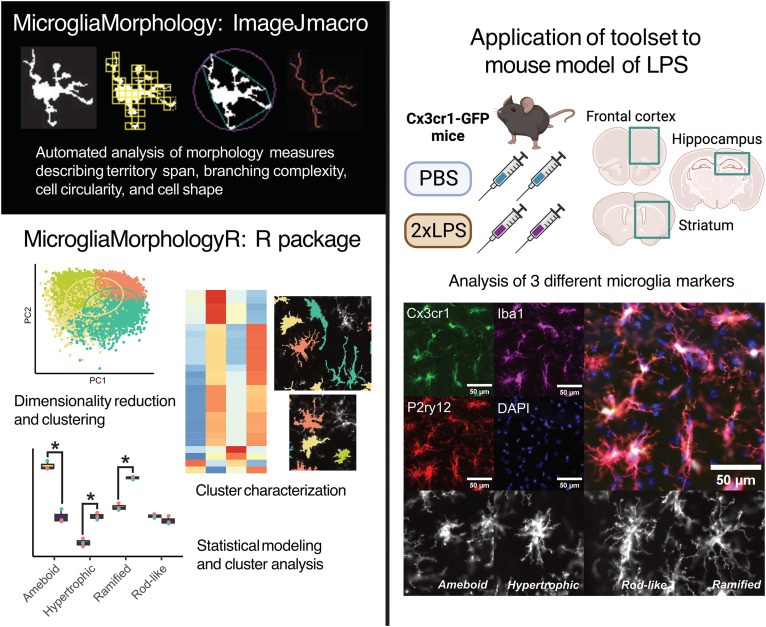
Visual Abstract

## Significance Statement

We developed an accessible, user-friendly, and open-source computational toolset for microglia morphology segmentation and analysis. While there has been considerable progress in the field to develop automated microglia morphology segmentation tools, the majority of published tools are not openly available or well documented, and there has been less transparency about the methods used to analyze the resulting morphological measures. Using our toolset, we took a data-informed approach to characterize different classes of microglia morphologies and to statistically model how membership across these forms dynamically changes across brain regions in an experimental mouse model. Application of our toolset will yield novel insights into microglia morphology differences at a single-cell resolution and in a spatially resolved manner across many different research questions.

## Introduction

Clinical and postmortem studies support a role for altered microglial function as a critical component of multiple brain disorders, including schizophrenia, autism spectrum disorder, and Alzheimer's disease (Tetreault et al., 2012; Suzuki et al., 2013; Hansen et al., 2018; Zhuo et al., 2023). In addition to their resident immune functions, microglia play critical roles to establish and maintain normal brain function including the regulation of neuronal cell number (Cunningham et al., 2013), shaping of brain circuitry (Schwarz et al., 2012; Jobling et al., 2018; Anderson et al., 2019; Nguyen et al., 2020; Mehl et al., 2022), and fine-tuning of neuronal connections (Schafer et al., 2012; Zhan et al., 2014; Wang et al., 2020), processes that have all been shown to be fundamentally disrupted in many brain disorders (Salter and Stevens, 2017; Bachiller et al., 2018; Dubbelaar et al., 2018; Hammond et al., 2018; Lenz and Nelson, 2018; Zhu et al., 2023). Microglia directly communicate with other cell types and modulate their function by releasing and responding to various molecular substrates in the brain environment including cytokines, chemokines, and neurotransmitters (Mead et al., 2012; Liu et al., 2016; Vainchtein et al., 2018; Ferro et al., 2021; Logiacco et al., 2021; Salvador et al., 2021). Thus, dysregulated microglial responses can disrupt the homeostatic brain environment and normal communication across cell types, ultimately altering brain function and behavior.

As rapid responders to their local environments, microglia exhibit a dynamic range of phenotypes defined by multiple parameters including transcriptomic signatures (Hammond et al., 2019; Li et al., 2019), epigenomic regulation (Ayata et al., 2018; Ciernia et al., 2018; Meleady et al., 2023), and changes in cellular morphology. Microglia are classically thought to engage in immune functions that are mirrored in morphology, where homeostatic microglia exhibit a ramified morphology that switches to an ameboid, unramified form in response to inflammatory signals in their environment. This morphological switch is thought to allow for increased mobility to sites of infection or injury, efficient phagocytosis, and release of cytokines into the microenvironment, all functions that are characteristic of a “pro-inflammatory” state. However, a mechanistic link between reduced branching and increased inflammatory function has only been shown recently, where increased P2RY12 potentiation of the THIK-1 channel caused decreased microglial ramifications and increased activity of the Il1b inflammasome in response to tissue damage (Madry et al., 2018). Cdk1-mediated microtubule remodeling has also recently been shown to be required for efficient cytokine trafficking and release and transformation of microglia from ramified to ameboid forms after LPS exposure in vitro and in situ (Adrian et al., 2023). In contrast, findings from other studies display a reverse relationship, where ramified microglia have been shown to phagocytose synapses during adult neurogenesis (Sierra et al., 2010; Kamei and Okabe, 2023), and ameboid microglia show reduced phagocytic capabilities in epilepsy (Abiega et al., 2016; Paolicelli et al., 2022). The relationship between form and function is clearly not as dichotomous as historically thought, and there has been an increasing effort in the field to move away from dualistic classification of microglial function and toward a clearer understanding and appreciation of the heterogeneous states of microglia (Dubbelaar et al., 2018; Paolicelli et al., 2022).

Microglia morphology has been shown to be highly context and signal dependent, displaying various forms ranging from ameboid-like under inflammatory states to hyper-ramified in mouse models of stress-induced depression, accelerated aging, and Alzheimer's disease (Beynon and Walker, 2012; Raj et al., 2014; Hellwig et al., 2016; Madry et al., 2018). Subsets of microglial populations have also been shown to display rod-like morphologies characterized by long, thin processes protruding from oval-shaped somas that retract neuron-adjacent planar processes in response to diffuse brain injury (Taylor et al., 2014). Furthermore, microglia are highly motile and never truly quiescent even in healthy conditions, constantly extending out protrusions to scan their environments for pathogens and other harmful signals, as revealed by in vivo two-photon imaging studies of the mouse brain (Davalos et al., 2005; Nimmerjahn et al., 2005; Bernier et al., 2020). Different morphological forms have been observed to be conserved across species (Geirsdottir et al., 2019) and spatially distributed across brain regions (Lawson et al., 1990; Wierzba-Bobrowicz et al., 1998; Mittelbronn et al., 2001; Tan et al., 2020; Brandi et al., 2022), neurodevelopmental timepoints (Paolicelli et al., 2011; Parakalan et al., 2012; Cengiz et al., 2019), and various pathological contexts (Davies et al., 2017; Savage et al., 2019; Meng et al., 2024). Microglial morphological forms including ramified, rod-like, hypertrophic, and ameboid have been commonly observed in both humans and mice, and microglia have been shown to display similar dendritic morphology across species (Geirsdottir et al., 2019; Savage et al., 2019; Paolicelli et al., 2022).

Microglia morphology can be explained using various measures of branch length, branching complexity, territory span, and cell circularity, which together comprise distinct sets of morphology measures that are changing in conjunction with each other. Nevertheless, studies often selectively report changes in individual features such as the number of branches or cell area alone, ultimately depicting an incomplete or biased representation of changes in microglia morphology that fail to capture true morphological states. Therefore, an analysis approach that considers contributions of all feature measures together to explain various axes of morphology is necessary to gain a better understanding of a microglia's actual morphological state and relationship to cellular function. A plethora of tools exist to analyze microglia morphology (Fernández-Arjona et al., 2017; York et al., 2018; Young and Morrison, 2018; Salamanca et al., 2019; Clarke et al., 2021) but often involve significant effort to extract meaningful information at a larger scale, as many approaches that allow for the analysis of morphological information at a cellular resolution involve manually choosing and segmenting individual cells within an image. This introduces a potential bias for which cells are selected and vastly limits the feasible sample size for analysis. While there has been considerable progress in the development of toolsets which automate these time-consuming steps (Clarke et al., 2021; Melo et al., 2023), there has been less development, transparency about, and availability of methods to analyze the resulting morphological measures (Reddaway et al., 2023). The underlying code and datasets for the majority of published toolsets are not openly available nor well documented, further limiting the uptake and progression of the most up-to-date toolsets by the larger research community (Reddaway et al., 2023).

Here, we describe an accessible and open-source morphology analysis toolset: MicrogliaMorphology (ImageJ tool) and MicrogliaMorphologyR (R package), which supply the field with new tools to systematically evaluate the heterogeneity of microglia morphological states by considering 27 different measures of microglia morphology. To demonstrate use cases for our toolset, we characterized and analyzed microglia morphology in an experimental model of repeated immune stimulation by peripheral lipopolysaccharide (LPS) administration, a commonly used model (Wendeln et al., 2018; H. Jung et al., 2023) which induced population shifts in the four major classes of microglia morphology in our dataset: ramified, hypertrophic, ameboid, and rod-like. Application of MicrogliaMorphology and MicrogliaMorphologyR by the scientific community will yield novel insights into microglia morphology differences in the brain at a single-cell resolution and in a spatially resolved manner across various experimental models and research questions. Additionally, the toolset is not limited to microglia morphology alone but can also be applied in the same way to characterize morphology of other cell types.

## Materials and Methods

### In vivo experiments

All experiments were conducted in accordance with the University of British Columbia guidelines, with approval from the Canadian Council on Animal Care Committee. Mice were housed in groups of two to four on a regular 12 h light/dark cycle, and all experiments were performed in regular light during the mouse's regular light cycle. CX3C motif chemokine receptor 1 (Cx3cr1) is a commonly used microglial marker that is expressed on microglia and other immune cells, and cells under control of the endogenous Cx3cr1 locus express GFP (IMSR_JAX:005582; S. Jung et al., 2000). Eight-week-old male and female Cx3cr1-GFP mice bred on a C57BL/6J background (*n* = 3/condition, 2 females and 1 male) were intraperitoneally injected with 0.5 mg/kg lipopolysaccharide (LPS; lipopolysaccharides from *E. coli* O55:B5, Sigma-Aldrich L5418) or vehicle solution (PBS; phosphate-buffered saline, Fisher BioReagents BP3991) once every 24 h for 2 d.

#### 1xLPS experiments (Extended Data Fig. 3-1)

Mice were housed in groups of two to four on a reversed 12 h light/dark cycle. Because mice are nocturnal animals, all experiments were performed in red light during the mouse's dark cycle when they are most active. Eight-week-old male and female C57BL/6J mice (*n* = 2/sex/condition) were intraperitoneally injected once with 1.0 mg/kg lipopolysaccharide (LPS; lipopolysaccharides from *E. coli* O55:B5, Sigma-Aldrich L5418) or vehicle solution 1xPBS (phosphate-buffered saline, Fisher BioReagents BP3991).

### Tissue collection

Three hours (2xLPS experiments) or 24 h (1xLPS experiments) after the final injection, mice were quickly anesthetized with isoflurane and transcardially perfused with 15 ml of 1xPBS before brains were extracted for downstream immunohistochemistry experiments. Extracted brains were immersion fixed in 4% paraformaldehyde for 48 h before cryoprotecting in 30% sucrose for 48 h prior to cryosectioning. Cryoprotected brains were then sectioned at 30 µm on the cryostat, collected in 1xPBS for long-term storage, and processed for immunohistochemistry.

### Immunohistochemistry

**Figure 1.**
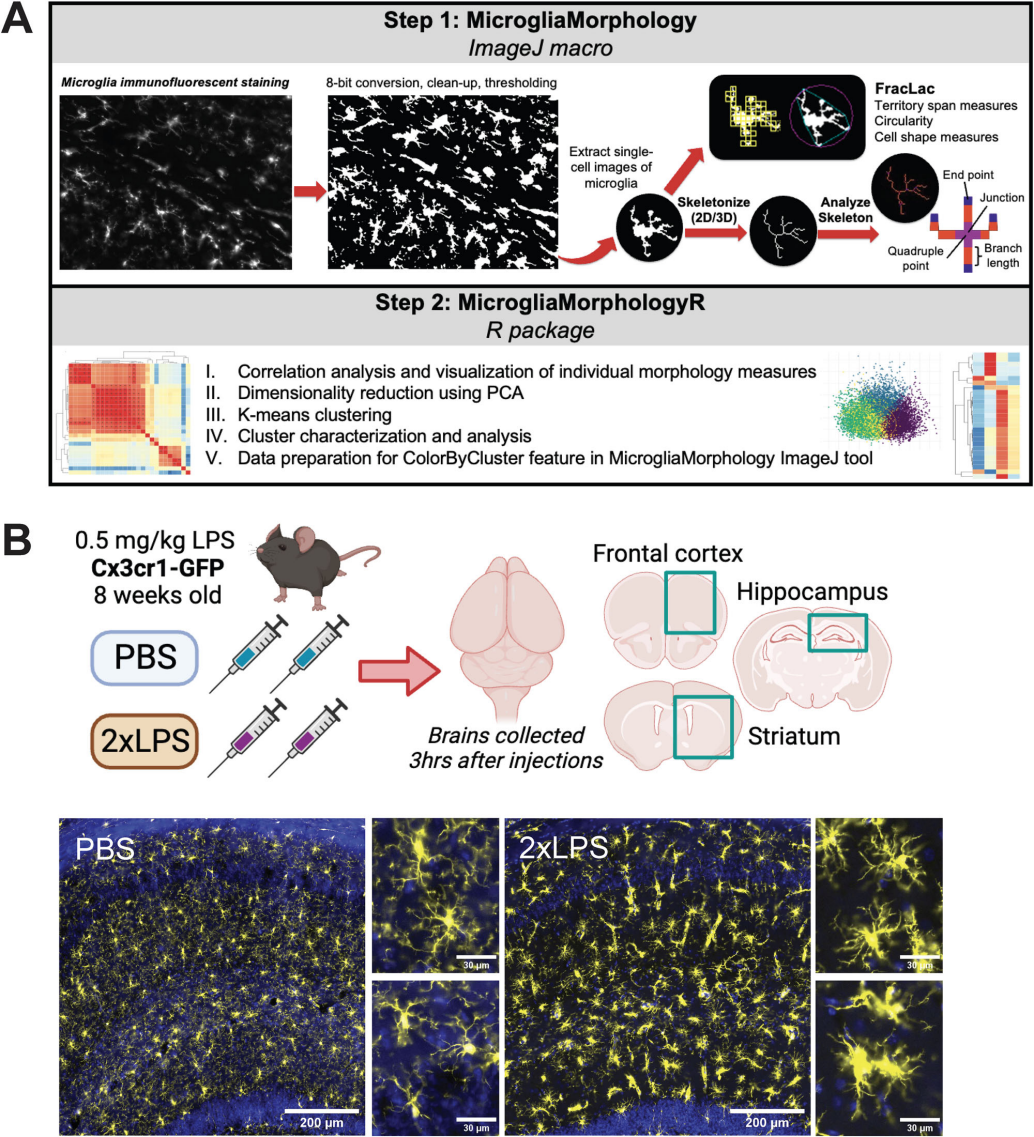
Study overview. ***A***, Outline of steps involved in MicrogliaMorphology and MicrogliaMorphologyR. ***B***, Experimental mouse model used for dataset described throughout paper, made using Biorender. Example images from the dorsal hippocampus with individual microglia insets for each treatment condition. Microglia (Iba1) in yellow and DAPI nuclear stain in blue. Full size images scale bar 200 µm, insets 30 µm.

Thirty-micrometer brain sections were immunofluorescently stained for various markers of microglia: ionized calcium binding adaptor molecule 1 (Iba1) and/or purinergic receptor P2Y12 (P2ry12) to analyze microglial morphology. Brain sections in the 1xLPS experiments (Extended Data Fig. 3-1) were stained with only Iba1, and brain sections in the 2xLPS experiments (Fig. 1*B*) were stained with both Iba1 and P2ry12. Free-floating brain sections were washed three times for 5 min each in 1xPBS, permeabilized in 1xPBS + 0.5% Triton (Fisher BioReagents BP151-500) for 5 min, and incubated in blocking solution made of 1xPBS + 0.03% Triton + 1% bovine serum albumin (BSA; Bio-techne Tocris 5217) for 1 h. After the blocking steps, sections were incubated overnight at 4°C in primary antibody solution containing 2% normal donkey serum (NDS; Jackson ImmunoResearch Laboratories 017-000-121) + 1xPBS + 0.03% Triton + primary antibodies (guinea pig anti-Iba1: 1:1,000, Synaptic Systems 234 308; rabbit anti-P2RY12: 1:500, AnaSpec AS-55043A). After primary antibody incubation, sections were washed three times for 5 min each with 1xPBS + 0.03% Triton before incubating for 2 h in secondary solution containing 2% NDS + DAPI (1:1,000; BioLegend 422801) + secondary antibodies (Alexa Fluor 647 donkey anti-guinea pig: 1:500, Jackson ImmunoResearch Laboratories 706-605-148; Alexa Fluor 568 donkey anti-rabbit: 1:500, Invitrogen A10042). Sections were washed three times for 5 min each with 1xPBS + 0.03% Triton before being transferred into 1xPBS for temporary storage before mounting. Sections were mounted onto microscope slides (Premium Superfrost Plus Microscope Slides, VWR CA48311-703) and air-dried before being coverslipped with mounting media (ProLong Glass Antifade Mountant, Invitrogen P36980; 24 × 60 mm 1.5H High Performance Coverslips, Marienfeld 0107242).

### Imaging

All mounted brain sections were imaged on the ZEISS Axioscan 7 microscope slide scanner at 20× magnification with a step size of 0.5 µm using the *z*-stack acquisition parameters within the imaging software (ZEISS ZEN 3.7). During image acquisition, Extended Depth of Focus (EDF) images were created using maximum projection settings and saved as the outputs in the final .czi files. Maximum projection EDF images only compile the pixels of highest intensity at any given position in a *z*-stack to construct a new 2D image which retains the 3D information. Using ImageJ, we created .tiff images of each fluorescent channel from .czi files and selected and saved .tiffs of brain regions of interest (ROIs) to use as input for downstream morphological analysis in MicrogliaMorphology and MicrogliaMorphologyR. We focused our analyses on multiple brain regions including the hippocampus, frontal cortex, and striatum, as well as subregions within them (Extended Data Fig. 4-1*D*). Images of coronal brain sections containing these regions were aligned to the Allen Brain Atlas (mouse.brain-map.org; Pinskiy et al., 2015) using the ImageJ macro FASTMAP (Terstege et al., 2022).

### MicrogliaMorphology

MicrogliaMorphology is designed to be a user-friendly ImageJ macro that wraps around existing ImageJ plugins AnalyzeParticles, Skeletonize (2D/3D), and AnalyzeSkeleton and is written using the ImageJ macro (IJM) language (Schneider et al., 2012). All supporting code for MicrogliaMorphology is available on GitHub at https://github.com/ciernialab/MicrogliaMorphology, and a detailed video tutorial which includes relevant troubleshooting steps is available on YouTube at https://www.youtube.com/watch?v=YhLCdlFLzk8. After MicrogliaMorphology is installed into the user's ImageJ plugins folder as described in the GitHub repository, it will appear in the Plugins dropdown menu from the ImageJ toolbar, where it can be clicked on to begin the user prompts. In Step 1, users are prompted to measure dataset-specific parameters, which are critical because every imaging dataset is prepared and acquired differently and thus requires user input to determine what parameters most appropriately and accurately capture microglia morphology within individual datasets. Image thresholding parameters including the method and radius considered for auto local thresholding are important to determine the most appropriate thresholding method which captures full, single microglial cells without losing branching connectivity. Area ranges that accurately describe single microglial cells are important to exclude any artifacts of 2D representation in the EDFs such as cell particles and incomplete cells or multiple cells that are overlapping. Dataset-specific image thresholding and cell area parameters determined by these initial steps are then called to within the macro to inform downstream steps of MicrogliaMorphology. In the subsequent steps, the only user input involves following prompts to select input folders to call from and output folders to write to, with the option of batch processing. All protocols, computation, and analysis described below have been written to be automated within MicrogliaMorphology, unless otherwise specified.

Step 2 after determining dataset-specific parameters is thresholding input .tiff fluorescent images. MicrogliaMorphology cleans up and thresholds input images according to standard protocol (Young and Morrison, 2018): images are binarized and converted to grayscale before the brightness and contrast are enhanced, unsharp mask filter applied to clarify existing detail, despeckle function applied to remove noise, and auto local or auto thresholding applied to the resulting images. Then, a second despeckle step is applied before dilation, and connection steps are applied to connect branches, after which outliers are removed. The final images are then used as input for Step 3, which uses AnalyzeParticles and ROI manager functions to create and save new images of single cells which pass the area criteria specified in Step 1. Generation of single-cell images in this step allows for the measurement of morphology measures at an unprecedented cellular resolution from hundreds to thousands of different microglia cells, each with unique identifiers.

The single-cell images are used as input for Step 4, which uses Skeletonize (2D/3D; Lee et al., 1994) and AnalyzeSkeleton (Arganda-Carreras et al., 2010) to generate measures of different morphology features including maximum branch length, average branch length, and numbers of end point voxels, junction voxels, triple points, branches, junctions, slab voxels, and quadruple points for every cell. MicrogliaMorphology saves these outputs as individual .csv files, which contain all skeleton measures for every individual cell in the dataset, marked by unique identifiers. Step 5 involves FracLac (Karperien, 1999, 2013), a plugin separate from MicrogliaMorphology which uses fractal analysis to measure additional morphology features including the width of bounding rectangle, maximum radius from hull's center of mass, maximum span across hull, diameter of bounding circle, maximum radius from circle's center of mass, perimeter, mean radius, mean radius from circle's center of mass, area, foreground pixels, height of bounding rectangle, max/min radii from circle's center of mass, relative variation (CV) in radii from circle's center of mass, span ratio of hull (major/minor axis), max/min radii from hull's center of mass, relative variation (CV) in radii from hull's center of mass, density of foreground pixels in hull area, and circularity. Because FracLac is incompatible with the IJM language, it was not integrated into our MicrogliaMorphology macro, and Step 5 must be completed using FracLac-specific user prompts and common parameters outlined in Young and Morrison (2018). Steps to batch process the single-cell images generated from MicrogliaMorphology using FracLac are written out in detail on our GitHub page for MicrogliaMorphology. Importantly, the unique identifiers for each cell are retained in the FracLac output, allowing for integration with the MicrogliaMorphology measures. The final AnalyzeSkeleton output from MicrogliaMorphology (Step 4) and FracLac (Step 5) are merged using a custom function in MicrogliaMorphologyR to generate a final, master .csv file containing measures for 27 different morphology features for every individual cell.

An additional feature within MicrogliaMorphology is the ColorByCluster feature, which allows the user to color the microglia cells in the original .tiff input images by their *k*-means cluster identification (see below, MicrogliaMorphologyR). This is a unique feature of MicrogliaMorphology that allows the user to visually validate their morphological clusters and gain insight into their spatial distribution in the brain (Fig. 3*E*). All original input immunofluorescent .tiff images containing FASTMAP subregion ROIs, thresholded images generated by MicrogliaMorphology, thresholded single-cell images generated by MicrogliaMorphology, and all analysis code for the data presented in this paper are available on the Open Science Framework (OSF) at https://osf.io/8t5c2/. Finally, tidied up datasets that were used for analysis in this paper are included as part of MicrogliaMorphologyR and can be loaded with the package (data_1xLPS_mouse, data_2xLPS_mouse, data_2xLPS_mouse_fuzzykmeans, and data_ImageTypeComparison).

### MicrogliaMorphologyR

MicrogliaMorphologyR is an R package that wraps several existing packages including tidyverse, Hmisc, pheatmap, factoextra, lmerTest, lme4, Matrix, SciViews, ggpubr, glmmTMB, DHARMa, rstatix, and gridExtra (Bates et al., 2015, 2023; Auguie, 2017; Brooks et al., 2017; Kuznetsova et al., 2017; Kolde, 2019; Wickham et al., 2019; Kassambara, 2020; Grosjean, 2022; Hartig, 2022; Harrell, 2023; Kassambara, 2023a,b). While our ImageJ macro, MicrogliaMorphology, facilitates the semiautomated measurement of 27 individual morphology features at a single-cell level, our complementary R package, MicrogliaMorphologyR, allows for analysis and visualization of this data to characterize microglia morphological states and gain insight into their relevance in experimental models. Functions within MicrogliaMorphologyR integrate correlation analyses and statistical modeling approaches and are used in conjunction with dimensionality reduction by principal component analysis and *k*-means clustering to characterize morphological states and quantify population shifts in the experimental model of choice. MicrogliaMorphologyR also includes exploratory data analysis functions to generate heatmap and boxplot visualizations of data in flexible ways including at the single-cell level, animal level, and experimental condition level. Furthermore, MicrogliaMorphologyR includes functions for generating quality control metrics on input data such as identifying values that dominate and disproportionately skew feature distributions, data normalization options, and performing linear mixed effects modeling, ANOVA, and other statistical analyses on the input dataset. All source code for MicrogliaMorphologyR and descriptions of functions can be found on GitHub at https://github.com/ciernialab/MicrogliaMorphologyR.

### Statistical analysis of microglia markers

To test for LPS-induced morphological population shifts in cluster membership, we first calculated the percentage of cells in each morphology cluster for every brain region and antibody for every mouse using the “clusterpercentage” function within MicrogliaMorphologyR. To assess how cluster membership changes with LPS treatment across microglia markers, we filtered for each individual brain region before fitting a generalized linear mixed model using a beta distribution to model the percentage of cluster membership as a factor of cluster identity, treatment, and antibody interactions with MouseID as a repeated measure [“percentage ∼ Cluster*Treatment*Antibody + (1|MouseID)”] using the “stats_cluster.animal” function from MicrogliaMorphologyR, which is wrapped around the glmmTMB R package (Brooks et al., 2017). The beta distribution is suitable for values like percentages or probabilities that are constrained to a range of 0–1. We fit separate models for each brain region individually because the model would not converge when considering Cluster*Treatment*Antibody*BrainRegion interactions together, and we were more interested in Cluster*Treatment*Antibody interactions for our analysis. Tests between treatments (PBS vs 2xLPS) were corrected for multiple comparisons across clusters and antibodies (∼Treatment|Cluster|Antibody) using the Bonferroni’s method and *q* values <0.05 were considered statistically significant (Extended Data Table 4-2).

To highlight the utility of MicrogliaMorphologyR for assessing changes in individual morphology measures, we used the “stats_morphologymeasures.animal” function, which is wrapped around the “lmer” function from the lme4 package in R, to fit linear mixed effects models for each brain region to the measure values as a factor of treatment and antibody interactions with MouseID as a repeated measure [“Value ∼ Treatment*Antibody + (1|MouseID)”]. We focused on cell area as an example. Tests between treatments (PBS vs 2xLPS) were corrected for multiple comparisons across antibodies (∼Treatment|Antibody) using Bonferroni’s method and *q* values <0.05 were considered statistically significant (Extended Data Table 4-4).

### Code accessibility

The datasets generated and/or analyzed during the current study are freely available to download, and all relevant materials including input images, data, and supporting code used in this study are available on the OSF website at https://osf.io/8t5c2/. The ImageJ and R code underlying both MicrogliaMorphology and MicrogliaMorphologyR, as well as the R code for the analysis demonstrated in this paper are all available through GitHub repositories at https://github.com/ciernialab/MicrogliaMorphology and https://github.com/ciernialab/MicrogliaMorphologyR.

## Results

### Morphology analysis toolset: ImageJ macro MicrogliaMorphology

Using ImageJ plugins (Karperien, 1999, 2013; Young and Morrison, 2018), we have developed an accessible and user-friendly ImageJ tool, MicrogliaMorphology, that automates the characterization of a vast range of morphology features from hundreds to thousands of individual microglial cells (Fig. 1*A*). We compiled the ImageJ code into an ImageJ macro format such that users can simply click through options for their morphology analysis and specify where to read and write files. The following steps are automated within the MicrogliaMorphology ImageJ macro such that users can easily perform morphology analysis by following user prompts within ImageJ. Briefly, immunofluorescence images are binarized, cleaned up to remove any background noise, and thresholded according to standard protocol (Young and Morrison, 2018) before individual cell images are created based on area measurements to exclude any artifacts that arise as a product of 2D image representation. The newly generated single-cell images are then used downstream as input for ImageJ plugins AnalyzeSkeleton (Arganda-Carreras et al., 2010) and FracLac (Karperien, 1999, 2013; Young and Morrison, 2018; Fig. 1*A*) to measure 27 unique morphology features from individual cells in a high-throughput and semiautomated manner (Fig. 1*A*). Importantly, MicrogliaMorphology saves the ROI information of each individual cell in the output file so that the user has the option of linking the individual cells back to their spatial locations in the original input images. We have enabled this option through the complementary ColorByCluster functions in MicrogliaMorphology and MicrogliaMorphologyR.

**Figure 2.**
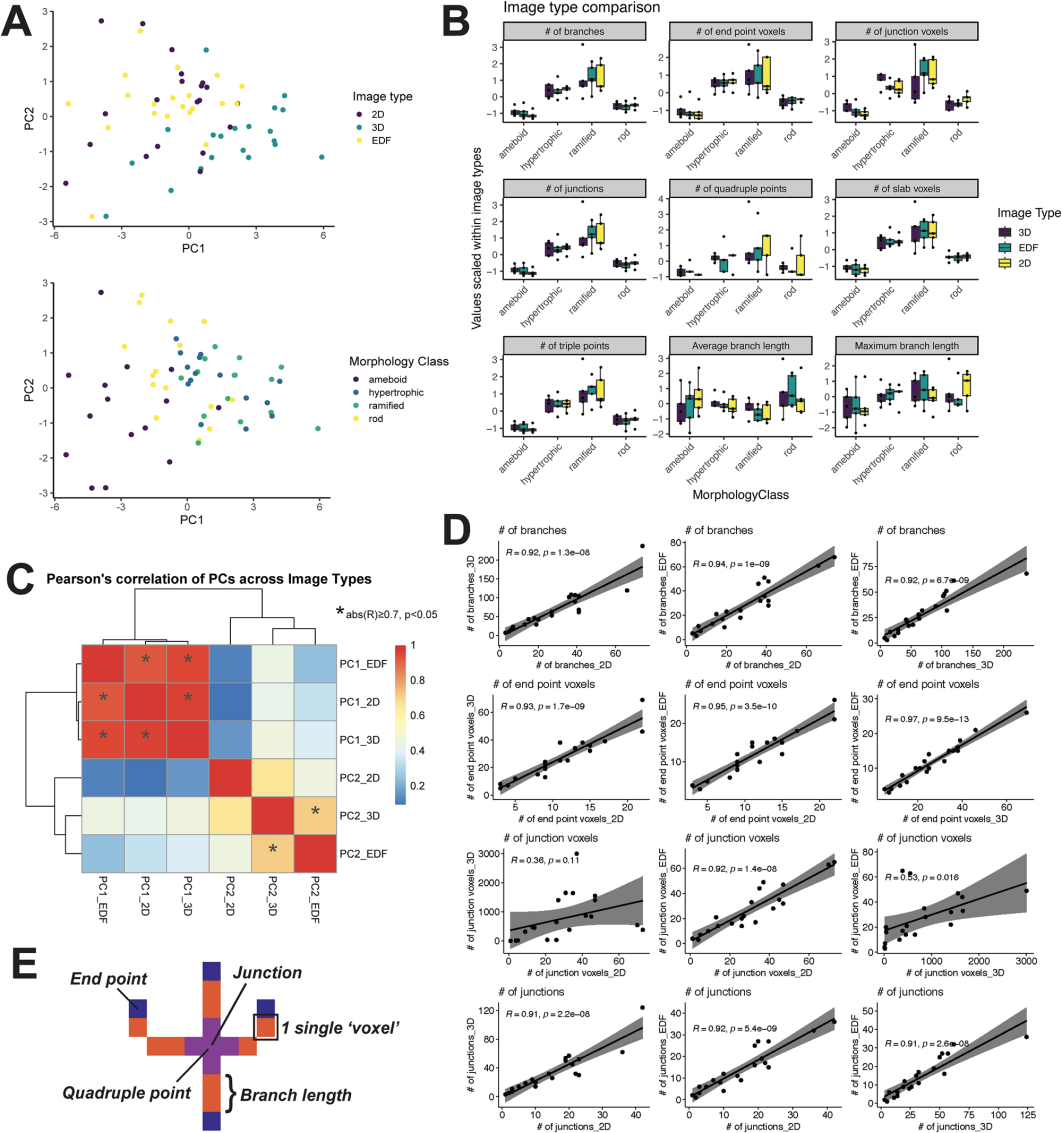
Comparison of 3D versus EDF versus single-plane 2D image types. ***A***, Samples represented in principal components space and colored by image type or morphology class. Each point is either a 2D, 3D, or EDF representation of 1 of 20 different cells. ***B***, Comparison of changes across morphological classes when cells are represented in 2D, 3D, or EDF forms. Values on plots are *z*-scores (centered and scaled) calculated within image type. ***C***, Spearman's correlation of PCs 1–2 after dimensionality reduction across image types. ***D***, Individual Pearson’s correlations between image types for specific morphology features measured using AnalyzeSkeleton. ***E***, Visual description of morphology features measured using AnalyzeSkeleton. See Extended Data Figure 2-1 and Extended Data Tables 2-1 and 2-2 for additional data.

As MicrogliaMorphology is wrapped around the ImageJ plugin FracLac (Karperien, 1999, 2013), which can only handle 2D input, it is limited to the analysis of 2D images. While 3D reconstructions of microglia offer the benefit of finer-grained detail for morphology analysis, the size of the generated datasets and the time and resources necessary to construct and process such data limits the applicability of 3D measurements to smaller areas, inevitably making the analysis comparatively low throughput. Two-dimensional image options include maximum projection EDF images, which only compile the pixels of highest intensity at any position in a *z*-stack to construct a new 2D image which preserves some 3D information, versus individual 2D images sampled that only capture single-plane information. To assess how accurately microglial morphology is represented in 3D image forms as compared with 2D image forms (EDF, single-plane 2D), we analyzed just the skeletal measures (Fig. 2*E*) from 20 individual microglial cells manually isolated from *z*-stack images of the hippocampus of female mice. AnalyzeSkeleton, one of the ImageJ plugins that MicrogliaMorphology is wrapped around, is able to measure skeletal features in both 2D and 3D, enabling direct comparisons across different image forms. Five cells of each morphological type (ameboid, ramified, hypertrophic, rod-like) were manually classified and selected out from the original 3D *z*-stacks, from which EDF images were generated and 2D single-plane images from the center of the stack were saved to create the test dataset.

10.1523/ENEURO.0014-24.2024.f2-1 Figure 2-1 E*x*tended *comparison of 3D vs. 2D image types.* (A) Changes in the raw values of AnalyzeSkeleton measures across 3D, EDF, and 2D image types. Each line is an individual cell represented in 3D, EDF, and 2D. (B) Individual Pearson’s correlations between image types for specific morphology features measured using AnalyzeSkeleton. (C) Spearman’s correlation of skeletal morphology measures to first 5 PCs after dimensionality reduction. (*abs(R) ≥ 0.8, p < 0.05). Download Figure 2-1, TIF file.

10.1523/ENEURO.0014-24.2024.t2-1 Table 2-1 Pearson’s correlation of principal components and p-values for correlations, related to Fig. 2C. Download Table 2-1, DOC file.

10.1523/ENEURO.0014-24.2024.t2-2 Table 2-2 Spearman’s correlation of morphology measures to principal components and p-values for correlations, related to Fig. 2-1C. Download Table 2-2, DOC file.

As expected, the raw values of all skeletal measures quantified decreased considerably when measured from 3D to either of the 2D image forms (Extended Data Fig. 2-1*A*). While cells with more ramification and cell branching complexity (hypertrophic, ramified classes) were better captured in 3D (Fig. 2*A*), relative differences in morphology across the four different forms were well conserved across image types (Fig. 2*B*). After dimensionality reduction, PC2, which mostly captured variability described by the maximum branch length (Extended Data Fig 2-1*C*, Extended Data Table 2-2), was highly correlated between 3D and EDF forms but not 3D and 2D forms (Fig. 2*C*, Extended Data Fig. 2-1*B*, Extended Data Table 2-1). Cell branching complexity, as described by numbers of junctions, end point voxels, branches, slab voxels, and triple points, was highly correlated across image types (Fig. 2*D*, Extended Data Fig. 2-1*B*). Although the number of junctions was also highly correlated across image types, junction voxels, or the numbers of actual pixels which make up the junctions, had low correlation scores when comparing 3D images with either of the 2D image types (Fig. 2*D*), which is unsurprising, as a 3D image would retain much more of this kind of information. Numbers of quadruple points, which would describe the most complex type of junction, was the least well captured in 2D representations (Extended Data Fig. 2-1*B*). As expected, these results together indicate that EDF images better retain 3D skeletal information than 2D forms do but that relative group differences between ameboid, hypertrophic, ramified, and rod-like morphologies are still maintained across image forms.

### Morphology analysis toolset: R package MicrogliaMorphologyR

Once the 27 morphological features are measured from individual microglia using the MicrogliaMorphology ImageJ macro and FracLac, they are concatenated into a final output data file which can be analyzed further to gain insight into microglia morphology changes in any given experimental model. We have provided an R package, MicrogliaMorphologyR, which contains a set of functions that implement one set of approaches for such an analysis in R. Using MicrogliaMorphologyR, the user can conduct exploratory data analysis to generate visualizations of their own data in flexible ways including heatmaps of how morphological features vary across morphological clusters and boxplots of how morphological populations shift at the subject-level across treatment conditions. Functions within MicrogliaMorphologyR are used in conjunction with principal component analysis and *k*-means clustering to gain further insight into microglia morphology features, classify individual cells by their morphological states, and allow for the quantification of morphological population shifts in experimental contexts. MicrogliaMorphologyR also includes functions for generating quality control metrics on input data such as identifying values that dominate and disproportionately skew feature distributions, data normalization options, visually exploring different sources of variability in the dataset, and performing ANOVA analysis, linear mixed effects modeling, and other statistical analyses on the input dataset.

### Application to 2xLPS mouse dataset

To demonstrate the utility of MicrogliaMorphology and MicrogliaMorphologyR, we describe an experimental dataset collected from the brains of 8-week-old Cx3cr1-GFP mice. Male and female mice were injected peripherally with two daily injections of vehicle or 0.5 mg/kg lipopolysaccharide (LPS), a major structural component of gram-negative bacteria that is commonly used to induce and study microglial responses in the brain (Fig. 1*B*). To capture a diverse range of microglia morphologies across multiple brain regions, we focused on profiling the frontal cortex, striatum, and hippocampus from six individual mice (*n* = 3/treatment, 2 females and 1 male). Using MicrogliaMorphology, we were able to quantify 27 morphological features from a total of 43,332 individual microglial cells, which made up our input dataset for analysis.

**Figure 3.**
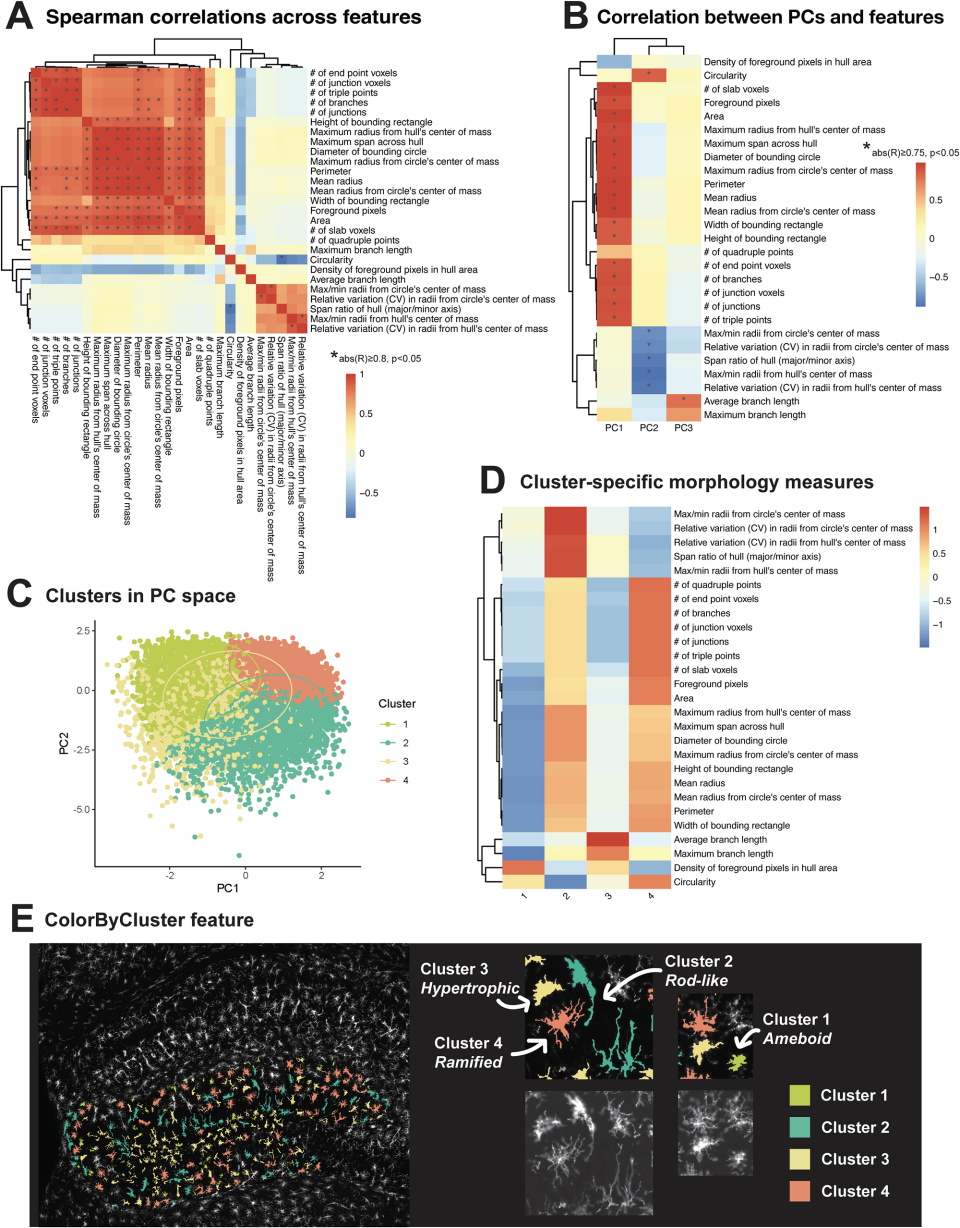
Characterization of morphological clusters in 2xLPS dataset. ***A***, Spearman's correlation matrix of 27 features measured by MicrogliaMorphology [*abs(*R*) ≥ 0.8; *p* < 0.05]. ***B***, Spearman's correlation of morphology measures to first three PCs after dimensionality reduction [*abs(*R*) ≥ 0.75; *p* < 0.05]. ***C***, Cluster classes displayed in PCs 1–2 space. ***D***, Average values for all 27 morphology features, scaled across clusters. ***E***, Individual cells spatially registered back to original images and visually annotated by morphological class using ColorByCluster feature. See Extended Data Figure 3-1 and Extended Data Tables 3-1–3-4 for additional data.

Within the pool of morphology measures, we defined distinct sets of highly correlated features that describe different aspects of morphology, including branching complexity, area and territory span, branch length, and cell shape (Fig. 3*A*, Extended Data Table 3-1). Spearman correlation analysis across all 27 features revealed that those which describe branching complexity (number of end point voxels, junction voxels, triple points, branches, junctions) were highly correlated to each other compared with the other features [abs(*R*) ≥ 0.8; *p* < 0.05; Extended Data Table 3-1]. Similar correlations were observed for features that describe area and territory span (width of bounding rectangle, maximum radius from hull's center of mass, maximum span across hull, diameter of bounding circle, maximum radius from circle's center of mass, perimeter, mean radius, mean radius from circle's center of mass, area, number of slab voxels, foreground pixels, height of bounding rectangle), branch length (maximum branch length, average branch length), and cell shape (max/min radii from bounding circle's and hull's center of mass, relative variation in radii from bounding circle's and hull's center of mass). As expected, cell circularity was highly negatively correlated with span ratio of the bounding hull (major/minor axis), a measure whose higher value indicates greater cell oblongness (Fig. 3*A*, Extended Data Table 3-1). The relationships observed among the 27 morphology features were consistently captured in another LPS dataset collected under entirely different conditions (reversed light cycle, single 1.0 mg/kg LPS exposure, 24 h collection time; Extended Data Fig. 3-1*A*, Extended Data Table 3-2), demonstrating that MicrogliaMorphology is able to consistently and robustly capture different aspects of microglia morphology across experimental models.

10.1523/ENEURO.0014-24.2024.f3-1 Figure 3-1 A*n*alysis *of 1xLPS morphology measures and clusters.* (A) Spearman’s correlation matrix of 27 features measured by MicrogliaMorphology. (*abs(R) ≥ 0.8, p < 0.05) (B) Spearman’s correlation of morphology measures to first 3 PCs after dimensionality reduction. (*abs(R) ≥ 0.75, p < 0.05) (C) Optimal k-means clustering parameters determined using within sum of squares and gap statistic techniques. Cluster classes displayed in PC space. (D) Average values for all 27 morphology features, scaled across clusters. Download Figure 3-1, TIF file.

10.1523/ENEURO.0014-24.2024.t3-1 Table 3-1 Spearman’s correlation of morphology measures and p-values for correlations, related to Fig. 3A. Download Table 3-1, DOC file.

10.1523/ENEURO.0014-24.2024.t3-2 Table 3-2 Spearman’s correlation of morphology measures and p-values for correlations, relate to Fig. 3-1A. Download Table 3-2, DOC file.

10.1523/ENEURO.0014-24.2024.t3-3 Table 3-3 Spearman’s correlation of morphology measures to principal components and p-values for correlations, related to Fig. 3-1B. Download Table 3-3, DOC file.

10.1523/ENEURO.0014-24.2024.t3-4 Table 3-4 Spearman’s correlation of morphology measures to principal components and p-values for correlations, related to Fig. 3B. Download Table 3-4, DOC file.

### Dimensionality reduction and soft clustering

To define morphological states from our 27-feature dataset, we performed dimensionality reduction using principal component analysis followed by fuzzy *k*-means clustering on the first three principal components (PCs), which together explained 84.6% of the variability in the dataset (Extended Data Fig. 4-1*B*). Spearman's correlation of the first three PCs to the 27 features showed that each PC was differentially correlated to and described by different sets of morphology features [abs(*R*) ≥ 0.75; *p* < 0.05; Fig. 3*B*, Extended Data Table 3-4]. PC1 was highly positively correlated to features describing branching complexity and territory span, meaning that individual cells with greater branching complexity or area had higher PC1 scores (Fig. 3*B*, Extended Data Table 3-4). PC1 was also highly positively correlated to density of foreground pixels in hull area, which describes a cell's occupancy within its territory and can be a proxy for soma and/or branch thickness. Taking these correlations together, PC1 captured the variability in the dataset driven by branching complexity, territory span, and territory occupancy. In a similar manner, PC2 captured variability driven by cell circularity and cell shape and PC3 captured variability driven by average branch length (Fig. 3*B*, Extended Data Table 3-4). In line with our feature analysis (Fig. 3*A*, Extended Data Fig. 3-1*A*), we also observed that the PCs were similarly described by the same distinct sets of features in the 1xLPS dataset (Extended Data Fig. 3-1*B*, Extended Data Table 3-3).

The first three PCs were used as input downstream for fuzzy *k*-means clustering (Cebeci, 2019), a soft clustering method that is similar in concept and algorithm to *k*-means clustering, which partitions data points within a given dataset into defined numbers of clusters based on their proximity to the nearest cluster's centroid. In fuzzy *k*-means, data points are not exclusively assigned to just one cluster, but rather given scores of membership to all clusters, allowing for “fuzziness” or overlap between two or more clusters. This allows for additional characterization of high-scoring cells within each cluster, cells with more ambiguous identities, and other cases that the user might be interested in, which might be informative to their specific dataset. Fuzzy *k*-means also assigns a final “hard” cluster assignment based on the class with the highest membership score, which can be used as input for downstream analysis. These final cluster assignments were then used for the analysis of the 2xLPS mouse dataset in this paper, unless otherwise specified. Using exploratory data analysis methods including the within sum of squares and silhouette methods (Extended Data Fig. 4-1*C*), we found that a clustering parameter of four yields the highest degree of within-cluster similarity and was thus the most optimal parameter to use for our example 2xLPS dataset.

### Cluster characterization and analysis

Once cluster membership was defined using *k*-means clustering, we further explored what features describe the different clusters (Fig. 3*D*) and how cells belonging to each cluster visually look using the ColorByCluster feature in MicrogliaMorphology (Fig. 3*E*). We recommend that users always perform these steps in addition to the initial clustering optimization steps (Extended Data Fig. 4-1*C*) to verify that the clusters defined within their datasets are morphologically distinct and in line with expected differences in microglia morphology. We computed the average values for all 27 morphology measures, scaled across clusters, to characterize how each morphological cluster was differentially defined by the various morphology measures relative to the other clusters (Fig. 3*D*). Cluster 1 had the lowest branching complexity and territory span, resembling the classic ameboid shape in the original images upon visual confirmation using the ColorByCluster feature in MicrogliaMorphology. Cluster 2 had the greatest oblongness and branching inhomogeneity and resembled rod-like shapes; Cluster 3 had the highest branch lengths and density of foreground pixels in the hull with average territory span values relative to the other clusters and appeared hypertrophic; and Cluster 4 had the greatest branching complexity, territory span, and circularity and appeared ramified (Fig. 3*D*,*E*). Cluster 1 (ameboid), 2 (rod-like), and 4 (ramified) cells had relatively lower overlap in PC space with each other compared with Cluster 3 (hypertrophic) cells, which highly overlapped with Cluster 1 and Cluster 2 cells (Fig. 3*C*). This was expected, as hypertrophic cells represent a state between ameboid and rod-like forms on the morphological spectrum (Fig. 3*C*,*E*). Clusters were similarly described by the different morphology measures in the independent 1xLPS dataset (Extended Data Fig. 3-1*C*,*D*), further pointing to MicrogliaMorphology and MicrogliaMorphologyR as a robust means to characterize and analyze microglia morphologies.

### LPS-induced changes in cluster membership and individual morphology measures

**Figure 4.**
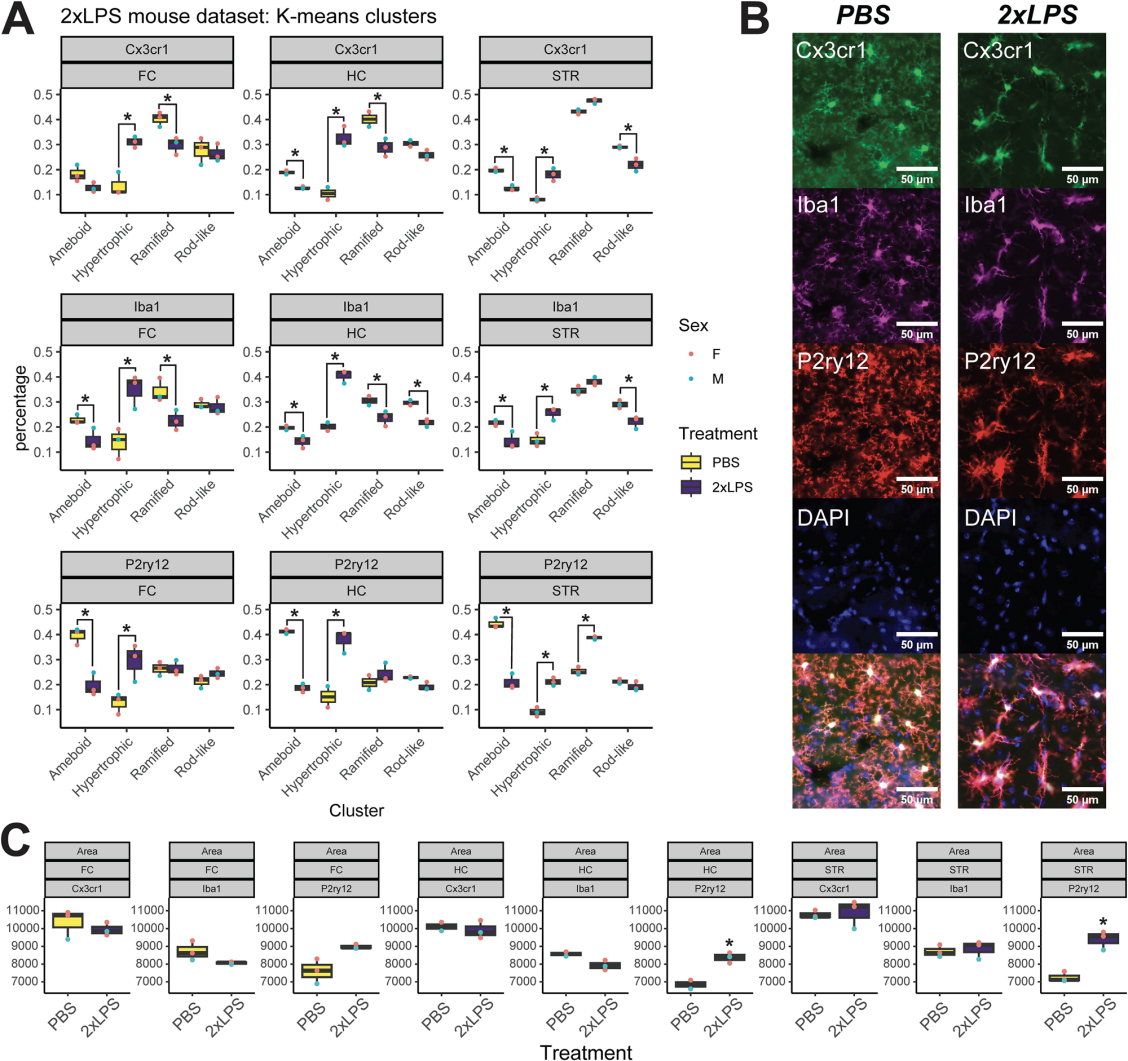
Analysis of morphological clusters and individual morphology measures across brain regions and antibody markers in 2xLPS dataset. ***A***, LPS-induced shifts in morphological populations across antibodies and within brain regions (*p < 0.05; Bonferroni). ***B***, Immunofluorescent images of the same microglial cells stained with Cx3cr1, Iba1, and P2ry12 in PBS and 2xLPS conditions. Scale bars, 50 µm. ***C***, LPS-induced changes in cell area across antibodies and within brain regions (*p < 0.05; Bonferroni) See Extended Data Figure 4-1 and Extended Data Tables 4-1–4-4 for additional data.

To highlight the utility of MicrogliaMorphologyR for assessing changes in cluster membership, we fit statistical models (see Materials and Methods) to test for LPS-induced morphological population shifts using the “stats_cluster.animal” function. Analysis of deviance (Type II Wald chi-square tests) on the models revealed significant contributions of cluster, treatment, and antibody interactions for all three brain regions [FC: *X*^2^(6, *n* = 6) = 17.676, Pr(>Chisq) = 0.007; HC: *X*^2^(6, *n* = 6) = 50.684, Pr(>Chisq) = 3.428 × 10^−9^; STR: *X*^2^(6, *n* = 6) = 77.402, Pr(>Chisq) = 1.228 × 10^−14^; Extended Data Table 4-1]. Using our toolset, we were able to characterize morphological population shifts within brain regions using different microglial markers in our experimental mouse model. Within the hippocampus and striatum, LPS-induced decreases in ameboid cluster membership and increases in hypertrophic cluster membership were maintained across all three microglia antibodies, while within the frontal cortex, changes in ameboid cluster membership were only seen in Iba1 and P2ry12-stained datasets (Fig. 4*A*, Extended Data Table 4-2). LPS-induced changes in ramified and rod-like cluster membership were mostly maintained between Cx3cr1- and Iba1-stained datasets, compared with changes in the P2ry12-stained dataset (Fig. 4*A*, Extended Data Table 4-2). Antibody-specific differences were also apparent upon examination of the immunofluorescent images for each of the antibodies, where in the baseline PBS condition, P2ry12 distribution was less concentrated in the cell bodies and more spread throughout the cell branches (Fig. 4*B*).

10.1523/ENEURO.0014-24.2024.f4-1 Figure 4-1 E*x*tended *analysis of 2xLPS dataset.* (A) Cells from dataset visualized in PCs 1-2 space and colored by different experimental variables. (B) Elbow plot depicting percentage of the variance in dataset explained by each Principal Component. (C) LPS-induced shifts in morphological populations across subregions and antibodies. Download Figure 4-1, TIF file.

10.1523/ENEURO.0014-24.2024.t4-1 Table 4-1 Analysis of Deviance (Type II Wald chisquare tests) on models fit for each brain region separately: percentage ∼ Cluster*Treatment*Antibody + (1|MouseID). Significance denoted at Pr(>Chisq) < 0.05, related to Fig. 4A. Download Table 4-1, DOC file.

10.1523/ENEURO.0014-24.2024.t4-2 Table 4-2 Tests between treatments across clusters and antibodies (∼Treatment|Cluster|Antibody), bonferroni-corrected for each brain region. Significance denoted at adjusted p-values (or q-values) < 0.05, related to Fig. 4A. Download Table 4-2, DOC file.

10.1523/ENEURO.0014-24.2024.t4-3 Table 4-3 Analysis of Deviance (Type II Wald chisquare tests) on models fit for each brain region separately for area measure: Value ∼ Treatment*Antibody + (1|MouseID). Significance denoted at Pr(>Chisq) < 0.05, related to Fig. 4C. Download Table 4-3, DOC file.

10.1523/ENEURO.0014-24.2024.t4-4 Table 4-4 Tests between treatments across antibodies (∼Treatment|Antibody), Bonferroni-corrected for each brain region. Significance denoted at adjusted p-values (or q-values) < 0.05, related to Fig. 4C. Download Table 4-4, DOC file.

To highlight the utility of MicrogliaMorphologyR for assessing changes in individual morphology measures, we fit statistical models (see Materials and Methods) to test for LPS-induced shifts in cell area as an example using the “stats_morphologymeasures.animal” function. Analysis of deviance (Type II Wald chi-square tests) on the models revealed significant contributions of treatment and antibody interactions for all three brain regions [FC: *X*^2^(2, *n* = 6) = 46.489, Pr(>Chisq) = 8.035 × 10^−11^; HC: *X*^2^(2, *n* = 6) = 189.851, Pr(>Chisq) = 5.949 × 10^−42^; STR: *X*^2^(2, *n* = 6) = 255.691, Pr(>Chisq) = 3.002 × 10^−56^; Extended Data Table 4-3]. Within the hippocampus and striatum, we observed LPS-induced increases in area for P2ry12-stained datasets that were not present in Cx3cr1- and Iba1-stained datasets (Fig. 4*C*, Extended Data Table 4-4). Taken together, our findings from the cluster-level and morphology measure-level analyses show that different antibodies produce different patterns of morphology changes but all work effectively with our toolset.

## Discussion

### MicrogliaMorphology and MicrogliaMorphologyR, a high-throughput pipeline to characterize microglia morphological states at a single-cell resolution

Microglia exhibit a dynamic range of morphologies including, but not limited to, ramified, ameboid, rod-like, and hypertrophic forms that are highly context specific and often rapidly changing in response to local environmental cues (Savage et al., 2019; Paolicelli et al., 2022; Reddaway et al., 2023). There has been a concerted effort as a field to move away from dualistic characterization of all microglia as “resting” or “activated,” which is often described in terms of morphological differences, and toward a clearer understanding and appreciation for the heterogeneous “states” of microglia that coexist in the brain in any given context (Dubbelaar et al., 2018; Paolicelli et al., 2022). In line with these efforts, there have been many recently published tools that classify and analyze microglia morphological subpopulations in an automated and high-throughput manner (York et al., 2018; Salamanca et al., 2019; Clarke et al., 2021; Leyh et al., 2021; Colombo et al., 2022; Melo et al., 2023; Reddaway et al., 2023; van Weering et al., 2023).

Using our toolset, MicrogliaMorphology and MicrogliaMorphologyR, we take a data-informed approach to characterize different populations of microglia morphologies and to statistically model how membership across all morphological states dynamically changes in experimental contexts and across brain regions in an automated and high-throughput manner, which offers a great advantage over more labor-intensive morphological approaches which employ manual categorizations of cells or assessment of individual measures of morphology rather than morphological states (Torres-Platas et al., 2014; Young and Morrison, 2018). Furthermore, the ColorByCluster feature within MicrogliaMorphology and functions within MicrogliaMorphologyR facilitate comparisons of morphological measures across clusters and together provide a thorough validation for verifying cluster identities both visually and analytically compared with existing tools. We demonstrate that MicrogliaMorphology and MicrogliaMorphologyR are able to reproducibly detect both subtle and pronounced changes in microglia morphology and together provide a robust method to characterize morphological states across a wide range of experimental and disease models. While our dataset was too underpowered to quantify sex differences in morphology, our tool could also be used to explore known sex differences in microglia in various contexts such as early brain development (Sullivan and Ciernia, 2022).

Importantly, we made both MicrogliaMorphology and MicrogliaMorphologyR free and open source resources. Our toolset only relies on software that is open source and freely available to download, and all relevant materials including input images, data, and supporting code used in this study are available on the OSF website at https://osf.io/8t5c2/. We have also included all of the single-cell images that were generated for this study at the OSF link, which with further processing can provide a unique, benchmarking dataset for researchers interested in applying other approaches such as machine learning methods to classify microglia morphology. The ImageJ and R code underlying both MicrogliaMorphology and MicrogliaMorphologyR, as well as the R code for the analysis demonstrated in this paper, are all available through GitHub repositories at https://github.com/ciernialab/MicrogliaMorphology and https://github.com/ciernialab/MicrogliaMorphologyR in the hopes that the larger research community can openly share troubleshooting tips, benefit from discussion, and continue to expand upon our work and develop our toolsets for broader use.

### MicrogliaMorphology and MicrogliaMorphologyR can be applied to quantify morphology from datasets stained for 3 different microglia markers

Cx3cr1, Iba1, and P2ry12 are all antibody markers that are commonly used to visualize and study various aspects of microglia including morphology. Of these three markers, P2ry12 is the most microglia specific, as both Cx3cr1 and Iba1 also label other macrophages (Paolicelli et al., 2022). While all three of these markers are considered “homeostatic” markers, their expression can change in disease-associated and inflammatory states (Kenkhuis et al., 2022) and infiltration of peripheral macrophages into the brain in diseases like Parkinson's disease and multiple sclerosis (Prinz and Priller, 2017) could complicate the analysis of morphology in datasets stained with nonspecific markers such as Cx3cr1 and Iba1. Thus, careful consideration of morphological markers should be taken depending on the experimental context in which microglia are being studied (Paolicelli et al., 2022). In our analyses of LPS-induced shifts in morphological populations and changes in individual morphology measures from sample-matched Cx3cr1, Iba1, and P2ry12-stained images, we found that P2ry12 showed unique differences in the percentage of morphological populations present across brain regions, the directionality of shifts across morphological populations, and the specific morphological features such as cell area that change with LPS administration (Fig. 4*A*,*C*). However, the objective of generating the triple-labeled datasets in this study was to provide a proof-of-concept for the application of MicrogliaMorphology and MicrogliaMorphologyR to different microglia antibodies. Thus, the preliminary findings presented here should be further explored in a dataset with sufficient sample size to be able to more comprehensively characterize and compare marker-specific effects.

### Future directions

To classify microglia by their morphological characteristics in any approach, hard cutoffs are used to define where one class starts and the next begins. This type of binning of morphologies is limited in that microglia are inherently dynamic and more realistically exist along a continuum of morphological forms. To represent the dynamic nature of microglia morphology, we also demonstrate that MicrogliaMorphology and MicrogliaMorphologyR can be optionally integrated with a soft clustering approach using fuzzy *k*-means clustering, which is similar in concept and algorithm to *k*-means clustering. Soft clustering approaches such as fuzzy *k*-means clustering not only yield final cluster (or class) identities for every cell but also membership scores of belonging to any given cluster. This allows for additional characterization of high-scoring cells within each cluster (i.e., quintessential “rod-like”, “ameboid”, “hypertrophic”, or “ramified” cells), cells with more ambiguous identities (e.g., a cell that is 5% rod-like, 5% ameboid, 45% hypertrophic, and 45% ramified), and other cases that the user might be interested in which might be informative for their specific dataset. Fuzzy *k*-means also assigns a final hard cluster assignment for every cell based on the class with the highest membership score, so the user can also use these final assignments as input for downstream analysis. While we used the hard cluster assignments for the analysis in this paper, we provide an example of using the soft clustering assignments from fuzzy *k*-means to analyze just the high-scoring cells for each morphological class in our GitHub page for MicrogliaMorphologyR. While we used *k*-means clustering approaches in this study, our toolset is highly flexible and can also be integrated with other clustering approaches such as hierarchical clustering or Gaussian mixture models.

Microglia have long been known as a highly heterogeneous cell type as defined by their morphology, electrophysiological properties, transcriptomic profiles, and surface expression of immune markers (Hammond et al., 2019; Li et al., 2019; Masuda et al., 2020; Paolicelli et al., 2022). Context-specific regulation of morphology further emphasizes the need to probe microglial phenotypes from multiple angles in conjunction with morphology to gain more clarity on the relationship between microglial form and function (Dubbelaar et al., 2018; Paolicelli et al., 2022). While the majority of studies of microglia morphology have yielded observational insights into the range of forms present in various contexts, only a few (Parakalan et al., 2012; Madry et al., 2018, p. 1; Adrian et al., 2023) have actually explored how different morphological states directly contribute to microglial function in the brain. The rise of single-cell sequencing technologies has provided vast new insight into the molecular mechanisms that shape heterogeneous microglial responses and has granted us a better understanding of microglial “states” in homeostatic, developmental, and disease-relevant contexts (Hammond et al., 2019; Li et al., 2019; Masuda et al., 2020). However, transcriptomic characterization alone does not capture the diversity of changes that microglia exhibit, and we still lack a direct understanding of whether morphologically different microglia populations are transcriptomically distinct. Parakalan et al. (2012) is one such study that directly explored these relationships by identifying over 2,000 differentially expressed genes with unique sets of biological functions between ameboid and ramified microglia laser-dissected and pooled from rat brains. However, while it is agreed upon that microglia exhibit a wide range of morphological forms across various biological contexts, it is still unclear whether we can transcriptomically define the heterogeneous morphological states that exist outside of the “resting” versus “activated” morphological dichotomy and in what ways these transcriptomic signatures relate to microglial function.

The advent of spatially resolved transcriptomics and development of methods for integrating multiple data modalities has opened new avenues to explore these relationships more directly. The ability to map morphologically classified microglia back to their spatial locations in their original input images using the complementary ColorByCluster functions in MicrogliaMorphology and MicrogliaMorphologyR allows for not only the visual verification and exploration of morphological cluster identity across tissue sections but also facilitates the direct integration of spatial transcriptomics data to morphological data at a cellular resolution. Our toolset serves as a resource that can complement new tools and approaches such as spatial transcriptomics to answer questions about the relationship between microglia morphology and microglia function more directly.

### Requirements of MicrogliaMorphology and MicrogliaMorphologyR

Our toolset requires the use of 2D image forms (EDF or single-plane 2D images) and depends on some user input to determine dataset-specific parameters for MicrogliaMorphology. Introductory skills coding in the R language are also necessary to be able to use MicrogliaMorphologyR, and larger computational resources may be required for the analysis of larger datasets. However, many of these requirements exist for alternative approaches for morphology analysis that have been presented as well.

## Synthesis

Reviewing Editor: Arvind Kumar, KTH Royal Institute of Technology Decisions are customarily a result of the Reviewing Editor and the peer reviewers coming together and discussing their recommendations until a consensus is reached. When revisions are invited, a fact-based synthesis statement explaining their decision and outlining what is needed to prepare a revision will be listed below. The following reviewer(s) agreed to reveal their identity: Stefano Garofalo.

Synthesis

In this manuscript authors have presented a pipeline for the analysis of morphology of microglia. From the perspective of a methodological paper, reviewers think that authors have done a great job in presenting the method. But the scientific results in the manuscript are rather weak.

We think that the authors should revise the manuscript with more focus on the method details, validation and versatility. For instance, authors should provide a comparison of their pipeline with an human expert evaluation of the morphologies.

The scientific results can be retained in the manuscript but only as a proof of concept or examples of the applicability of the tool. But if authors want to keep them as results - it wont work given the sample size. Moreover, if it is a methodology paper, authors should not describe markers related to microglial functions. If they do, then they need to give some minimal discussion on the topic.

I have appended the comments from the reviewers. Please consider them in the revision of the manuscript.

We look forward to reading the revised version of the manuscript.

Reviewer #1

The authors compiled a morphology analysis pipeline into an accessible and fully open-source ImageJ macro and R package to evaluate the heterogeneity of microglia phenotype in various experimental models. The methods are very well described, making the reproducibility of the analysis higher in comparison to previously published papers. This is a methodological paper that describes a new tool to analyze microglial morphology, useful also in combination with other techniques such as spatial transcriptomic.

From the point of view of the methodological study, it is well constructed and described, while from biological point of view results too weak. The number of animals is too few to distinguish between male and female, and the markers used (cx3cr1, p2yr12 and iba1) did not add information to the analysis, opening more questions. The authors should stress more about the methods and less the biological results.

Minor:

In the introduction, most of the information refers to reviews and not original papers. The authors should improve the references.

Reviewer #2

The manuscript describes the creation and use of a new open source tool for the automated classification of microglia based on morphological features. The authors have built in an analysis pipeline that allows for not only the detection of microglia of different morphology but also clustering of different morphological phenotypes.

The analysis package that the authors have put together seems to function well and is provides and excellent approach to quantifying microglia morphology in a way that is both high throughput and will increase consistency. I can imagine that many labs will benefit from this package.

The manuscript itself is also very nicely written and clear to follow. I only have a couple of comments.

1. Would users be able to refine/modify the classifiers used in the MicrogliaMorphologyR pipeline should they decide to further subdivide morphological classifications? As a hypothetical example, recent work has identified a population of "exhausted-like" microglia with enriched expression of stress- and inflammation-related genes (10.1016/j.immuni.2023.12.001). While this work did not assess morphological characteristics of these microglia, provided that they differed based on a subset of the 27 morphological characteristics considered in the MicrogliaMorphology pipelines, would future users be able to modify the classifiers to accommodate new microglia states?

This might also be important to consider when examining different species. The authors do mention that human and mouse morphologies are similar but perhaps in other species the differences might be more apparent and require modification of the classifiers.

I don't believe this comment requires any experimental action but perhaps the authors could clarify this point.

2. Although the package itself has been well validated as being able to identify differences induced by LPS administration, it does not appear as though a basic validation of the accuracy of the morphological detection has been performed in comparison to some ground truth, (i.e. a trained human). Could the authors include a comparison from a few images showing the morphological comparison performed by an experienced rater to that of the detection software to determine the accuracy of classification.

## Author Response

April 29, 2024 Dear eNeuro editors and reviewers, Thank you for the opportunity to submit to eNeuro a revised draft of our manuscript titled: Development of a high-throughput pipeline to characterize microglia morphological states at a single-cell resolution. We are very grateful for the detailed feedback and appreciate the time and effort that was dedicated to help us improve our manuscript. We were able to update our manuscript to incorporate changes that the suggestions provided by the reviewers, and we have highlighted them below. Below is a point-by-point response to the reviewersâ€{trade mark, serif} suggestions and concerns. The line numbers correspond to the No Markup version of the attached Manuscript Revision Word .docx file. Manuscript Instructions Extended Data should be labeled as Figure 1-1, Figure 1-2, Table 1-1, etc., so they indicate which figure they are supporting (i.e. Extended Data table supporting Figure 5 labeled as Figure 5-1). This change is not necessary at this stage, but will be if your paper receives a revise decision. * Response: We have updated the figure and table labels throughout the text. New legend names and descriptions can be found in lines 665-755. Reviewer #1 Comments The authors compiled a morphology analysis pipeline into an accessible and fully open-source ImageJ macro and R package to evaluate the heterogeneity of microglia phenotype in various experimental models. The methods are very well described, making the reproducibility of the analysis higher in comparison to previously published papers. This is a methodological paper that describes a new tool to analyze microglial morphology, useful also in combination with other techniques such as spatial transcriptomic. From the point of view of the methodological study, it is well constructed and described, while from biological point of view results too weak. The number of animals is too few to distinguish between male and female, and the markers used (cx3cr1, p2yr12 and iba1) did not add information to the analysis, opening more questions. The authors should stress more about the methods and less the biological results. This is a confidential document and must not be discussed with others, forwarded in any form, or posted on websites without the express written consent of eNeuro. * Response: To emphasize the results as more of a use-case and example of the applicability of our tool, we updated the text in the discussion: 1) Lines 575-576: changed the subheading of the section where we talk about antibody-specific differences from â€œChoice of markers affects morphology analysisâ€ to â€œMicrogliaMorphology and MicrogliaMorphologyR can be applied to quantify morphology from datasets stained for 3 different microglia markersâ€; 2) removed sentences within this subsection summarizing Alzheimerâ€{trade mark, serif}s disease-specific differences in expression of microglial markers near pathological hallmarks; 3) Added text in lines 591-595 emphasizing that the objective of the triple labeled dataset was to show that our toolset could be applied to different microglia markers and that the findings presented are preliminary and should be followed up on in a study with sufficient sample size. We also updated the analysis to better reflect direct comparisons between antibodies and reduced the text in the results section by highlighting just one individual morphology measure, area, rather than 3 and updated Figure 4C to reflect this change - these changes can all be found in the â€œStatistical analysis of microglia markersâ€ subsection of the Methods section in lines 303-327. In the introduction, most of the information refers to reviews and not original papers. The authors should improve the references. * Response: We have updated the references to original research papers throughout the introduction section in lines 36-127 and have highlighted new additions in yellow. Reviewer #2 Comments The manuscript describes the creation and use of a new open source tool for the automated classification of microglia based on morphological features. The authors have built in an analysis pipeline that allows for not only the detection of microglia of different morphology but also clustering of different morphological phenotypes. The analysis package that the authors have put together seems to function well and is provides and excellent approach to quantifying microglia morphology in a way that is both high throughput and will increase consistency. I can imagine that many labs will benefit from this package. The manuscript itself is also very nicely written and clear to follow. I only have a couple of comments. This is a confidential document and must not be discussed with others, forwarded in any form, or posted on websites without the express written consent of eNeuro. 1) Would users be able to refine/modify the classifiers used in the MicrogliaMorphologyR pipeline should they decide to further subdivide morphological classifications? As a hypothetical example, recent work has identified a population of exhausted-like microglia with enriched expression of stress- and inflammation-related genes (10.1016/j.immuni.2023.12.001). While this work did not assess morphological characteristics of these microglia, provided that they differed based on a subset of the 27 morphological characteristics considered in the MicrogliaMorphology pipelines, would future users be able to modify the classifiers to accommodate new microglia states? * Response: Yes, the analysis will always be dataset-specific and users can modify the classifiers to accommodate any number of microglial states that are appropriate for their study. Our toolset just offers different ways to facilitate comparisons of morphological measures across clusters more thoroughly and to verify suspected cluster identities both visually and analytically. However, the final numbers of clusters determined for a dataset is ultimately up to the user, and the hope is that the user will use the metrics available through our toolset to more objectively guide their decision-making. This might also be important to consider when examining different species. The authors do mention that human and mouse morphologies are similar but perhaps in other species the differences might be more apparent and require modification of the classifiers. I don't believe this comment requires any experimental action but perhaps the authors could clarify this point. * Response: As long as the cells can be parsed individually in MicrogliaMorphology, images of microglia (or any other cell type) from any species can be analyzed. 2) Although the package itself has been well validated as being able to identify differences induced by LPS administration, it does not appear as though a basic validation of the accuracy of the morphological detection has been performed in comparison to some ground truth, (i.e. a trained human). Could the authors include a comparison from a few images showing the morphological comparison performed by an experienced rater to that of the detection software to determine the accuracy of classification. * Response: We are interpreting this recommendation to be about validating the clustering specifically, and not the measurement of individual morphology features. This recommendation assumes that human raters can provide an objective truth to be used as the standard to compare against, but purely manual classification is a very subjective This is a confidential document and must not be discussed with others, forwarded in any form, or posted on websites without the express written consent of eNeuro. process as effective blinding is not possible, no actual guidelines for manual classification of microglia morphology exist that are agreed upon by the field, and there are many inter-rater biases present which make the process highly subjective (Reddaway et al., 2023). In reality, one â€œtrainedâ€ human does not have the same opinions as another, and could easily be driven by motives to quantify the morphological subtype they want and expect to see within any given experiment. Moreover, it has been shown that humans rely on context and group differences within images to classify morphology and perform poorly when given the task of classifying morphologies of single cell images without the context of the larger image (Yao et al., 2019). Additionally, â€œexpertsâ€ assign different morphological classifications to the same reconstructions of neurons from their own studies when grading cells using a web-based interactive classification system, highlighting that they engage in their own, mutually inconsistent classification schemes based on grading criteria. (DeFelipe et al., 2013) Because microglia morphology is dynamic and there are intermediate states that would be challenging to classify subjectively, there would likely be a lot of disagreement between manual ratings for these more ambiguous cases. Therefore, using a clustering approach to mathematically process the morphological data our toolset produces to determine cluster membership is a more scientifically sound approach. While questions about how many clusters there are in a dataset and their biological interpretation is, of course, important, we would like to emphasize that this is the application of the output of our tool and not the tool itself. Our toolset just offers different ways to facilitate comparisons of morphological measures across clusters more thoroughly and to verify suspected cluster identities both visually and analytically. However, the final numbers of clusters determined for a dataset and their interpretation is ultimately up to the user, and the hope is that the user will use the metrics available through our toolset to more objectively guide their decision-making. Taken together, we donâ€{trade mark, serif}t think that having a single experienced rater classifying a few images as suggested by the reviewer would provide anything scientifically useful for the reasons listed. If the reviewer still thinks that a human-rater experiment is absolutely necessary for publication, please advise us on what specifically you would like us to do to address this comment. Works cited: * Reddaway J, Richardson PE, Bevan RJ, Stoneman J, Palombo M. Microglial morphometric analysis: so many options, so little consistency. Front Neuroinform. 2023 Aug 10;17:1211188. doi: 10.3389/fninf.2023.1211188. PMID: 37637472; PMCID: PMC10448193. * Yao K, Rochman ND, Sun SX. Cell Type Classification and Unsupervised Morphological Phenotyping From Low-Resolution Images Using Deep Learning. Sci Rep. 2019 Sep 17;9(1):13467. doi: 10.1038/s41598-019- 50010-9. PMID: 31530889; PMCID: PMC6749053. * DeFelipe J, LÃ³pez-Cruz PL, Benavides-Piccione R, Bielza C, LarraÃ{plus minus}aga P, Anderson S, Burkhalter A, Cauli B, FairÃ{copyright, serif}n A, Feldmeyer D, Fishell G, This is a confidential document and must not be discussed with others, forwarded in any form, or posted on websites without the express written consent of eNeuro. Fitzpatrick D, Freund TF, GonzÃ¡lez-Burgos G, Hestrin S, Hill S, Hof PR, Huang J, Jones EG, Kawaguchi Y, KisvÃ¡rday Z, Kubota Y, Lewis DA, MarÃn O, Markram H, McBain CJ, Meyer HS, Monyer H, Nelson SB, Rockland K, Rossier J, Rubenstein JL, Rudy B, Scanziani M, Shepherd GM, Sherwood CC, Staiger JF, TamÃ¡s G, Thomson A, Wang Y, Yuste R, Ascoli GA. New insights into the classification and nomenclature of cortical GABAergic interneurons. Nat Rev Neurosci. 2013 Mar;14(3):202-16. doi: 10.1038/nrn3444. Epub 2013 Feb 6. PMID: 23385869; PMCID: PMC3619199. We look forward to hearing from you regarding our updated submission and are happy to respond to any further questions and suggestions you may have. Thank you very much! Sincerely, Corresponding author of manuscript
