# Supplementary material for: Development of a High-Throughput Pipeline to Characterize Microglia Morphological States at a Single-Cell Resolution
Source: eNeuro. 2024 Jul 26;11(7):ENEURO.0014-24.2024. doi: 10.1523/ENEURO.0014-24.2024 (PMC11289588; doi:10.1523/ENEURO.0014-24.2024)
Supplement: Table 2-1 — Pearson’s correlation of principal components and p-values for correlations, related to Fig. 2C. Download Table 2-1, DOC file. [file eneuro-11-ENEURO.0014-24.2024-s001.doc]

| **PC_a** | **PC_b** | **correlation** | **pvalues** |
| --- | --- | --- | --- |
| PC1_2D | PC1_2D | 1 | NA |
| PC1_2D | PC1_3D | 0.964718444036363 | 7.0778938265903e-12 |
| PC1_2D | PC1_EDF | 0.931263798607638 | 2.53033283215132e-09 |
| PC1_2D | PC2_2D | 0.0894725244048934 | 0.707569183209019 |
| PC1_2D | PC2_3D | 0.433775235032866 | 0.0560280579596322 |
| PC1_2D | PC2_EDF | 0.33818087495069 | 0.144734461673298 |
| PC1_3D | PC1_2D | 0.964718444036363 | 7.0778938265903e-12 |
| PC1_3D | PC1_3D | 1 | NA |
| PC1_3D | PC1_EDF | 0.956800137725298 | 4.25330881625996e-11 |
| PC1_3D | PC2_2D | 0.16137551709721 | 0.496694318565953 |
| PC1_3D | PC2_3D | 0.46034138978036 | 0.041105747450644 |
| PC1_3D | PC2_EDF | 0.387624984967488 | 0.0912855174299179 |
| PC1_EDF | PC1_2D | 0.931263798607638 | 2.53033283215132e-09 |
| PC1_EDF | PC1_3D | 0.956800137725298 | 4.25330881625996e-11 |
| PC1_EDF | PC1_EDF | 1 | NA |
| PC1_EDF | PC2_2D | 0.122270130499778 | 0.607578000024524 |
| PC1_EDF | PC2_3D | 0.450312701282719 | 0.0463256299903181 |
| PC1_EDF | PC2_EDF | 0.247276243273875 | 0.293220667237261 |
| PC2_2D | PC1_2D | 0.0894725244048934 | 0.707569183209019 |
| PC2_2D | PC1_3D | 0.16137551709721 | 0.496694318565953 |
| PC2_2D | PC1_EDF | 0.122270130499778 | 0.607578000024524 |
| PC2_2D | PC2_2D | 1 | NA |
| PC2_2D | PC2_3D | 0.667698241087472 | 0.00129592879981777 |
| PC2_2D | PC2_EDF | 0.459143970211989 | 0.0417038987709291 |
| PC2_3D | PC1_2D | 0.433775235032866 | 0.0560280579596322 |
| PC2_3D | PC1_3D | 0.46034138978036 | 0.041105747450644 |
| PC2_3D | PC1_EDF | 0.450312701282719 | 0.0463256299903181 |
| PC2_3D | PC2_2D | 0.667698241087472 | 0.00129592879981777 |
| PC2_3D | PC2_3D | 1 | NA |
| PC2_3D | PC2_EDF | 0.718113524629044 | 0.000362770419291447 |
| PC2_EDF | PC1_2D | 0.33818087495069 | 0.144734461673298 |
| PC2_EDF | PC1_3D | 0.387624984967488 | 0.0912855174299179 |
| PC2_EDF | PC1_EDF | 0.247276243273875 | 0.293220667237261 |
| PC2_EDF | PC2_2D | 0.459143970211989 | 0.0417038987709291 |
| PC2_EDF | PC2_3D | 0.718113524629044 | 0.000362770419291447 |
| PC2_EDF | PC2_EDF | 1 | NA |
