# Supplementary material for: Development of a High-Throughput Pipeline to Characterize Microglia Morphological States at a Single-Cell Resolution
Source: eNeuro. 2024 Jul 26;11(7):ENEURO.0014-24.2024. doi: 10.1523/ENEURO.0014-24.2024 (PMC11289588; doi:10.1523/ENEURO.0014-24.2024)
Supplement: Table 2-2 — Spearman’s correlation of morphology measures to principal components and p-values for correlations, related to Fig. 2-1C. Download Table 2-2, DOC file. [file eneuro-11-ENEURO.0014-24.2024-s002.doc]

| **measure** | **PC** | **correlation** | **pvalues** |
| --- | --- | --- | --- |
| # of branches | PC1 | 0.988840909824356 | 0 |
| # of branches | PC2 | -0.073764229992519 | 0.575397736900022 |
| # of branches | PC3 | 0.0366875597551338 | 0.780784937967498 |
| # of branches | PC4 | 0.000583665723377128 | 0.996468615547526 |
| # of branches | PC5 | -0.103030896979001 | 0.43340771423406 |
| # of end point voxels | PC1 | 0.967541038529119 | 0 |
| # of end point voxels | PC2 | -0.0941662028870446 | 0.474203173534294 |
| # of end point voxels | PC3 | -0.0378445732642969 | 0.774051761932836 |
| # of end point voxels | PC4 | 0.0501997133594056 | 0.703273100664661 |
| # of end point voxels | PC5 | -0.105519574866334 | 0.422321024304819 |
| # of junction voxels | PC1 | 0.944039165213608 | 0 |
| # of junction voxels | PC2 | -0.0776253831201344 | 0.555508699061563 |
| # of junction voxels | PC3 | -0.193354740553804 | 0.138814082475258 |
| # of junction voxels | PC4 | 0.111838360786044 | 0.394910314013419 |
| # of junction voxels | PC5 | -0.293242183064998 | 0.0229718133604879 |
| # of junctions | PC1 | 0.977396757565732 | 0 |
| # of junctions | PC2 | -0.0581906247641894 | 0.658753083234275 |
| # of junctions | PC3 | 0.0830460564599301 | 0.528150878487275 |
| # of junctions | PC4 | -0.0251612591550843 | 0.848659918105135 |
| # of junctions | PC5 | -0.0978369844936371 | 0.457065901375996 |
| # of quadruple points | PC1 | 0.801777690707537 | 1.37667655053519e-14 |
| # of quadruple points | PC2 | 0.00385719144664357 | 0.976665790985342 |
| # of quadruple points | PC3 | -0.551290526762072 | 4.99796985709011e-06 |
| # of quadruple points | PC4 | 0.0264534249213839 | 0.840986353759135 |
| # of quadruple points | PC5 | 0.11735648901467 | 0.371852823862512 |
| # of slab voxels | PC1 | 0.9156408906255 | 0 |
| # of slab voxels | PC2 | 0.166189669129703 | 0.204417381401716 |
| # of slab voxels | PC3 | 0.107727361179712 | 0.412622604506434 |
| # of slab voxels | PC4 | -0.149267831895129 | 0.254998468020504 |
| # of slab voxels | PC5 | -0.0438467309624932 | 0.739397901002707 |
| # of triple points | PC1 | 0.960108244516891 | 0 |
| # of triple points | PC2 | -0.0687996465440923 | 0.601441378821487 |
| # of triple points | PC3 | 0.143077680464574 | 0.275455991974684 |
| # of triple points | PC4 | -0.036874830766963 | 0.7796940239731 |
| # of triple points | PC5 | -0.11404500827701 | 0.385590614888543 |
| Average branch length | PC1 | -0.558707602073154 | 3.49684059575139e-06 |
| Average branch length | PC2 | 0.698565269112771 | 5.44301048677198e-10 |
| Average branch length | PC3 | 0.0308415495619277 | 0.815041051481383 |
| Average branch length | PC4 | -0.371405675743503 | 0.00348164411000829 |
| Average branch length | PC5 | -0.00567328774628788 | 0.965684798475595 |
| Maximum branch length | PC1 | 0.275753788961727 | 0.0329588468724735 |
| Maximum branch length | PC2 | 0.867333619599362 | 0 |
| Maximum branch length | PC3 | 0.0550229267504001 | 0.676271286766178 |
| Maximum branch length | PC4 | 0.361011536067903 | 0.00459972923774599 |
| Maximum branch length | PC5 | 0.118215924442526 | 0.368336346897159 |
