# Supplementary material for: Development of a High-Throughput Pipeline to Characterize Microglia Morphological States at a Single-Cell Resolution
Source: eNeuro. 2024 Jul 26;11(7):ENEURO.0014-24.2024. doi: 10.1523/ENEURO.0014-24.2024 (PMC11289588; doi:10.1523/ENEURO.0014-24.2024)
Supplement: Table 3-1 — Spearman’s correlation of morphology measures and p-values for correlations, related to Fig. 3A. Download Table 3-1, DOC file. [file eneuro-11-ENEURO.0014-24.2024-s003.doc]

| **measure_a** | **measure_b** | **correlation** | **pvalues** |
| --- | --- | --- | --- |
| # of branches | Area | 0.870279704126133 | 0 |
| # of branches | Average.branch.length | -0.433767010493458 | 0 |
| # of branches | Circularity | 0.207119938078143 | 0 |
| # of branches | Density.of.foreground.pixels.in.hull.area | -0.513017120537881 | 0 |
| # of branches | Diameter.of.bounding.circle | 0.789589398502023 | 0 |
| # of branches | Foreground.pixels | 0.817589383397924 | 0 |
| # of branches | Height.of.bounding.rectangle | 0.735697247813546 | 0 |
| # of branches | Max.min.radii.from.circle.s.center.of.mass | -0.0756022797274397 | 0 |
| # of branches | Max.min.radii.from.hull.s.center.of.mass | -0.172567823467187 | 0 |
| # of branches | Maximum.branch.length | 0.133276502790138 | 0 |
| # of branches | Maximum.radius.from.circle.s.center.of.mass | 0.789589414020039 | 0 |
| # of branches | Maximum.radius.from.hull.s.center.of.mass | 0.765056734010344 | 0 |
| # of branches | Maximum.span.across.hull | 0.784546049993071 | 0 |
| # of branches | Mean.radius | 0.812800848879536 | 0 |
| # of branches | Mean.radius.from.circle.s.center.of.mass | 0.807692778171319 | 0 |
| # of branches | Perimeter | 0.839723536731482 | 0 |
| # of branches | Relative.variation..CV..in.radii.from.circle.s.center.of.mass | -0.0812344759568278 | 0 |
| # of branches | Relative.variation..CV..in.radii.from.hull.s.center.of.mass | -0.174815258890409 | 0 |
| # of branches | Span.ratio.of.hull..major.minor.axis. | -0.154483825251242 | 0 |
| # of branches | Width.of.bounding.rectangle | 0.738863465186592 | 0 |
| # of branches | X..of.branches | 1 | NA |
| # of branches | X..of.end.point.voxels | 0.942551427766579 | 0 |
| # of branches | X..of.junction.voxels | 0.975748475646093 | 0 |
| # of branches | X..of.junctions | 0.997017388820973 | 0 |
| # of branches | X..of.quadruple.points | 0.534953754491433 | 0 |
| # of branches | X..of.slab.voxels | 0.946410437832116 | 0 |
| # of branches | X..of.triple.points | 0.988819548852999 | 0 |
| # of end point voxels | Area | 0.869457539709727 | 0 |
| # of end point voxels | Average.branch.length | -0.372592354828206 | 0 |
| # of end point voxels | Circularity | 0.190468511709512 | 0 |
| # of end point voxels | Density.of.foreground.pixels.in.hull.area | -0.58274135634984 | 0 |
| # of end point voxels | Diameter.of.bounding.circle | 0.792375089966396 | 0 |
| # of end point voxels | Foreground.pixels | 0.786466955556312 | 0 |
| # of end point voxels | Height.of.bounding.rectangle | 0.737591951396698 | 0 |
| # of end point voxels | Max.min.radii.from.circle.s.center.of.mass | -0.0743487130466927 | 0 |
| # of end point voxels | Max.min.radii.from.hull.s.center.of.mass | -0.164853772808831 | 0 |
| # of end point voxels | Maximum.branch.length | 0.162512582113566 | 0 |
| # of end point voxels | Maximum.radius.from.circle.s.center.of.mass | 0.792375078876259 | 0 |
| # of end point voxels | Maximum.radius.from.hull.s.center.of.mass | 0.768661387437399 | 0 |
| # of end point voxels | Maximum.span.across.hull | 0.787306840510699 | 0 |
| # of end point voxels | Mean.radius | 0.816080774706233 | 0 |
| # of end point voxels | Mean.radius.from.circle.s.center.of.mass | 0.811013639493219 | 0 |
| # of end point voxels | Perimeter | 0.840969897283505 | 0 |
| # of end point voxels | Relative.variation..CV..in.radii.from.circle.s.center.of.mass | -0.0801277458070065 | 0 |
| # of end point voxels | Relative.variation..CV..in.radii.from.hull.s.center.of.mass | -0.167003183078594 | 0 |
| # of end point voxels | Span.ratio.of.hull..major.minor.axis. | -0.144815177738081 | 0 |
| # of end point voxels | Width.of.bounding.rectangle | 0.739228165292799 | 0 |
| # of end point voxels | X..of.branches | 0.942551427766579 | 0 |
| # of end point voxels | X..of.end.point.voxels | 1 | NA |
| # of end point voxels | X..of.junction.voxels | 0.89925052298832 | 0 |
| # of end point voxels | X..of.junctions | 0.916715836315564 | 0 |
| # of end point voxels | X..of.quadruple.points | 0.505676665368971 | 0 |
| # of end point voxels | X..of.slab.voxels | 0.908488654218388 | 0 |
| # of end point voxels | X..of.triple.points | 0.907366417865909 | 0 |
| # of junction voxels | Area | 0.839445153300049 | 0 |
| # of junction voxels | Average.branch.length | -0.423978438347027 | 0 |
| # of junction voxels | Circularity | 0.205437338354286 | 0 |
| # of junction voxels | Density.of.foreground.pixels.in.hull.area | -0.477652880487385 | 0 |
| # of junction voxels | Diameter.of.bounding.circle | 0.759950567231289 | 0 |
| # of junction voxels | Foreground.pixels | 0.796515216484863 | 0 |
| # of junction voxels | Height.of.bounding.rectangle | 0.708855435202185 | 0 |
| # of junction voxels | Max.min.radii.from.circle.s.center.of.mass | -0.0724729731857548 | 0 |
| # of junction voxels | Max.min.radii.from.hull.s.center.of.mass | -0.170133456016743 | 0 |
| # of junction voxels | Maximum.branch.length | 0.121305425787818 | 0 |
| # of junction voxels | Maximum.radius.from.circle.s.center.of.mass | 0.759950593551556 | 0 |
| # of junction voxels | Maximum.radius.from.hull.s.center.of.mass | 0.735871624404429 | 0 |
| # of junction voxels | Maximum.span.across.hull | 0.755035732603252 | 0 |
| # of junction voxels | Mean.radius | 0.782386551315737 | 0 |
| # of junction voxels | Mean.radius.from.circle.s.center.of.mass | 0.777274940710946 | 0 |
| # of junction voxels | Perimeter | 0.809014323783874 | 0 |
| # of junction voxels | Relative.variation..CV..in.radii.from.circle.s.center.of.mass | -0.0778151373035474 | 0 |
| # of junction voxels | Relative.variation..CV..in.radii.from.hull.s.center.of.mass | -0.172116100222339 | 0 |
| # of junction voxels | Span.ratio.of.hull..major.minor.axis. | -0.153760419348038 | 0 |
| # of junction voxels | Width.of.bounding.rectangle | 0.712186672842358 | 0 |
| # of junction voxels | X..of.branches | 0.975748475646093 | 0 |
| # of junction voxels | X..of.end.point.voxels | 0.89925052298832 | 0 |
| # of junction voxels | X..of.junction.voxels | 1 | NA |
| # of junction voxels | X..of.junctions | 0.975373834335616 | 0 |
| # of junction voxels | X..of.quadruple.points | 0.566306070492533 | 0 |
| # of junction voxels | X..of.slab.voxels | 0.91894219855478 | 0 |
| # of junction voxels | X..of.triple.points | 0.961960408035309 | 0 |
| # of junctions | Area | 0.858435371551824 | 0 |
| # of junctions | Average.branch.length | -0.4426342519225 | 0 |
| # of junctions | Circularity | 0.206342255204931 | 0 |
| # of junctions | Density.of.foreground.pixels.in.hull.area | -0.493628106946815 | 0 |
| # of junctions | Diameter.of.bounding.circle | 0.778556229598562 | 0 |
| # of junctions | Foreground.pixels | 0.811933560693732 | 0 |
| # of junctions | Height.of.bounding.rectangle | 0.72536613377572 | 0 |
| # of junctions | Max.min.radii.from.circle.s.center.of.mass | -0.0744421585584609 | 0 |
| # of junctions | Max.min.radii.from.hull.s.center.of.mass | -0.170727231975621 | 0 |
| # of junctions | Maximum.branch.length | 0.12508120763662 | 0 |
| # of junctions | Maximum.radius.from.circle.s.center.of.mass | 0.778556251650582 | 0 |
| # of junctions | Maximum.radius.from.hull.s.center.of.mass | 0.754243606597065 | 0 |
| # of junctions | Maximum.span.across.hull | 0.773617158466239 | 0 |
| # of junctions | Mean.radius | 0.801244894193699 | 0 |
| # of junctions | Mean.radius.from.circle.s.center.of.mass | 0.796223772201085 | 0 |
| # of junctions | Perimeter | 0.828100387362316 | 0 |
| # of junctions | Relative.variation..CV..in.radii.from.circle.s.center.of.mass | -0.0799299892184914 | 0 |
| # of junctions | Relative.variation..CV..in.radii.from.hull.s.center.of.mass | -0.173127404669658 | 0 |
| # of junctions | Span.ratio.of.hull..major.minor.axis. | -0.153007344962941 | 0 |
| # of junctions | Width.of.bounding.rectangle | 0.728675143292752 | 0 |
| # of junctions | X..of.branches | 0.997017388820973 | 0 |
| # of junctions | X..of.end.point.voxels | 0.916715836315564 | 0 |
| # of junctions | X..of.junction.voxels | 0.975373834335616 | 0 |
| # of junctions | X..of.junctions | 1 | NA |
| # of junctions | X..of.quadruple.points | 0.510366328281602 | 0 |
| # of junctions | X..of.slab.voxels | 0.939866608750159 | 0 |
| # of junctions | X..of.triple.points | 0.995194276004749 | 0 |
| # of quadruple points | Area | 0.444177626435394 | 0 |
| # of quadruple points | Average.branch.length | -0.189562208447833 | 0 |
| # of quadruple points | Circularity | 0.13712095139189 | 0 |
| # of quadruple points | Density.of.foreground.pixels.in.hull.area | -0.224095894668508 | 0 |
| # of quadruple points | Diameter.of.bounding.circle | 0.392363500415536 | 0 |
| # of quadruple points | Foreground.pixels | 0.434501393968193 | 0 |
| # of quadruple points | Height.of.bounding.rectangle | 0.369950428462283 | 0 |
| # of quadruple points | Max.min.radii.from.circle.s.center.of.mass | -0.0496596889679138 | 0 |
| # of quadruple points | Max.min.radii.from.hull.s.center.of.mass | -0.113993328764662 | 0 |
| # of quadruple points | Maximum.branch.length | 0.0656415924224468 | 0 |
| # of quadruple points | Maximum.radius.from.circle.s.center.of.mass | 0.392363474425529 | 0 |
| # of quadruple points | Maximum.radius.from.hull.s.center.of.mass | 0.378119042536725 | 0 |
| # of quadruple points | Maximum.span.across.hull | 0.389184927696926 | 0 |
| # of quadruple points | Mean.radius | 0.406453425453363 | 0 |
| # of quadruple points | Mean.radius.from.circle.s.center.of.mass | 0.403505131682858 | 0 |
| # of quadruple points | Perimeter | 0.422963686030874 | 0 |
| # of quadruple points | Relative.variation..CV..in.radii.from.circle.s.center.of.mass | -0.0535952699101407 | 0 |
| # of quadruple points | Relative.variation..CV..in.radii.from.hull.s.center.of.mass | -0.112687497958581 | 0 |
| # of quadruple points | Span.ratio.of.hull..major.minor.axis. | -0.107702905736618 | 0 |
| # of quadruple points | Width.of.bounding.rectangle | 0.373939945314504 | 0 |
| # of quadruple points | X..of.branches | 0.534953754491433 | 0 |
| # of quadruple points | X..of.end.point.voxels | 0.505676665368971 | 0 |
| # of quadruple points | X..of.junction.voxels | 0.566306070492533 | 0 |
| # of quadruple points | X..of.junctions | 0.510366328281602 | 0 |
| # of quadruple points | X..of.quadruple.points | 1 | NA |
| # of quadruple points | X..of.slab.voxels | 0.514115898532003 | 0 |
| # of quadruple points | X..of.triple.points | 0.432868414014579 | 0 |
| # of slab voxels | Area | 0.966050167454338 | 0 |
| # of slab voxels | Average.branch.length | -0.14699280746351 | 0 |
| # of slab voxels | Circularity | 0.172776339234026 | 0 |
| # of slab voxels | Density.of.foreground.pixels.in.hull.area | -0.543713366263801 | 0 |
| # of slab voxels | Diameter.of.bounding.circle | 0.895507903459788 | 0 |
| # of slab voxels | Foreground.pixels | 0.918853570942563 | 0 |
| # of slab voxels | Height.of.bounding.rectangle | 0.82624586206343 | 0 |
| # of slab voxels | Max.min.radii.from.circle.s.center.of.mass | -0.0614638778079768 | 0 |
| # of slab voxels | Max.min.radii.from.hull.s.center.of.mass | -0.140637236250492 | 0 |
| # of slab voxels | Maximum.branch.length | 0.326362806993915 | 0 |
| # of slab voxels | Maximum.radius.from.circle.s.center.of.mass | 0.89550790003489 | 0 |
| # of slab voxels | Maximum.radius.from.hull.s.center.of.mass | 0.874049642396535 | 0 |
| # of slab voxels | Maximum.span.across.hull | 0.889998470086133 | 0 |
| # of slab voxels | Mean.radius | 0.917077976987415 | 0 |
| # of slab voxels | Mean.radius.from.circle.s.center.of.mass | 0.913046330745086 | 0 |
| # of slab voxels | Perimeter | 0.943107383631784 | 0 |
| # of slab voxels | Relative.variation..CV..in.radii.from.circle.s.center.of.mass | -0.066821506822694 | 0 |
| # of slab voxels | Relative.variation..CV..in.radii.from.hull.s.center.of.mass | -0.141286099890714 | 0 |
| # of slab voxels | Span.ratio.of.hull..major.minor.axis. | -0.135348085352828 | 0 |
| # of slab voxels | Width.of.bounding.rectangle | 0.826295305004462 | 0 |
| # of slab voxels | X..of.branches | 0.946410437832116 | 0 |
| # of slab voxels | X..of.end.point.voxels | 0.908488654218388 | 0 |
| # of slab voxels | X..of.junction.voxels | 0.91894219855478 | 0 |
| # of slab voxels | X..of.junctions | 0.939866608750159 | 0 |
| # of slab voxels | X..of.quadruple.points | 0.514115898532003 | 0 |
| # of slab voxels | X..of.slab.voxels | 1 | NA |
| # of slab voxels | X..of.triple.points | 0.931010915891002 | 0 |
| # of triple points | Area | 0.853544778474226 | 0 |
| # of triple points | Average.branch.length | -0.445747123805815 | 0 |
| # of triple points | Circularity | 0.200959210435835 | 0 |
| # of triple points | Density.of.foreground.pixels.in.hull.area | -0.494772311663357 | 0 |
| # of triple points | Diameter.of.bounding.circle | 0.775494829230918 | 0 |
| # of triple points | Foreground.pixels | 0.805767632003723 | 0 |
| # of triple points | Height.of.bounding.rectangle | 0.721872591194821 | 0 |
| # of triple points | Max.min.radii.from.circle.s.center.of.mass | -0.0727966604969464 | 0 |
| # of triple points | Max.min.radii.from.hull.s.center.of.mass | -0.166490020566609 | 0 |
| # of triple points | Maximum.branch.length | 0.124310363722102 | 0 |
| # of triple points | Maximum.radius.from.circle.s.center.of.mass | 0.775494856673958 | 0 |
| # of triple points | Maximum.radius.from.hull.s.center.of.mass | 0.751473133519457 | 0 |
| # of triple points | Maximum.span.across.hull | 0.770680152346776 | 0 |
| # of triple points | Mean.radius | 0.797746854624585 | 0 |
| # of triple points | Mean.radius.from.circle.s.center.of.mass | 0.792807938161369 | 0 |
| # of triple points | Perimeter | 0.824090258998828 | 0 |
| # of triple points | Relative.variation..CV..in.radii.from.circle.s.center.of.mass | -0.0780817141275291 | 0 |
| # of triple points | Relative.variation..CV..in.radii.from.hull.s.center.of.mass | -0.169260998705826 | 0 |
| # of triple points | Span.ratio.of.hull..major.minor.axis. | -0.147974181544751 | 0 |
| # of triple points | Width.of.bounding.rectangle | 0.724797172204361 | 0 |
| # of triple points | X..of.branches | 0.988819548852999 | 0 |
| # of triple points | X..of.end.point.voxels | 0.907366417865909 | 0 |
| # of triple points | X..of.junction.voxels | 0.961960408035309 | 0 |
| # of triple points | X..of.junctions | 0.995194276004749 | 0 |
| # of triple points | X..of.quadruple.points | 0.432868414014579 | 0 |
| # of triple points | X..of.slab.voxels | 0.931010915891002 | 0 |
| # of triple points | X..of.triple.points | 1 | NA |
| Area | Area | 1 | NA |
| Area | Average.branch.length | -0.0206323350770382 | 1.74512126251969e-05 |
| Area | Circularity | 0.11799651403009 | 0 |
| Area | Density.of.foreground.pixels.in.hull.area | -0.591693786328655 | 0 |
| Area | Diameter.of.bounding.circle | 0.942640371692751 | 0 |
| Area | Foreground.pixels | 0.936889720219534 | 0 |
| Area | Height.of.bounding.rectangle | 0.861337322159709 | 0 |
| Area | Max.min.radii.from.circle.s.center.of.mass | -0.0716584138387166 | 0 |
| Area | Max.min.radii.from.hull.s.center.of.mass | -0.11204046262038 | 0 |
| Area | Maximum.branch.length | 0.408422090897812 | 0 |
| Area | Maximum.radius.from.circle.s.center.of.mass | 0.942640365280099 | 0 |
| Area | Maximum.radius.from.hull.s.center.of.mass | 0.926377165578504 | 0 |
| Area | Maximum.span.across.hull | 0.936708204921127 | 0 |
| Area | Mean.radius | 0.967025005525981 | 0 |
| Area | Mean.radius.from.circle.s.center.of.mass | 0.964235773039085 | 0 |
| Area | Perimeter | 0.986255958307987 | 0 |
| Area | Relative.variation..CV..in.radii.from.circle.s.center.of.mass | -0.0799183605024835 | 0 |
| Area | Relative.variation..CV..in.radii.from.hull.s.center.of.mass | -0.114062615445787 | 0 |
| Area | Span.ratio.of.hull..major.minor.axis. | -0.105196557602272 | 0 |
| Area | Width.of.bounding.rectangle | 0.862730790795378 | 0 |
| Area | X..of.branches | 0.870279704126133 | 0 |
| Area | X..of.end.point.voxels | 0.869457539709727 | 0 |
| Area | X..of.junction.voxels | 0.839445153300049 | 0 |
| Area | X..of.junctions | 0.858435371551824 | 0 |
| Area | X..of.quadruple.points | 0.444177626435394 | 0 |
| Area | X..of.slab.voxels | 0.966050167454338 | 0 |
| Area | X..of.triple.points | 0.853544778474226 | 0 |
| Average branch length | Area | -0.0206323350770382 | 1.74512126251969e-05 |
| Average branch length | Average.branch.length | 1 | NA |
| Average branch length | Circularity | -0.152760277248602 | 0 |
| Average branch length | Density.of.foreground.pixels.in.hull.area | 0.1053288364984 | 0 |
| Average branch length | Diameter.of.bounding.circle | 0.0284822413698051 | 3.02949354491489e-09 |
| Average branch length | Foreground.pixels | 0.0226699855714346 | 2.36388383267894e-06 |
| Average branch length | Height.of.bounding.rectangle | 0.00790891590222104 | 0.0996968535358138 |
| Average branch length | Max.min.radii.from.circle.s.center.of.mass | 0.0637727346513757 | 0 |
| Average branch length | Max.min.radii.from.hull.s.center.of.mass | 0.142659617066935 | 0 |
| Average branch length | Maximum.branch.length | 0.504813694982283 | 0 |
| Average branch length | Maximum.radius.from.circle.s.center.of.mass | 0.0284821570848907 | 3.02981773003808e-09 |
| Average branch length | Maximum.radius.from.hull.s.center.of.mass | 0.0438273982821756 | 0 |
| Average branch length | Maximum.span.across.hull | 0.029007264441201 | 1.54738932778287e-09 |
| Average branch length | Mean.radius | 0.016598660378233 | 0.000549534287713982 |
| Average branch length | Mean.radius.from.circle.s.center.of.mass | 0.0209241328826347 | 1.32466954356758e-05 |
| Average branch length | Perimeter | 0.00646466864283797 | 0.178404891468142 |
| Average branch length | Relative.variation..CV..in.radii.from.circle.s.center.of.mass | 0.0653539291382807 | 0 |
| Average branch length | Relative.variation..CV..in.radii.from.hull.s.center.of.mass | 0.145774984330698 | 0 |
| Average branch length | Span.ratio.of.hull..major.minor.axis. | 0.0994586066627968 | 0 |
| Average branch length | Width.of.bounding.rectangle | 0.00250623097234891 | 0.601885266995704 |
| Average branch length | X..of.branches | -0.433767010493458 | 0 |
| Average branch length | X..of.end.point.voxels | -0.372592354828206 | 0 |
| Average branch length | X..of.junction.voxels | -0.423978438347027 | 0 |
| Average branch length | X..of.junctions | -0.4426342519225 | 0 |
| Average branch length | X..of.quadruple.points | -0.189562208447833 | 0 |
| Average branch length | X..of.slab.voxels | -0.14699280746351 | 0 |
| Average branch length | X..of.triple.points | -0.445747123805815 | 0 |
| Circularity | Area | 0.11799651403009 | 0 |
| Circularity | Average.branch.length | -0.152760277248602 | 0 |
| Circularity | Circularity | 1 | NA |
| Circularity | Density.of.foreground.pixels.in.hull.area | 0.0576105829081519 | 0 |
| Circularity | Diameter.of.bounding.circle | -0.170881195372636 | 0 |
| Circularity | Foreground.pixels | 0.163757557881063 | 0 |
| Circularity | Height.of.bounding.rectangle | -0.0222646466204714 | 3.5672135085818e-06 |
| Circularity | Max.min.radii.from.circle.s.center.of.mass | -0.543503902945391 | 0 |
| Circularity | Max.min.radii.from.hull.s.center.of.mass | -0.726428256858663 | 0 |
| Circularity | Maximum.branch.length | -0.128054708194659 | 0 |
| Circularity | Maximum.radius.from.circle.s.center.of.mass | -0.170881211069446 | 0 |
| Circularity | Maximum.radius.from.hull.s.center.of.mass | -0.191415456717985 | 0 |
| Circularity | Maximum.span.across.hull | -0.177867452511137 | 0 |
| Circularity | Mean.radius | -0.092033317667078 | 0 |
| Circularity | Mean.radius.from.circle.s.center.of.mass | -0.105973771584636 | 0 |
| Circularity | Perimeter | -0.0244818351411776 | 3.45372162202651e-07 |
| Circularity | Relative.variation..CV..in.radii.from.circle.s.center.of.mass | -0.539787135482064 | 0 |
| Circularity | Relative.variation..CV..in.radii.from.hull.s.center.of.mass | -0.698244208304774 | 0 |
| Circularity | Span.ratio.of.hull..major.minor.axis. | -0.816411507650905 | 0 |
| Circularity | Width.of.bounding.rectangle | 0.0254549907074434 | 1.16118255455433e-07 |
| Circularity | X..of.branches | 0.207119938078143 | 0 |
| Circularity | X..of.end.point.voxels | 0.190468511709512 | 0 |
| Circularity | X..of.junction.voxels | 0.205437338354286 | 0 |
| Circularity | X..of.junctions | 0.206342255204931 | 0 |
| Circularity | X..of.quadruple.points | 0.13712095139189 | 0 |
| Circularity | X..of.slab.voxels | 0.172776339234026 | 0 |
| Circularity | X..of.triple.points | 0.200959210435835 | 0 |
| Density of foreground pixels in hull area | Area | -0.591693786328655 | 0 |
| Density of foreground pixels in hull area | Average.branch.length | 0.1053288364984 | 0 |
| Density of foreground pixels in hull area | Circularity | 0.0576105829081519 | 0 |
| Density of foreground pixels in hull area | Density.of.foreground.pixels.in.hull.area | 1 | NA |
| Density of foreground pixels in hull area | Diameter.of.bounding.circle | -0.584260781989121 | 0 |
| Density of foreground pixels in hull area | Foreground.pixels | -0.294129819085501 | 0 |
| Density of foreground pixels in hull area | Height.of.bounding.rectangle | -0.528770182187893 | 0 |
| Density of foreground pixels in hull area | Max.min.radii.from.circle.s.center.of.mass | 0.088182584705824 | 0 |
| Density of foreground pixels in hull area | Max.min.radii.from.hull.s.center.of.mass | 0.0323491784264062 | 1.63384861195937e-11 |
| Density of foreground pixels in hull area | Maximum.branch.length | -0.289697635440003 | 0 |
| Density of foreground pixels in hull area | Maximum.radius.from.circle.s.center.of.mass | -0.584260774770198 | 0 |
| Density of foreground pixels in hull area | Maximum.radius.from.hull.s.center.of.mass | -0.58499747490767 | 0 |
| Density of foreground pixels in hull area | Maximum.span.across.hull | -0.580329595271922 | 0 |
| Density of foreground pixels in hull area | Mean.radius | -0.606322001674621 | 0 |
| Density of foreground pixels in hull area | Mean.radius.from.circle.s.center.of.mass | -0.606552935664553 | 0 |
| Density of foreground pixels in hull area | Perimeter | -0.600104148412333 | 0 |
| Density of foreground pixels in hull area | Relative.variation..CV..in.radii.from.circle.s.center.of.mass | 0.0906240595631344 | 0 |
| Density of foreground pixels in hull area | Relative.variation..CV..in.radii.from.hull.s.center.of.mass | 0.0246487033532335 | 2.87305720547693e-07 |
| Density of foreground pixels in hull area | Span.ratio.of.hull..major.minor.axis. | 0.0101756065285584 | 0.0341596101606025 |
| Density of foreground pixels in hull area | Width.of.bounding.rectangle | -0.5265647743259 | 0 |
| Density of foreground pixels in hull area | X..of.branches | -0.513017120537881 | 0 |
| Density of foreground pixels in hull area | X..of.end.point.voxels | -0.58274135634984 | 0 |
| Density of foreground pixels in hull area | X..of.junction.voxels | -0.477652880487385 | 0 |
| Density of foreground pixels in hull area | X..of.junctions | -0.493628106946815 | 0 |
| Density of foreground pixels in hull area | X..of.quadruple.points | -0.224095894668508 | 0 |
| Density of foreground pixels in hull area | X..of.slab.voxels | -0.543713366263801 | 0 |
| Density of foreground pixels in hull area | X..of.triple.points | -0.494772311663357 | 0 |
| Diameter of bounding circle | Area | 0.942640371692751 | 0 |
| Diameter of bounding circle | Average.branch.length | 0.0284822413698051 | 3.02949354491489e-09 |
| Diameter of bounding circle | Circularity | -0.170881195372636 | 0 |
| Diameter of bounding circle | Density.of.foreground.pixels.in.hull.area | -0.584260781989121 | 0 |
| Diameter of bounding circle | Diameter.of.bounding.circle | 1 | NA |
| Diameter of bounding circle | Foreground.pixels | 0.872854423718717 | 0 |
| Diameter of bounding circle | Height.of.bounding.rectangle | 0.857929871701467 | 0 |
| Diameter of bounding circle | Max.min.radii.from.circle.s.center.of.mass | 0.129281564885551 | 0 |
| Diameter of bounding circle | Max.min.radii.from.hull.s.center.of.mass | 0.130181464500985 | 0 |
| Diameter of bounding circle | Maximum.branch.length | 0.444959471364668 | 0 |
| Diameter of bounding circle | Maximum.radius.from.circle.s.center.of.mass | 0.999999999934184 | 0 |
| Diameter of bounding circle | Maximum.radius.from.hull.s.center.of.mass | 0.986233418655774 | 0 |
| Diameter of bounding circle | Maximum.span.across.hull | 0.99892043144843 | 0 |
| Diameter of bounding circle | Mean.radius | 0.98571657143719 | 0 |
| Diameter of bounding circle | Mean.radius.from.circle.s.center.of.mass | 0.986960792662937 | 0 |
| Diameter of bounding circle | Perimeter | 0.981649841055076 | 0 |
| Diameter of bounding circle | Relative.variation..CV..in.radii.from.circle.s.center.of.mass | 0.134220143801569 | 0 |
| Diameter of bounding circle | Relative.variation..CV..in.radii.from.hull.s.center.of.mass | 0.12536980701226 | 0 |
| Diameter of bounding circle | Span.ratio.of.hull..major.minor.axis. | 0.17362240010263 | 0 |
| Diameter of bounding circle | Width.of.bounding.rectangle | 0.839699209103542 | 0 |
| Diameter of bounding circle | X..of.branches | 0.789589398502023 | 0 |
| Diameter of bounding circle | X..of.end.point.voxels | 0.792375089966396 | 0 |
| Diameter of bounding circle | X..of.junction.voxels | 0.759950567231289 | 0 |
| Diameter of bounding circle | X..of.junctions | 0.778556229598562 | 0 |
| Diameter of bounding circle | X..of.quadruple.points | 0.392363500415536 | 0 |
| Diameter of bounding circle | X..of.slab.voxels | 0.895507903459788 | 0 |
| Diameter of bounding circle | X..of.triple.points | 0.775494829230918 | 0 |
| Foreground pixels | Area | 0.936889720219534 | 0 |
| Foreground pixels | Average.branch.length | 0.0226699855714346 | 2.36388383267894e-06 |
| Foreground pixels | Circularity | 0.163757557881063 | 0 |
| Foreground pixels | Density.of.foreground.pixels.in.hull.area | -0.294129819085501 | 0 |
| Foreground pixels | Diameter.of.bounding.circle | 0.872854423718717 | 0 |
| Foreground pixels | Foreground.pixels | 1 | NA |
| Foreground pixels | Height.of.bounding.rectangle | 0.801264259706169 | 0 |
| Foreground pixels | Max.min.radii.from.circle.s.center.of.mass | -0.0483298542598424 | 0 |
| Foreground pixels | Max.min.radii.from.hull.s.center.of.mass | -0.119957257992999 | 0 |
| Foreground pixels | Maximum.branch.length | 0.366530761459073 | 0 |
| Foreground pixels | Maximum.radius.from.circle.s.center.of.mass | 0.8728544171938 | 0 |
| Foreground pixels | Maximum.radius.from.hull.s.center.of.mass | 0.853351500222111 | 0 |
| Foreground pixels | Maximum.span.across.hull | 0.867656936831152 | 0 |
| Foreground pixels | Mean.radius | 0.891688139195409 | 0 |
| Foreground pixels | Mean.radius.from.circle.s.center.of.mass | 0.888290131097613 | 0 |
| Foreground pixels | Perimeter | 0.916936349817696 | 0 |
| Foreground pixels | Relative.variation..CV..in.radii.from.circle.s.center.of.mass | -0.057021514894003 | 0 |
| Foreground pixels | Relative.variation..CV..in.radii.from.hull.s.center.of.mass | -0.125568302914974 | 0 |
| Foreground pixels | Span.ratio.of.hull..major.minor.axis. | -0.119435579325684 | 0 |
| Foreground pixels | Width.of.bounding.rectangle | 0.803645529629612 | 0 |
| Foreground pixels | X..of.branches | 0.817589383397924 | 0 |
| Foreground pixels | X..of.end.point.voxels | 0.786466955556312 | 0 |
| Foreground pixels | X..of.junction.voxels | 0.796515216484863 | 0 |
| Foreground pixels | X..of.junctions | 0.811933560693732 | 0 |
| Foreground pixels | X..of.quadruple.points | 0.434501393968193 | 0 |
| Foreground pixels | X..of.slab.voxels | 0.918853570942563 | 0 |
| Foreground pixels | X..of.triple.points | 0.805767632003723 | 0 |
| Height of bounding rectangle | Area | 0.861337322159709 | 0 |
| Height of bounding rectangle | Average.branch.length | 0.00790891590222104 | 0.0996968535358138 |
| Height of bounding rectangle | Circularity | -0.0222646466204714 | 3.5672135085818e-06 |
| Height of bounding rectangle | Density.of.foreground.pixels.in.hull.area | -0.528770182187893 | 0 |
| Height of bounding rectangle | Diameter.of.bounding.circle | 0.857929871701467 | 0 |
| Height of bounding rectangle | Foreground.pixels | 0.801264259706169 | 0 |
| Height of bounding rectangle | Height.of.bounding.rectangle | 1 | NA |
| Height of bounding rectangle | Max.min.radii.from.circle.s.center.of.mass | 0.00955047232938573 | 0.0468065197448362 |
| Height of bounding rectangle | Max.min.radii.from.hull.s.center.of.mass | -0.00551373316201941 | 0.251078786963749 |
| Height of bounding rectangle | Maximum.branch.length | 0.38708702199184 | 0 |
| Height of bounding rectangle | Maximum.radius.from.circle.s.center.of.mass | 0.857929838115099 | 0 |
| Height of bounding rectangle | Maximum.radius.from.hull.s.center.of.mass | 0.845415790727287 | 0 |
| Height of bounding rectangle | Maximum.span.across.hull | 0.853959377663987 | 0 |
| Height of bounding rectangle | Mean.radius | 0.863868831867782 | 0 |
| Height of bounding rectangle | Mean.radius.from.circle.s.center.of.mass | 0.864000005268545 | 0 |
| Height of bounding rectangle | Perimeter | 0.872397446073664 | 0 |
| Height of bounding rectangle | Relative.variation..CV..in.radii.from.circle.s.center.of.mass | 0.00631380963432988 | 0.188752248208777 |
| Height of bounding rectangle | Relative.variation..CV..in.radii.from.hull.s.center.of.mass | -0.00954747280364566 | 0.0468756146818299 |
| Height of bounding rectangle | Span.ratio.of.hull..major.minor.axis. | 0.0131968385085662 | 0.0060117630511316 |
| Height of bounding rectangle | Width.of.bounding.rectangle | 0.557135801190955 | 0 |
| Height of bounding rectangle | X..of.branches | 0.735697247813546 | 0 |
| Height of bounding rectangle | X..of.end.point.voxels | 0.737591951396698 | 0 |
| Height of bounding rectangle | X..of.junction.voxels | 0.708855435202185 | 0 |
| Height of bounding rectangle | X..of.junctions | 0.72536613377572 | 0 |
| Height of bounding rectangle | X..of.quadruple.points | 0.369950428462283 | 0 |
| Height of bounding rectangle | X..of.slab.voxels | 0.82624586206343 | 0 |
| Height of bounding rectangle | X..of.triple.points | 0.721872591194821 | 0 |
| Max/min radii from circle's center of mass | Area | -0.0716584138387166 | 0 |
| Max/min radii from circle's center of mass | Average.branch.length | 0.0637727346513757 | 0 |
| Max/min radii from circle's center of mass | Circularity | -0.543503902945391 | 0 |
| Max/min radii from circle's center of mass | Density.of.foreground.pixels.in.hull.area | 0.088182584705824 | 0 |
| Max/min radii from circle's center of mass | Diameter.of.bounding.circle | 0.129281564885551 | 0 |
| Max/min radii from circle's center of mass | Foreground.pixels | -0.0483298542598424 | 0 |
| Max/min radii from circle's center of mass | Height.of.bounding.rectangle | 0.00955047232938573 | 0.0468065197448362 |
| Max/min radii from circle's center of mass | Max.min.radii.from.circle.s.center.of.mass | 1 | NA |
| Max/min radii from circle's center of mass | Max.min.radii.from.hull.s.center.of.mass | 0.71510197509986 | 0 |
| Max/min radii from circle's center of mass | Maximum.branch.length | 0.0488444629304575 | 0 |
| Max/min radii from circle's center of mass | Maximum.radius.from.circle.s.center.of.mass | 0.129281649109372 | 0 |
| Max/min radii from circle's center of mass | Maximum.radius.from.hull.s.center.of.mass | 0.123894429483278 | 0 |
| Max/min radii from circle's center of mass | Maximum.span.across.hull | 0.142014172002031 | 0 |
| Max/min radii from circle's center of mass | Mean.radius | 0.0238980474204883 | 6.51581973709625e-07 |
| Max/min radii from circle's center of mass | Mean.radius.from.circle.s.center.of.mass | 0.0243386501354637 | 4.04090853667327e-07 |
| Max/min radii from circle's center of mass | Perimeter | 0.0085269977417882 | 0.0758995581391022 |
| Max/min radii from circle's center of mass | Relative.variation..CV..in.radii.from.circle.s.center.of.mass | 0.914378095282022 | 0 |
| Max/min radii from circle's center of mass | Relative.variation..CV..in.radii.from.hull.s.center.of.mass | 0.643588503871307 | 0 |
| Max/min radii from circle's center of mass | Span.ratio.of.hull..major.minor.axis. | 0.60678956151938 | 0 |
| Max/min radii from circle's center of mass | Width.of.bounding.rectangle | -0.0309920175832701 | 1.0982592613118e-10 |
| Max/min radii from circle's center of mass | X..of.branches | -0.0756022797274397 | 0 |
| Max/min radii from circle's center of mass | X..of.end.point.voxels | -0.0743487130466927 | 0 |
| Max/min radii from circle's center of mass | X..of.junction.voxels | -0.0724729731857548 | 0 |
| Max/min radii from circle's center of mass | X..of.junctions | -0.0744421585584609 | 0 |
| Max/min radii from circle's center of mass | X..of.quadruple.points | -0.0496596889679138 | 0 |
| Max/min radii from circle's center of mass | X..of.slab.voxels | -0.0614638778079768 | 0 |
| Max/min radii from circle's center of mass | X..of.triple.points | -0.0727966604969464 | 0 |
| Max/min radii from hull's center of mass | Area | -0.11204046262038 | 0 |
| Max/min radii from hull's center of mass | Average.branch.length | 0.142659617066935 | 0 |
| Max/min radii from hull's center of mass | Circularity | -0.726428256858663 | 0 |
| Max/min radii from hull's center of mass | Density.of.foreground.pixels.in.hull.area | 0.0323491784264062 | 1.63384861195937e-11 |
| Max/min radii from hull's center of mass | Diameter.of.bounding.circle | 0.130181464500985 | 0 |
| Max/min radii from hull's center of mass | Foreground.pixels | -0.119957257992999 | 0 |
| Max/min radii from hull's center of mass | Height.of.bounding.rectangle | -0.00551373316201941 | 0.251078786963749 |
| Max/min radii from hull's center of mass | Max.min.radii.from.circle.s.center.of.mass | 0.71510197509986 | 0 |
| Max/min radii from hull's center of mass | Max.min.radii.from.hull.s.center.of.mass | 1 | NA |
| Max/min radii from hull's center of mass | Maximum.branch.length | 0.122058260162056 | 0 |
| Max/min radii from hull's center of mass | Maximum.radius.from.circle.s.center.of.mass | 0.13018152393187 | 0 |
| Max/min radii from hull's center of mass | Maximum.radius.from.hull.s.center.of.mass | 0.17436320543333 | 0 |
| Max/min radii from hull's center of mass | Maximum.span.across.hull | 0.141819014106739 | 0 |
| Max/min radii from hull's center of mass | Mean.radius | 0.0233247293327423 | 1.19856057523293e-06 |
| Max/min radii from hull's center of mass | Mean.radius.from.circle.s.center.of.mass | 0.0391096886717688 | 4.44089209850063e-16 |
| Max/min radii from hull's center of mass | Perimeter | -0.00481989831197671 | 0.315714548622203 |
| Max/min radii from hull's center of mass | Relative.variation..CV..in.radii.from.circle.s.center.of.mass | 0.708516981580342 | 0 |
| Max/min radii from hull's center of mass | Relative.variation..CV..in.radii.from.hull.s.center.of.mass | 0.897522857693994 | 0 |
| Max/min radii from hull's center of mass | Span.ratio.of.hull..major.minor.axis. | 0.734107027240098 | 0 |
| Max/min radii from hull's center of mass | Width.of.bounding.rectangle | -0.0452452213569109 | 0 |
| Max/min radii from hull's center of mass | X..of.branches | -0.172567823467187 | 0 |
| Max/min radii from hull's center of mass | X..of.end.point.voxels | -0.164853772808831 | 0 |
| Max/min radii from hull's center of mass | X..of.junction.voxels | -0.170133456016743 | 0 |
| Max/min radii from hull's center of mass | X..of.junctions | -0.170727231975621 | 0 |
| Max/min radii from hull's center of mass | X..of.quadruple.points | -0.113993328764662 | 0 |
| Max/min radii from hull's center of mass | X..of.slab.voxels | -0.140637236250492 | 0 |
| Max/min radii from hull's center of mass | X..of.triple.points | -0.166490020566609 | 0 |
| Maximum branch length | Area | 0.408422090897812 | 0 |
| Maximum branch length | Average.branch.length | 0.504813694982283 | 0 |
| Maximum branch length | Circularity | -0.128054708194659 | 0 |
| Maximum branch length | Density.of.foreground.pixels.in.hull.area | -0.289697635440003 | 0 |
| Maximum branch length | Diameter.of.bounding.circle | 0.444959471364668 | 0 |
| Maximum branch length | Foreground.pixels | 0.366530761459073 | 0 |
| Maximum branch length | Height.of.bounding.rectangle | 0.38708702199184 | 0 |
| Maximum branch length | Max.min.radii.from.circle.s.center.of.mass | 0.0488444629304575 | 0 |
| Maximum branch length | Max.min.radii.from.hull.s.center.of.mass | 0.122058260162056 | 0 |
| Maximum branch length | Maximum.branch.length | 1 | NA |
| Maximum branch length | Maximum.radius.from.circle.s.center.of.mass | 0.444959483822094 | 0 |
| Maximum branch length | Maximum.radius.from.hull.s.center.of.mass | 0.457897679425697 | 0 |
| Maximum branch length | Maximum.span.across.hull | 0.44350752820317 | 0 |
| Maximum branch length | Mean.radius | 0.439244538759374 | 0 |
| Maximum branch length | Mean.radius.from.circle.s.center.of.mass | 0.443304756781015 | 0 |
| Maximum branch length | Perimeter | 0.434951574921928 | 0 |
| Maximum branch length | Relative.variation..CV..in.radii.from.circle.s.center.of.mass | 0.0457970486577601 | 0 |
| Maximum branch length | Relative.variation..CV..in.radii.from.hull.s.center.of.mass | 0.128476746535101 | 0 |
| Maximum branch length | Span.ratio.of.hull..major.minor.axis. | 0.0833718748853752 | 0 |
| Maximum branch length | Width.of.bounding.rectangle | 0.376499280238273 | 0 |
| Maximum branch length | X..of.branches | 0.133276502790138 | 0 |
| Maximum branch length | X..of.end.point.voxels | 0.162512582113566 | 0 |
| Maximum branch length | X..of.junction.voxels | 0.121305425787818 | 0 |
| Maximum branch length | X..of.junctions | 0.12508120763662 | 0 |
| Maximum branch length | X..of.quadruple.points | 0.0656415924224468 | 0 |
| Maximum branch length | X..of.slab.voxels | 0.326362806993915 | 0 |
| Maximum branch length | X..of.triple.points | 0.124310363722102 | 0 |
| Maximum radius from circle's center of mass | Area | 0.942640365280099 | 0 |
| Maximum radius from circle's center of mass | Average.branch.length | 0.0284821570848907 | 3.02981773003808e-09 |
| Maximum radius from circle's center of mass | Circularity | -0.170881211069446 | 0 |
| Maximum radius from circle's center of mass | Density.of.foreground.pixels.in.hull.area | -0.584260774770198 | 0 |
| Maximum radius from circle's center of mass | Diameter.of.bounding.circle | 0.999999999934184 | 0 |
| Maximum radius from circle's center of mass | Foreground.pixels | 0.8728544171938 | 0 |
| Maximum radius from circle's center of mass | Height.of.bounding.rectangle | 0.857929838115099 | 0 |
| Maximum radius from circle's center of mass | Max.min.radii.from.circle.s.center.of.mass | 0.129281649109372 | 0 |
| Maximum radius from circle's center of mass | Max.min.radii.from.hull.s.center.of.mass | 0.13018152393187 | 0 |
| Maximum radius from circle's center of mass | Maximum.branch.length | 0.444959483822094 | 0 |
| Maximum radius from circle's center of mass | Maximum.radius.from.circle.s.center.of.mass | 1 | NA |
| Maximum radius from circle's center of mass | Maximum.radius.from.hull.s.center.of.mass | 0.986233406976135 | 0 |
| Maximum radius from circle's center of mass | Maximum.span.across.hull | 0.998920430906739 | 0 |
| Maximum radius from circle's center of mass | Mean.radius | 0.985716564888545 | 0 |
| Maximum radius from circle's center of mass | Mean.radius.from.circle.s.center.of.mass | 0.986960779888528 | 0 |
| Maximum radius from circle's center of mass | Perimeter | 0.98164983493365 | 0 |
| Maximum radius from circle's center of mass | Relative.variation..CV..in.radii.from.circle.s.center.of.mass | 0.134220237298356 | 0 |
| Maximum radius from circle's center of mass | Relative.variation..CV..in.radii.from.hull.s.center.of.mass | 0.125369835859444 | 0 |
| Maximum radius from circle's center of mass | Span.ratio.of.hull..major.minor.axis. | 0.173622408478345 | 0 |
| Maximum radius from circle's center of mass | Width.of.bounding.rectangle | 0.839699241723877 | 0 |
| Maximum radius from circle's center of mass | X..of.branches | 0.789589414020039 | 0 |
| Maximum radius from circle's center of mass | X..of.end.point.voxels | 0.792375078876259 | 0 |
| Maximum radius from circle's center of mass | X..of.junction.voxels | 0.759950593551556 | 0 |
| Maximum radius from circle's center of mass | X..of.junctions | 0.778556251650582 | 0 |
| Maximum radius from circle's center of mass | X..of.quadruple.points | 0.392363474425529 | 0 |
| Maximum radius from circle's center of mass | X..of.slab.voxels | 0.89550790003489 | 0 |
| Maximum radius from circle's center of mass | X..of.triple.points | 0.775494856673958 | 0 |
| Maximum radius from hull's center of mass | Area | 0.926377165578504 | 0 |
| Maximum radius from hull's center of mass | Average.branch.length | 0.0438273982821756 | 0 |
| Maximum radius from hull's center of mass | Circularity | -0.191415456717985 | 0 |
| Maximum radius from hull's center of mass | Density.of.foreground.pixels.in.hull.area | -0.58499747490767 | 0 |
| Maximum radius from hull's center of mass | Diameter.of.bounding.circle | 0.986233418655774 | 0 |
| Maximum radius from hull's center of mass | Foreground.pixels | 0.853351500222111 | 0 |
| Maximum radius from hull's center of mass | Height.of.bounding.rectangle | 0.845415790727287 | 0 |
| Maximum radius from hull's center of mass | Max.min.radii.from.circle.s.center.of.mass | 0.123894429483278 | 0 |
| Maximum radius from hull's center of mass | Max.min.radii.from.hull.s.center.of.mass | 0.17436320543333 | 0 |
| Maximum radius from hull's center of mass | Maximum.branch.length | 0.457897679425697 | 0 |
| Maximum radius from hull's center of mass | Maximum.radius.from.circle.s.center.of.mass | 0.986233406976135 | 0 |
| Maximum radius from hull's center of mass | Maximum.radius.from.hull.s.center.of.mass | 1 | NA |
| Maximum radius from hull's center of mass | Maximum.span.across.hull | 0.984756812102263 | 0 |
| Maximum radius from hull's center of mass | Mean.radius | 0.971536697835693 | 0 |
| Maximum radius from hull's center of mass | Mean.radius.from.circle.s.center.of.mass | 0.97725523775402 | 0 |
| Maximum radius from hull's center of mass | Perimeter | 0.96815550941462 | 0 |
| Maximum radius from hull's center of mass | Relative.variation..CV..in.radii.from.circle.s.center.of.mass | 0.121421066866228 | 0 |
| Maximum radius from hull's center of mass | Relative.variation..CV..in.radii.from.hull.s.center.of.mass | 0.1863981490794 | 0 |
| Maximum radius from hull's center of mass | Span.ratio.of.hull..major.minor.axis. | 0.172568796630723 | 0 |
| Maximum radius from hull's center of mass | Width.of.bounding.rectangle | 0.829495443755098 | 0 |
| Maximum radius from hull's center of mass | X..of.branches | 0.765056734010344 | 0 |
| Maximum radius from hull's center of mass | X..of.end.point.voxels | 0.768661387437399 | 0 |
| Maximum radius from hull's center of mass | X..of.junction.voxels | 0.735871624404429 | 0 |
| Maximum radius from hull's center of mass | X..of.junctions | 0.754243606597065 | 0 |
| Maximum radius from hull's center of mass | X..of.quadruple.points | 0.378119042536725 | 0 |
| Maximum radius from hull's center of mass | X..of.slab.voxels | 0.874049642396535 | 0 |
| Maximum radius from hull's center of mass | X..of.triple.points | 0.751473133519457 | 0 |
| Maximum span across hull | Area | 0.936708204921127 | 0 |
| Maximum span across hull | Average.branch.length | 0.029007264441201 | 1.54738932778287e-09 |
| Maximum span across hull | Circularity | -0.177867452511137 | 0 |
| Maximum span across hull | Density.of.foreground.pixels.in.hull.area | -0.580329595271922 | 0 |
| Maximum span across hull | Diameter.of.bounding.circle | 0.99892043144843 | 0 |
| Maximum span across hull | Foreground.pixels | 0.867656936831152 | 0 |
| Maximum span across hull | Height.of.bounding.rectangle | 0.853959377663987 | 0 |
| Maximum span across hull | Max.min.radii.from.circle.s.center.of.mass | 0.142014172002031 | 0 |
| Maximum span across hull | Max.min.radii.from.hull.s.center.of.mass | 0.141819014106739 | 0 |
| Maximum span across hull | Maximum.branch.length | 0.44350752820317 | 0 |
| Maximum span across hull | Maximum.radius.from.circle.s.center.of.mass | 0.998920430906739 | 0 |
| Maximum span across hull | Maximum.radius.from.hull.s.center.of.mass | 0.984756812102263 | 0 |
| Maximum span across hull | Maximum.span.across.hull | 1 | NA |
| Maximum span across hull | Mean.radius | 0.981664995212056 | 0 |
| Maximum span across hull | Mean.radius.from.circle.s.center.of.mass | 0.983148221160357 | 0 |
| Maximum span across hull | Perimeter | 0.976881218755601 | 0 |
| Maximum span across hull | Relative.variation..CV..in.radii.from.circle.s.center.of.mass | 0.148684860425952 | 0 |
| Maximum span across hull | Relative.variation..CV..in.radii.from.hull.s.center.of.mass | 0.137833805283324 | 0 |
| Maximum span across hull | Span.ratio.of.hull..major.minor.axis. | 0.1943241486818 | 0 |
| Maximum span across hull | Width.of.bounding.rectangle | 0.834505569632486 | 0 |
| Maximum span across hull | X..of.branches | 0.784546049993071 | 0 |
| Maximum span across hull | X..of.end.point.voxels | 0.787306840510699 | 0 |
| Maximum span across hull | X..of.junction.voxels | 0.755035732603252 | 0 |
| Maximum span across hull | X..of.junctions | 0.773617158466239 | 0 |
| Maximum span across hull | X..of.quadruple.points | 0.389184927696926 | 0 |
| Maximum span across hull | X..of.slab.voxels | 0.889998470086133 | 0 |
| Maximum span across hull | X..of.triple.points | 0.770680152346776 | 0 |
| Mean radius | Area | 0.967025005525981 | 0 |
| Mean radius | Average.branch.length | 0.016598660378233 | 0.000549534287713982 |
| Mean radius | Circularity | -0.092033317667078 | 0 |
| Mean radius | Density.of.foreground.pixels.in.hull.area | -0.606322001674621 | 0 |
| Mean radius | Diameter.of.bounding.circle | 0.98571657143719 | 0 |
| Mean radius | Foreground.pixels | 0.891688139195409 | 0 |
| Mean radius | Height.of.bounding.rectangle | 0.863868831867782 | 0 |
| Mean radius | Max.min.radii.from.circle.s.center.of.mass | 0.0238980474204883 | 6.51581973709625e-07 |
| Mean radius | Max.min.radii.from.hull.s.center.of.mass | 0.0233247293327423 | 1.19856057523293e-06 |
| Mean radius | Maximum.branch.length | 0.439244538759374 | 0 |
| Mean radius | Maximum.radius.from.circle.s.center.of.mass | 0.985716564888545 | 0 |
| Mean radius | Maximum.radius.from.hull.s.center.of.mass | 0.971536697835693 | 0 |
| Mean radius | Maximum.span.across.hull | 0.981664995212056 | 0 |
| Mean radius | Mean.radius | 1 | NA |
| Mean radius | Mean.radius.from.circle.s.center.of.mass | 0.997856741797448 | 0 |
| Mean radius | Perimeter | 0.992853206070773 | 0 |
| Mean radius | Relative.variation..CV..in.radii.from.circle.s.center.of.mass | 0.00765170242161239 | 0.111209163410665 |
| Mean radius | Relative.variation..CV..in.radii.from.hull.s.center.of.mass | 0.00708352045118884 | 0.140345499583357 |
| Mean radius | Span.ratio.of.hull..major.minor.axis. | 0.0758955148116463 | 0 |
| Mean radius | Width.of.bounding.rectangle | 0.859138173161064 | 0 |
| Mean radius | X..of.branches | 0.812800848879536 | 0 |
| Mean radius | X..of.end.point.voxels | 0.816080774706233 | 0 |
| Mean radius | X..of.junction.voxels | 0.782386551315737 | 0 |
| Mean radius | X..of.junctions | 0.801244894193699 | 0 |
| Mean radius | X..of.quadruple.points | 0.406453425453363 | 0 |
| Mean radius | X..of.slab.voxels | 0.917077976987415 | 0 |
| Mean radius | X..of.triple.points | 0.797746854624585 | 0 |
| Mean radius from circle's center of mass | Area | 0.964235773039085 | 0 |
| Mean radius from circle's center of mass | Average.branch.length | 0.0209241328826347 | 1.32466954356758e-05 |
| Mean radius from circle's center of mass | Circularity | -0.105973771584636 | 0 |
| Mean radius from circle's center of mass | Density.of.foreground.pixels.in.hull.area | -0.606552935664553 | 0 |
| Mean radius from circle's center of mass | Diameter.of.bounding.circle | 0.986960792662937 | 0 |
| Mean radius from circle's center of mass | Foreground.pixels | 0.888290131097613 | 0 |
| Mean radius from circle's center of mass | Height.of.bounding.rectangle | 0.864000005268545 | 0 |
| Mean radius from circle's center of mass | Max.min.radii.from.circle.s.center.of.mass | 0.0243386501354637 | 4.04090853667327e-07 |
| Mean radius from circle's center of mass | Max.min.radii.from.hull.s.center.of.mass | 0.0391096886717688 | 4.44089209850063e-16 |
| Mean radius from circle's center of mass | Maximum.branch.length | 0.443304756781015 | 0 |
| Mean radius from circle's center of mass | Maximum.radius.from.circle.s.center.of.mass | 0.986960779888528 | 0 |
| Mean radius from circle's center of mass | Maximum.radius.from.hull.s.center.of.mass | 0.97725523775402 | 0 |
| Mean radius from circle's center of mass | Maximum.span.across.hull | 0.983148221160357 | 0 |
| Mean radius from circle's center of mass | Mean.radius | 0.997856741797448 | 0 |
| Mean radius from circle's center of mass | Mean.radius.from.circle.s.center.of.mass | 1 | NA |
| Mean radius from circle's center of mass | Perimeter | 0.992278610931879 | 0 |
| Mean radius from circle's center of mass | Relative.variation..CV..in.radii.from.circle.s.center.of.mass | 0.00443677982103617 | 0.355719061006092 |
| Mean radius from circle's center of mass | Relative.variation..CV..in.radii.from.hull.s.center.of.mass | 0.0275160672619858 | 1.01168948773989e-08 |
| Mean radius from circle's center of mass | Span.ratio.of.hull..major.minor.axis. | 0.0844311484442145 | 0 |
| Mean radius from circle's center of mass | Width.of.bounding.rectangle | 0.857626283400406 | 0 |
| Mean radius from circle's center of mass | X..of.branches | 0.807692778171319 | 0 |
| Mean radius from circle's center of mass | X..of.end.point.voxels | 0.811013639493219 | 0 |
| Mean radius from circle's center of mass | X..of.junction.voxels | 0.777274940710946 | 0 |
| Mean radius from circle's center of mass | X..of.junctions | 0.796223772201085 | 0 |
| Mean radius from circle's center of mass | X..of.quadruple.points | 0.403505131682858 | 0 |
| Mean radius from circle's center of mass | X..of.slab.voxels | 0.913046330745086 | 0 |
| Mean radius from circle's center of mass | X..of.triple.points | 0.792807938161369 | 0 |
| Perimeter | Area | 0.986255958307987 | 0 |
| Perimeter | Average.branch.length | 0.00646466864283797 | 0.178404891468142 |
| Perimeter | Circularity | -0.0244818351411776 | 3.45372162202651e-07 |
| Perimeter | Density.of.foreground.pixels.in.hull.area | -0.600104148412333 | 0 |
| Perimeter | Diameter.of.bounding.circle | 0.981649841055076 | 0 |
| Perimeter | Foreground.pixels | 0.916936349817696 | 0 |
| Perimeter | Height.of.bounding.rectangle | 0.872397446073664 | 0 |
| Perimeter | Max.min.radii.from.circle.s.center.of.mass | 0.0085269977417882 | 0.0758995581391022 |
| Perimeter | Max.min.radii.from.hull.s.center.of.mass | -0.00481989831197671 | 0.315714548622203 |
| Perimeter | Maximum.branch.length | 0.434951574921928 | 0 |
| Perimeter | Maximum.radius.from.circle.s.center.of.mass | 0.98164983493365 | 0 |
| Perimeter | Maximum.radius.from.hull.s.center.of.mass | 0.96815550941462 | 0 |
| Perimeter | Maximum.span.across.hull | 0.976881218755601 | 0 |
| Perimeter | Mean.radius | 0.992853206070773 | 0 |
| Perimeter | Mean.radius.from.circle.s.center.of.mass | 0.992278610931879 | 0 |
| Perimeter | Perimeter | 1 | NA |
| Perimeter | Relative.variation..CV..in.radii.from.circle.s.center.of.mass | -7.73455820865891e-05 | 0.987154569874219 |
| Perimeter | Relative.variation..CV..in.radii.from.hull.s.center.of.mass | -0.0117529351979539 | 0.0144232857782876 |
| Perimeter | Span.ratio.of.hull..major.minor.axis. | 0.0152438424961856 | 0.00150715213738928 |
| Perimeter | Width.of.bounding.rectangle | 0.865327620336871 | 0 |
| Perimeter | X..of.branches | 0.839723536731482 | 0 |
| Perimeter | X..of.end.point.voxels | 0.840969897283505 | 0 |
| Perimeter | X..of.junction.voxels | 0.809014323783874 | 0 |
| Perimeter | X..of.junctions | 0.828100387362316 | 0 |
| Perimeter | X..of.quadruple.points | 0.422963686030874 | 0 |
| Perimeter | X..of.slab.voxels | 0.943107383631784 | 0 |
| Perimeter | X..of.triple.points | 0.824090258998828 | 0 |
| Relative variation (CV) in radii from circle's center of mass | Area | -0.0799183605024835 | 0 |
| Relative variation (CV) in radii from circle's center of mass | Average.branch.length | 0.0653539291382807 | 0 |
| Relative variation (CV) in radii from circle's center of mass | Circularity | -0.539787135482064 | 0 |
| Relative variation (CV) in radii from circle's center of mass | Density.of.foreground.pixels.in.hull.area | 0.0906240595631344 | 0 |
| Relative variation (CV) in radii from circle's center of mass | Diameter.of.bounding.circle | 0.134220143801569 | 0 |
| Relative variation (CV) in radii from circle's center of mass | Foreground.pixels | -0.057021514894003 | 0 |
| Relative variation (CV) in radii from circle's center of mass | Height.of.bounding.rectangle | 0.00631380963432988 | 0.188752248208777 |
| Relative variation (CV) in radii from circle's center of mass | Max.min.radii.from.circle.s.center.of.mass | 0.914378095282022 | 0 |
| Relative variation (CV) in radii from circle's center of mass | Max.min.radii.from.hull.s.center.of.mass | 0.708516981580342 | 0 |
| Relative variation (CV) in radii from circle's center of mass | Maximum.branch.length | 0.0457970486577601 | 0 |
| Relative variation (CV) in radii from circle's center of mass | Maximum.radius.from.circle.s.center.of.mass | 0.134220237298356 | 0 |
| Relative variation (CV) in radii from circle's center of mass | Maximum.radius.from.hull.s.center.of.mass | 0.121421066866228 | 0 |
| Relative variation (CV) in radii from circle's center of mass | Maximum.span.across.hull | 0.148684860425952 | 0 |
| Relative variation (CV) in radii from circle's center of mass | Mean.radius | 0.00765170242161239 | 0.111209163410665 |
| Relative variation (CV) in radii from circle's center of mass | Mean.radius.from.circle.s.center.of.mass | 0.00443677982103617 | 0.355719061006092 |
| Relative variation (CV) in radii from circle's center of mass | Perimeter | -7.73455820865891e-05 | 0.987154569874219 |
| Relative variation (CV) in radii from circle's center of mass | Relative.variation..CV..in.radii.from.circle.s.center.of.mass | 1 | NA |
| Relative variation (CV) in radii from circle's center of mass | Relative.variation..CV..in.radii.from.hull.s.center.of.mass | 0.704436488210371 | 0 |
| Relative variation (CV) in radii from circle's center of mass | Span.ratio.of.hull..major.minor.axis. | 0.634925215668862 | 0 |
| Relative variation (CV) in radii from circle's center of mass | Width.of.bounding.rectangle | -0.0471317542311732 | 0 |
| Relative variation (CV) in radii from circle's center of mass | X..of.branches | -0.0812344759568278 | 0 |
| Relative variation (CV) in radii from circle's center of mass | X..of.end.point.voxels | -0.0801277458070065 | 0 |
| Relative variation (CV) in radii from circle's center of mass | X..of.junction.voxels | -0.0778151373035474 | 0 |
| Relative variation (CV) in radii from circle's center of mass | X..of.junctions | -0.0799299892184914 | 0 |
| Relative variation (CV) in radii from circle's center of mass | X..of.quadruple.points | -0.0535952699101407 | 0 |
| Relative variation (CV) in radii from circle's center of mass | X..of.slab.voxels | -0.066821506822694 | 0 |
| Relative variation (CV) in radii from circle's center of mass | X..of.triple.points | -0.0780817141275291 | 0 |
| Relative variation (CV) in radii from hull's center of mass | Area | -0.114062615445787 | 0 |
| Relative variation (CV) in radii from hull's center of mass | Average.branch.length | 0.145774984330698 | 0 |
| Relative variation (CV) in radii from hull's center of mass | Circularity | -0.698244208304774 | 0 |
| Relative variation (CV) in radii from hull's center of mass | Density.of.foreground.pixels.in.hull.area | 0.0246487033532335 | 2.87305720547693e-07 |
| Relative variation (CV) in radii from hull's center of mass | Diameter.of.bounding.circle | 0.12536980701226 | 0 |
| Relative variation (CV) in radii from hull's center of mass | Foreground.pixels | -0.125568302914974 | 0 |
| Relative variation (CV) in radii from hull's center of mass | Height.of.bounding.rectangle | -0.00954747280364566 | 0.0468756146818299 |
| Relative variation (CV) in radii from hull's center of mass | Max.min.radii.from.circle.s.center.of.mass | 0.643588503871307 | 0 |
| Relative variation (CV) in radii from hull's center of mass | Max.min.radii.from.hull.s.center.of.mass | 0.897522857693994 | 0 |
| Relative variation (CV) in radii from hull's center of mass | Maximum.branch.length | 0.128476746535101 | 0 |
| Relative variation (CV) in radii from hull's center of mass | Maximum.radius.from.circle.s.center.of.mass | 0.125369835859444 | 0 |
| Relative variation (CV) in radii from hull's center of mass | Maximum.radius.from.hull.s.center.of.mass | 0.1863981490794 | 0 |
| Relative variation (CV) in radii from hull's center of mass | Maximum.span.across.hull | 0.137833805283324 | 0 |
| Relative variation (CV) in radii from hull's center of mass | Mean.radius | 0.00708352045118884 | 0.140345499583357 |
| Relative variation (CV) in radii from hull's center of mass | Mean.radius.from.circle.s.center.of.mass | 0.0275160672619858 | 1.01168948773989e-08 |
| Relative variation (CV) in radii from hull's center of mass | Perimeter | -0.0117529351979539 | 0.0144232857782876 |
| Relative variation (CV) in radii from hull's center of mass | Relative.variation..CV..in.radii.from.circle.s.center.of.mass | 0.704436488210371 | 0 |
| Relative variation (CV) in radii from hull's center of mass | Relative.variation..CV..in.radii.from.hull.s.center.of.mass | 1 | NA |
| Relative variation (CV) in radii from hull's center of mass | Span.ratio.of.hull..major.minor.axis. | 0.688816087902492 | 0 |
| Relative variation (CV) in radii from hull's center of mass | Width.of.bounding.rectangle | -0.0530355090570999 | 0 |
| Relative variation (CV) in radii from hull's center of mass | X..of.branches | -0.174815258890409 | 0 |
| Relative variation (CV) in radii from hull's center of mass | X..of.end.point.voxels | -0.167003183078594 | 0 |
| Relative variation (CV) in radii from hull's center of mass | X..of.junction.voxels | -0.172116100222339 | 0 |
| Relative variation (CV) in radii from hull's center of mass | X..of.junctions | -0.173127404669658 | 0 |
| Relative variation (CV) in radii from hull's center of mass | X..of.quadruple.points | -0.112687497958581 | 0 |
| Relative variation (CV) in radii from hull's center of mass | X..of.slab.voxels | -0.141286099890714 | 0 |
| Relative variation (CV) in radii from hull's center of mass | X..of.triple.points | -0.169260998705826 | 0 |
| Span ratio of hull (major/minor axis) | Area | -0.105196557602272 | 0 |
| Span ratio of hull (major/minor axis) | Average.branch.length | 0.0994586066627968 | 0 |
| Span ratio of hull (major/minor axis) | Circularity | -0.816411507650905 | 0 |
| Span ratio of hull (major/minor axis) | Density.of.foreground.pixels.in.hull.area | 0.0101756065285584 | 0.0341596101606025 |
| Span ratio of hull (major/minor axis) | Diameter.of.bounding.circle | 0.17362240010263 | 0 |
| Span ratio of hull (major/minor axis) | Foreground.pixels | -0.119435579325684 | 0 |
| Span ratio of hull (major/minor axis) | Height.of.bounding.rectangle | 0.0131968385085662 | 0.0060117630511316 |
| Span ratio of hull (major/minor axis) | Max.min.radii.from.circle.s.center.of.mass | 0.60678956151938 | 0 |
| Span ratio of hull (major/minor axis) | Max.min.radii.from.hull.s.center.of.mass | 0.734107027240098 | 0 |
| Span ratio of hull (major/minor axis) | Maximum.branch.length | 0.0833718748853752 | 0 |
| Span ratio of hull (major/minor axis) | Maximum.radius.from.circle.s.center.of.mass | 0.173622408478345 | 0 |
| Span ratio of hull (major/minor axis) | Maximum.radius.from.hull.s.center.of.mass | 0.172568796630723 | 0 |
| Span ratio of hull (major/minor axis) | Maximum.span.across.hull | 0.1943241486818 | 0 |
| Span ratio of hull (major/minor axis) | Mean.radius | 0.0758955148116463 | 0 |
| Span ratio of hull (major/minor axis) | Mean.radius.from.circle.s.center.of.mass | 0.0844311484442145 | 0 |
| Span ratio of hull (major/minor axis) | Perimeter | 0.0152438424961856 | 0.00150715213738928 |
| Span ratio of hull (major/minor axis) | Relative.variation..CV..in.radii.from.circle.s.center.of.mass | 0.634925215668862 | 0 |
| Span ratio of hull (major/minor axis) | Relative.variation..CV..in.radii.from.hull.s.center.of.mass | 0.688816087902492 | 0 |
| Span ratio of hull (major/minor axis) | Span.ratio.of.hull..major.minor.axis. | 1 | NA |
| Span ratio of hull (major/minor axis) | Width.of.bounding.rectangle | -0.0414179387946285 | 0 |
| Span ratio of hull (major/minor axis) | X..of.branches | -0.154483825251242 | 0 |
| Span ratio of hull (major/minor axis) | X..of.end.point.voxels | -0.144815177738081 | 0 |
| Span ratio of hull (major/minor axis) | X..of.junction.voxels | -0.153760419348038 | 0 |
| Span ratio of hull (major/minor axis) | X..of.junctions | -0.153007344962941 | 0 |
| Span ratio of hull (major/minor axis) | X..of.quadruple.points | -0.107702905736618 | 0 |
| Span ratio of hull (major/minor axis) | X..of.slab.voxels | -0.135348085352828 | 0 |
| Span ratio of hull (major/minor axis) | X..of.triple.points | -0.147974181544751 | 0 |
| Width of bounding rectangle | Area | 0.862730790795378 | 0 |
| Width of bounding rectangle | Average.branch.length | 0.00250623097234891 | 0.601885266995704 |
| Width of bounding rectangle | Circularity | 0.0254549907074434 | 1.16118255455433e-07 |
| Width of bounding rectangle | Density.of.foreground.pixels.in.hull.area | -0.5265647743259 | 0 |
| Width of bounding rectangle | Diameter.of.bounding.circle | 0.839699209103542 | 0 |
| Width of bounding rectangle | Foreground.pixels | 0.803645529629612 | 0 |
| Width of bounding rectangle | Height.of.bounding.rectangle | 0.557135801190955 | 0 |
| Width of bounding rectangle | Max.min.radii.from.circle.s.center.of.mass | -0.0309920175832701 | 1.0982592613118e-10 |
| Width of bounding rectangle | Max.min.radii.from.hull.s.center.of.mass | -0.0452452213569109 | 0 |
| Width of bounding rectangle | Maximum.branch.length | 0.376499280238273 | 0 |
| Width of bounding rectangle | Maximum.radius.from.circle.s.center.of.mass | 0.839699241723877 | 0 |
| Width of bounding rectangle | Maximum.radius.from.hull.s.center.of.mass | 0.829495443755098 | 0 |
| Width of bounding rectangle | Maximum.span.across.hull | 0.834505569632486 | 0 |
| Width of bounding rectangle | Mean.radius | 0.859138173161064 | 0 |
| Width of bounding rectangle | Mean.radius.from.circle.s.center.of.mass | 0.857626283400406 | 0 |
| Width of bounding rectangle | Perimeter | 0.865327620336871 | 0 |
| Width of bounding rectangle | Relative.variation..CV..in.radii.from.circle.s.center.of.mass | -0.0471317542311732 | 0 |
| Width of bounding rectangle | Relative.variation..CV..in.radii.from.hull.s.center.of.mass | -0.0530355090570999 | 0 |
| Width of bounding rectangle | Span.ratio.of.hull..major.minor.axis. | -0.0414179387946285 | 0 |
| Width of bounding rectangle | Width.of.bounding.rectangle | 1 | NA |
| Width of bounding rectangle | X..of.branches | 0.738863465186592 | 0 |
| Width of bounding rectangle | X..of.end.point.voxels | 0.739228165292799 | 0 |
| Width of bounding rectangle | X..of.junction.voxels | 0.712186672842358 | 0 |
| Width of bounding rectangle | X..of.junctions | 0.728675143292752 | 0 |
| Width of bounding rectangle | X..of.quadruple.points | 0.373939945314504 | 0 |
| Width of bounding rectangle | X..of.slab.voxels | 0.826295305004462 | 0 |
| Width of bounding rectangle | X..of.triple.points | 0.724797172204361 | 0 |
