# Supplementary material for: Development of a High-Throughput Pipeline to Characterize Microglia Morphological States at a Single-Cell Resolution
Source: eNeuro. 2024 Jul 26;11(7):ENEURO.0014-24.2024. doi: 10.1523/ENEURO.0014-24.2024 (PMC11289588; doi:10.1523/ENEURO.0014-24.2024)
Supplement: Table 3-2 — Spearman’s correlation of morphology measures and p-values for correlations, relate to Fig. 3-1A. Download Table 3-2, DOC file. [file eneuro-11-ENEURO.0014-24.2024-s004.doc]

| **measure_a** | **measure_b** | **correlation** | **pvalues** |
| --- | --- | --- | --- |
| # of branches | Area | 0.802838315825867 | 0 |
| # of branches | Average.branch.length | -0.541300004916695 | 0 |
| # of branches | Circularity | 0.144638535762818 | 0 |
| # of branches | Density.of.foreground.pixels.in.hull.area | -0.588748535467309 | 0 |
| # of branches | Diameter.of.bounding.circle | 0.699795761218074 | 0 |
| # of branches | Foreground.pixels | 0.705474120975976 | 0 |
| # of branches | Height.of.bounding.rectangle | 0.661592395657146 | 0 |
| # of branches | Max.min.radii.from.circle.s.center.of.mass | -0.0750417124357828 | 0 |
| # of branches | Max.min.radii.from.hull.s.center.of.mass | -0.178284241725058 | 0 |
| # of branches | Maximum.branch.length | 0.0165431765965601 | 0.0407777709127846 |
| # of branches | Maximum.radius.from.circle.s.center.of.mass | 0.699795888858169 | 0 |
| # of branches | Maximum.radius.from.hull.s.center.of.mass | 0.66431268206944 | 0 |
| # of branches | Maximum.span.across.hull | 0.693187194800088 | 0 |
| # of branches | Mean.radius | 0.734318223494847 | 0 |
| # of branches | Mean.radius.from.circle.s.center.of.mass | 0.726649517518614 | 0 |
| # of branches | Perimeter | 0.763636114848252 | 0 |
| # of branches | Relative.variation..CV..in.radii.from.circle.s.center.of.mass | -0.0795914446244367 | 0 |
| # of branches | Relative.variation..CV..in.radii.from.hull.s.center.of.mass | -0.190133893063063 | 0 |
| # of branches | Span.ratio.of.hull..major.minor.axis. | -0.126754352604664 | 0 |
| # of branches | Width.of.bounding.rectangle | 0.618387936993836 | 0 |
| # of branches | X..of.branches | 1 | NA |
| # of branches | X..of.end.point.voxels | 0.928448134630506 | 0 |
| # of branches | X..of.junction.voxels | 0.962707624375835 | 0 |
| # of branches | X..of.junctions | 0.99561344042755 | 0 |
| # of branches | X..of.quadruple.points | 0.451772606453885 | 0 |
| # of branches | X..of.slab.voxels | 0.917237548163551 | 0 |
| # of branches | X..of.triple.points | 0.983704794309308 | 0 |
| # of end point voxels | Area | 0.808431654292786 | 0 |
| # of end point voxels | Average.branch.length | -0.468561210978394 | 0 |
| # of end point voxels | Circularity | 0.133385849460867 | 0 |
| # of end point voxels | Density.of.foreground.pixels.in.hull.area | -0.64799816435722 | 0 |
| # of end point voxels | Diameter.of.bounding.circle | 0.707721437163649 | 0 |
| # of end point voxels | Foreground.pixels | 0.680442412593973 | 0 |
| # of end point voxels | Height.of.bounding.rectangle | 0.669618694947446 | 0 |
| # of end point voxels | Max.min.radii.from.circle.s.center.of.mass | -0.073941335485816 | 0 |
| # of end point voxels | Max.min.radii.from.hull.s.center.of.mass | -0.166899261134277 | 0 |
| # of end point voxels | Maximum.branch.length | 0.0457716990855284 | 1.48873786631754e-08 |
| # of end point voxels | Maximum.radius.from.circle.s.center.of.mass | 0.707721585393467 | 0 |
| # of end point voxels | Maximum.radius.from.hull.s.center.of.mass | 0.673493102738194 | 0 |
| # of end point voxels | Maximum.span.across.hull | 0.700951368485575 | 0 |
| # of end point voxels | Mean.radius | 0.742911771896499 | 0 |
| # of end point voxels | Mean.radius.from.circle.s.center.of.mass | 0.734619882626655 | 0 |
| # of end point voxels | Perimeter | 0.770805306557271 | 0 |
| # of end point voxels | Relative.variation..CV..in.radii.from.circle.s.center.of.mass | -0.0769498548315971 | 0 |
| # of end point voxels | Relative.variation..CV..in.radii.from.hull.s.center.of.mass | -0.178280302020069 | 0 |
| # of end point voxels | Span.ratio.of.hull..major.minor.axis. | -0.122364182325642 | 0 |
| # of end point voxels | Width.of.bounding.rectangle | 0.622279772552397 | 0 |
| # of end point voxels | X..of.branches | 0.928448134630506 | 0 |
| # of end point voxels | X..of.end.point.voxels | 1 | NA |
| # of end point voxels | X..of.junction.voxels | 0.867903130067007 | 0 |
| # of end point voxels | X..of.junctions | 0.893840718807504 | 0 |
| # of end point voxels | X..of.quadruple.points | 0.433871904615127 | 0 |
| # of end point voxels | X..of.slab.voxels | 0.87398244119356 | 0 |
| # of end point voxels | X..of.triple.points | 0.879866175815331 | 0 |
| # of junction voxels | Area | 0.760488814682213 | 0 |
| # of junction voxels | Average.branch.length | -0.523368895851906 | 0 |
| # of junction voxels | Circularity | 0.139877849843813 | 0 |
| # of junction voxels | Density.of.foreground.pixels.in.hull.area | -0.546507543684237 | 0 |
| # of junction voxels | Diameter.of.bounding.circle | 0.662417144783249 | 0 |
| # of junction voxels | Foreground.pixels | 0.675144317695844 | 0 |
| # of junction voxels | Height.of.bounding.rectangle | 0.62512704337344 | 0 |
| # of junction voxels | Max.min.radii.from.circle.s.center.of.mass | -0.0725218800800832 | 0 |
| # of junction voxels | Max.min.radii.from.hull.s.center.of.mass | -0.173626064246733 | 0 |
| # of junction voxels | Maximum.branch.length | 0.00865350214513194 | 0.284589252692996 |
| # of junction voxels | Maximum.radius.from.circle.s.center.of.mass | 0.662417208599931 | 0 |
| # of junction voxels | Maximum.radius.from.hull.s.center.of.mass | 0.629355944772793 | 0 |
| # of junction voxels | Maximum.span.across.hull | 0.656177474467146 | 0 |
| # of junction voxels | Mean.radius | 0.695023756756056 | 0 |
| # of junction voxels | Mean.radius.from.circle.s.center.of.mass | 0.687900972051311 | 0 |
| # of junction voxels | Perimeter | 0.72312969391464 | 0 |
| # of junction voxels | Relative.variation..CV..in.radii.from.circle.s.center.of.mass | -0.0768297475910305 | 0 |
| # of junction voxels | Relative.variation..CV..in.radii.from.hull.s.center.of.mass | -0.182106906902988 | 0 |
| # of junction voxels | Span.ratio.of.hull..major.minor.axis. | -0.120934710649621 | 0 |
| # of junction voxels | Width.of.bounding.rectangle | 0.587799270607362 | 0 |
| # of junction voxels | X..of.branches | 0.962707624375835 | 0 |
| # of junction voxels | X..of.end.point.voxels | 0.867903130067007 | 0 |
| # of junction voxels | X..of.junction.voxels | 1 | NA |
| # of junction voxels | X..of.junctions | 0.962247590182906 | 0 |
| # of junction voxels | X..of.quadruple.points | 0.48583957105108 | 0 |
| # of junction voxels | X..of.slab.voxels | 0.876636130730476 | 0 |
| # of junction voxels | X..of.triple.points | 0.943841240675936 | 0 |
| # of junctions | Area | 0.78730100033053 | 0 |
| # of junctions | Average.branch.length | -0.549545890550375 | 0 |
| # of junctions | Circularity | 0.142801184424732 | 0 |
| # of junctions | Density.of.foreground.pixels.in.hull.area | -0.566653832633416 | 0 |
| # of junctions | Diameter.of.bounding.circle | 0.68645334690181 | 0 |
| # of junctions | Foreground.pixels | 0.697994312913483 | 0 |
| # of junctions | Height.of.bounding.rectangle | 0.648522743683108 | 0 |
| # of junctions | Max.min.radii.from.circle.s.center.of.mass | -0.0730609472482384 | 0 |
| # of junctions | Max.min.radii.from.hull.s.center.of.mass | -0.175833124815153 | 0 |
| # of junctions | Maximum.branch.length | 0.0096203951980501 | 0.234189804870143 |
| # of junctions | Maximum.radius.from.circle.s.center.of.mass | 0.686453469992875 | 0 |
| # of junctions | Maximum.radius.from.hull.s.center.of.mass | 0.651560238230033 | 0 |
| # of junctions | Maximum.span.across.hull | 0.680048189184638 | 0 |
| # of junctions | Mean.radius | 0.720068119171879 | 0 |
| # of junctions | Mean.radius.from.circle.s.center.of.mass | 0.712711182617409 | 0 |
| # of junctions | Perimeter | 0.74890155809533 | 0 |
| # of junctions | Relative.variation..CV..in.radii.from.circle.s.center.of.mass | -0.0781523370079931 | 0 |
| # of junctions | Relative.variation..CV..in.radii.from.hull.s.center.of.mass | -0.1877640943637 | 0 |
| # of junctions | Span.ratio.of.hull..major.minor.axis. | -0.123971118900431 | 0 |
| # of junctions | Width.of.bounding.rectangle | 0.607050627414771 | 0 |
| # of junctions | X..of.branches | 0.99561344042755 | 0 |
| # of junctions | X..of.end.point.voxels | 0.893840718807504 | 0 |
| # of junctions | X..of.junction.voxels | 0.962247590182906 | 0 |
| # of junctions | X..of.junctions | 1 | NA |
| # of junctions | X..of.quadruple.points | 0.418781663238617 | 0 |
| # of junctions | X..of.slab.voxels | 0.908230218092835 | 0 |
| # of junctions | X..of.triple.points | 0.993211512590015 | 0 |
| # of quadruple points | Area | 0.339069438164287 | 0 |
| # of quadruple points | Average.branch.length | -0.20049607196676 | 0 |
| # of quadruple points | Circularity | 0.0889948765863107 | 0 |
| # of quadruple points | Density.of.foreground.pixels.in.hull.area | -0.234703206854671 | 0 |
| # of quadruple points | Diameter.of.bounding.circle | 0.28412351882983 | 0 |
| # of quadruple points | Foreground.pixels | 0.307402430913355 | 0 |
| # of quadruple points | Height.of.bounding.rectangle | 0.276143318234118 | 0 |
| # of quadruple points | Max.min.radii.from.circle.s.center.of.mass | -0.0486082485349146 | 1.80781256631235e-09 |
| # of quadruple points | Max.min.radii.from.hull.s.center.of.mass | -0.104877418657893 | 0 |
| # of quadruple points | Maximum.branch.length | 0.00957289446852036 | 0.236507644018474 |
| # of quadruple points | Maximum.radius.from.circle.s.center.of.mass | 0.284123621689345 | 0 |
| # of quadruple points | Maximum.radius.from.hull.s.center.of.mass | 0.267396954187571 | 0 |
| # of quadruple points | Maximum.span.across.hull | 0.28079068032621 | 0 |
| # of quadruple points | Mean.radius | 0.301698443281639 | 0 |
| # of quadruple points | Mean.radius.from.circle.s.center.of.mass | 0.297822595303561 | 0 |
| # of quadruple points | Perimeter | 0.316185353394684 | 0 |
| # of quadruple points | Relative.variation..CV..in.radii.from.circle.s.center.of.mass | -0.0475437913725715 | 4.04500166695243e-09 |
| # of quadruple points | Relative.variation..CV..in.radii.from.hull.s.center.of.mass | -0.109067704761233 | 0 |
| # of quadruple points | Span.ratio.of.hull..major.minor.axis. | -0.0722421443840065 | 0 |
| # of quadruple points | Width.of.bounding.rectangle | 0.259090641138724 | 0 |
| # of quadruple points | X..of.branches | 0.451772606453885 | 0 |
| # of quadruple points | X..of.end.point.voxels | 0.433871904615127 | 0 |
| # of quadruple points | X..of.junction.voxels | 0.48583957105108 | 0 |
| # of quadruple points | X..of.junctions | 0.418781663238617 | 0 |
| # of quadruple points | X..of.quadruple.points | 1 | NA |
| # of quadruple points | X..of.slab.voxels | 0.424611742511911 | 0 |
| # of quadruple points | X..of.triple.points | 0.324438814960663 | 0 |
| # of slab voxels | Area | 0.949393620746746 | 0 |
| # of slab voxels | Average.branch.length | -0.198715908318534 | 0 |
| # of slab voxels | Circularity | 0.099799759366664 | 0 |
| # of slab voxels | Density.of.foreground.pixels.in.hull.area | -0.642299850202467 | 0 |
| # of slab voxels | Diameter.of.bounding.circle | 0.856607617744586 | 0 |
| # of slab voxels | Foreground.pixels | 0.86158164430702 | 0 |
| # of slab voxels | Height.of.bounding.rectangle | 0.780951894231432 | 0 |
| # of slab voxels | Max.min.radii.from.circle.s.center.of.mass | -0.0553382340885388 | 7.48467954281296e-12 |
| # of slab voxels | Max.min.radii.from.hull.s.center.of.mass | -0.135165988108168 | 0 |
| # of slab voxels | Maximum.branch.length | 0.261737182946652 | 0 |
| # of slab voxels | Maximum.radius.from.circle.s.center.of.mass | 0.856607631216895 | 0 |
| # of slab voxels | Maximum.radius.from.hull.s.center.of.mass | 0.824098717249763 | 0 |
| # of slab voxels | Maximum.span.across.hull | 0.848886788247582 | 0 |
| # of slab voxels | Mean.radius | 0.888886866300003 | 0 |
| # of slab voxels | Mean.radius.from.circle.s.center.of.mass | 0.882671493394647 | 0 |
| # of slab voxels | Perimeter | 0.92001543488791 | 0 |
| # of slab voxels | Relative.variation..CV..in.radii.from.circle.s.center.of.mass | -0.0568526193128191 | 1.98152605435098e-12 |
| # of slab voxels | Relative.variation..CV..in.radii.from.hull.s.center.of.mass | -0.147072581510514 | 0 |
| # of slab voxels | Span.ratio.of.hull..major.minor.axis. | -0.10181994175478 | 0 |
| # of slab voxels | Width.of.bounding.rectangle | 0.753325541487284 | 0 |
| # of slab voxels | X..of.branches | 0.917237548163551 | 0 |
| # of slab voxels | X..of.end.point.voxels | 0.87398244119356 | 0 |
| # of slab voxels | X..of.junction.voxels | 0.876636130730476 | 0 |
| # of slab voxels | X..of.junctions | 0.908230218092835 | 0 |
| # of slab voxels | X..of.quadruple.points | 0.424611742511911 | 0 |
| # of slab voxels | X..of.slab.voxels | 1 | NA |
| # of slab voxels | X..of.triple.points | 0.8963432980757 | 0 |
| # of triple points | Area | 0.781841619594792 | 0 |
| # of triple points | Average.branch.length | -0.549860532013595 | 0 |
| # of triple points | Circularity | 0.138055294982599 | 0 |
| # of triple points | Density.of.foreground.pixels.in.hull.area | -0.563724360655924 | 0 |
| # of triple points | Diameter.of.bounding.circle | 0.683594705430163 | 0 |
| # of triple points | Foreground.pixels | 0.692797359359377 | 0 |
| # of triple points | Height.of.bounding.rectangle | 0.644570080378894 | 0 |
| # of triple points | Max.min.radii.from.circle.s.center.of.mass | -0.0701059351697314 | 0 |
| # of triple points | Max.min.radii.from.hull.s.center.of.mass | -0.170139772631169 | 0 |
| # of triple points | Maximum.branch.length | 0.0102052073181881 | 0.206964859245472 |
| # of triple points | Maximum.radius.from.circle.s.center.of.mass | 0.683594828068555 | 0 |
| # of triple points | Maximum.radius.from.hull.s.center.of.mass | 0.649464164724829 | 0 |
| # of triple points | Maximum.span.across.hull | 0.677341549877694 | 0 |
| # of triple points | Mean.radius | 0.716553990397988 | 0 |
| # of triple points | Mean.radius.from.circle.s.center.of.mass | 0.709378230542683 | 0 |
| # of triple points | Perimeter | 0.744735312630939 | 0 |
| # of triple points | Relative.variation..CV..in.radii.from.circle.s.center.of.mass | -0.0757751523151772 | 0 |
| # of triple points | Relative.variation..CV..in.radii.from.hull.s.center.of.mass | -0.182261489676345 | 0 |
| # of triple points | Span.ratio.of.hull..major.minor.axis. | -0.119926020441094 | 0 |
| # of triple points | Width.of.bounding.rectangle | 0.603963087727479 | 0 |
| # of triple points | X..of.branches | 0.983704794309308 | 0 |
| # of triple points | X..of.end.point.voxels | 0.879866175815331 | 0 |
| # of triple points | X..of.junction.voxels | 0.943841240675936 | 0 |
| # of triple points | X..of.junctions | 0.993211512590015 | 0 |
| # of triple points | X..of.quadruple.points | 0.324438814960663 | 0 |
| # of triple points | X..of.slab.voxels | 0.8963432980757 | 0 |
| # of triple points | X..of.triple.points | 1 | NA |
| Area | Area | 1 | NA |
| Area | Average.branch.length | -0.0325792686861346 | 5.5859650867518e-05 |
| Area | Circularity | 0.0546849002959205 | 1.31379351842043e-11 |
| Area | Density.of.foreground.pixels.in.hull.area | -0.67290992698681 | 0 |
| Area | Diameter.of.bounding.circle | 0.916874918444294 | 0 |
| Area | Foreground.pixels | 0.909648635931245 | 0 |
| Area | Height.of.bounding.rectangle | 0.82294288024086 | 0 |
| Area | Max.min.radii.from.circle.s.center.of.mass | -0.0741931089804992 | 0 |
| Area | Max.min.radii.from.hull.s.center.of.mass | -0.10558614371317 | 0 |
| Area | Maximum.branch.length | 0.376334472296143 | 0 |
| Area | Maximum.radius.from.circle.s.center.of.mass | 0.916874814041282 | 0 |
| Area | Maximum.radius.from.hull.s.center.of.mass | 0.892105690533823 | 0 |
| Area | Maximum.span.across.hull | 0.908497282079629 | 0 |
| Area | Mean.radius | 0.952420643414535 | 0 |
| Area | Mean.radius.from.circle.s.center.of.mass | 0.948722877580332 | 0 |
| Area | Perimeter | 0.978815558151304 | 0 |
| Area | Relative.variation..CV..in.radii.from.circle.s.center.of.mass | -0.0779101836195323 | 0 |
| Area | Relative.variation..CV..in.radii.from.hull.s.center.of.mass | -0.11677557245703 | 0 |
| Area | Span.ratio.of.hull..major.minor.axis. | -0.0802447334970643 | 0 |
| Area | Width.of.bounding.rectangle | 0.807279424177353 | 0 |
| Area | X..of.branches | 0.802838315825867 | 0 |
| Area | X..of.end.point.voxels | 0.808431654292786 | 0 |
| Area | X..of.junction.voxels | 0.760488814682213 | 0 |
| Area | X..of.junctions | 0.78730100033053 | 0 |
| Area | X..of.quadruple.points | 0.339069438164287 | 0 |
| Area | X..of.slab.voxels | 0.949393620746746 | 0 |
| Area | X..of.triple.points | 0.781841619594792 | 0 |
| Average branch length | Area | -0.0325792686861346 | 5.5859650867518e-05 |
| Average branch length | Average.branch.length | 1 | NA |
| Average branch length | Circularity | -0.142482652257021 | 0 |
| Average branch length | Density.of.foreground.pixels.in.hull.area | 0.155113359922814 | 0 |
| Average branch length | Diameter.of.bounding.circle | 0.0271754589177456 | 0.000776620759256996 |
| Average branch length | Foreground.pixels | 0.0449106989968263 | 2.75671294680535e-08 |
| Average branch length | Height.of.bounding.rectangle | -0.0234802792356412 | 0.00368606323742737 |
| Average branch length | Max.min.radii.from.circle.s.center.of.mass | 0.0735483702868901 | 0 |
| Average branch length | Max.min.radii.from.hull.s.center.of.mass | 0.158925276292152 | 0 |
| Average branch length | Maximum.branch.length | 0.528742866862097 | 0 |
| Average branch length | Maximum.radius.from.circle.s.center.of.mass | 0.0271751948169098 | 0.000776712640470079 |
| Average branch length | Maximum.radius.from.hull.s.center.of.mass | 0.0481442091057528 | 2.57358845345834e-09 |
| Average branch length | Maximum.span.across.hull | 0.0280627452134027 | 0.000518957588333535 |
| Average branch length | Mean.radius | 0.00678113912890915 | 0.401733229240904 |
| Average branch length | Mean.radius.from.circle.s.center.of.mass | 0.0135702283388304 | 0.0933274601798371 |
| Average branch length | Perimeter | 0.000113153617280275 | 0.988836376582857 |
| Average branch length | Relative.variation..CV..in.radii.from.circle.s.center.of.mass | 0.0817154463979024 | 0 |
| Average branch length | Relative.variation..CV..in.radii.from.hull.s.center.of.mass | 0.166462517943933 | 0 |
| Average branch length | Span.ratio.of.hull..major.minor.axis. | 0.102182140868491 | 0 |
| Average branch length | Width.of.bounding.rectangle | 0.0186191609364788 | 0.0213043169789211 |
| Average branch length | X..of.branches | -0.541300004916695 | 0 |
| Average branch length | X..of.end.point.voxels | -0.468561210978394 | 0 |
| Average branch length | X..of.junction.voxels | -0.523368895851906 | 0 |
| Average branch length | X..of.junctions | -0.549545890550375 | 0 |
| Average branch length | X..of.quadruple.points | -0.20049607196676 | 0 |
| Average branch length | X..of.slab.voxels | -0.198715908318534 | 0 |
| Average branch length | X..of.triple.points | -0.549860532013595 | 0 |
| Circularity | Area | 0.0546849002959205 | 1.31379351842043e-11 |
| Circularity | Average.branch.length | -0.142482652257021 | 0 |
| Circularity | Circularity | 1 | NA |
| Circularity | Density.of.foreground.pixels.in.hull.area | 0.0716581141237648 | 0 |
| Circularity | Diameter.of.bounding.circle | -0.289545104554997 | 0 |
| Circularity | Foreground.pixels | 0.108721757655263 | 0 |
| Circularity | Height.of.bounding.rectangle | -0.0595082601861066 | 1.77191594730175e-13 |
| Circularity | Max.min.radii.from.circle.s.center.of.mass | -0.547967836888909 | 0 |
| Circularity | Max.min.radii.from.hull.s.center.of.mass | -0.717623498345842 | 0 |
| Circularity | Maximum.branch.length | -0.176828487074485 | 0 |
| Circularity | Maximum.radius.from.circle.s.center.of.mass | -0.289545252982585 | 0 |
| Circularity | Maximum.radius.from.hull.s.center.of.mass | -0.310504818970604 | 0 |
| Circularity | Maximum.span.across.hull | -0.297703467082943 | 0 |
| Circularity | Mean.radius | -0.192322144772804 | 0 |
| Circularity | Mean.radius.from.circle.s.center.of.mass | -0.21007531574719 | 0 |
| Circularity | Perimeter | -0.119019524586443 | 0 |
| Circularity | Relative.variation..CV..in.radii.from.circle.s.center.of.mass | -0.545803098154305 | 0 |
| Circularity | Relative.variation..CV..in.radii.from.hull.s.center.of.mass | -0.678997159078028 | 0 |
| Circularity | Span.ratio.of.hull..major.minor.axis. | -0.810826657960008 | 0 |
| Circularity | Width.of.bounding.rectangle | -0.106510868227047 | 0 |
| Circularity | X..of.branches | 0.144638535762818 | 0 |
| Circularity | X..of.end.point.voxels | 0.133385849460867 | 0 |
| Circularity | X..of.junction.voxels | 0.139877849843813 | 0 |
| Circularity | X..of.junctions | 0.142801184424732 | 0 |
| Circularity | X..of.quadruple.points | 0.0889948765863107 | 0 |
| Circularity | X..of.slab.voxels | 0.099799759366664 | 0 |
| Circularity | X..of.triple.points | 0.138055294982599 | 0 |
| Density of foreground pixels in hull area | Area | -0.67290992698681 | 0 |
| Density of foreground pixels in hull area | Average.branch.length | 0.155113359922814 | 0 |
| Density of foreground pixels in hull area | Circularity | 0.0716581141237648 | 0 |
| Density of foreground pixels in hull area | Density.of.foreground.pixels.in.hull.area | 1 | NA |
| Density of foreground pixels in hull area | Diameter.of.bounding.circle | -0.641172321601682 | 0 |
| Density of foreground pixels in hull area | Foreground.pixels | -0.324389885530837 | 0 |
| Density of foreground pixels in hull area | Height.of.bounding.rectangle | -0.584041391003146 | 0 |
| Density of foreground pixels in hull area | Max.min.radii.from.circle.s.center.of.mass | 0.089194721694009 | 0 |
| Density of foreground pixels in hull area | Max.min.radii.from.hull.s.center.of.mass | 0.0391494815513112 | 1.27972973507084e-06 |
| Density of foreground pixels in hull area | Maximum.branch.length | -0.254264893886761 | 0 |
| Density of foreground pixels in hull area | Maximum.radius.from.circle.s.center.of.mass | -0.641172288864286 | 0 |
| Density of foreground pixels in hull area | Maximum.radius.from.hull.s.center.of.mass | -0.635620954417605 | 0 |
| Density of foreground pixels in hull area | Maximum.span.across.hull | -0.634501429491596 | 0 |
| Density of foreground pixels in hull area | Mean.radius | -0.678325274391104 | 0 |
| Density of foreground pixels in hull area | Mean.radius.from.circle.s.center.of.mass | -0.673994419553791 | 0 |
| Density of foreground pixels in hull area | Perimeter | -0.675527881586245 | 0 |
| Density of foreground pixels in hull area | Relative.variation..CV..in.radii.from.circle.s.center.of.mass | 0.0859501476204992 | 0 |
| Density of foreground pixels in hull area | Relative.variation..CV..in.radii.from.hull.s.center.of.mass | 0.0435792410880904 | 6.99334978815358e-08 |
| Density of foreground pixels in hull area | Span.ratio.of.hull..major.minor.axis. | 0.0304276325112855 | 0.00016762349709043 |
| Density of foreground pixels in hull area | Width.of.bounding.rectangle | -0.554196624245954 | 0 |
| Density of foreground pixels in hull area | X..of.branches | -0.588748535467309 | 0 |
| Density of foreground pixels in hull area | X..of.end.point.voxels | -0.64799816435722 | 0 |
| Density of foreground pixels in hull area | X..of.junction.voxels | -0.546507543684237 | 0 |
| Density of foreground pixels in hull area | X..of.junctions | -0.566653832633416 | 0 |
| Density of foreground pixels in hull area | X..of.quadruple.points | -0.234703206854671 | 0 |
| Density of foreground pixels in hull area | X..of.slab.voxels | -0.642299850202467 | 0 |
| Density of foreground pixels in hull area | X..of.triple.points | -0.563724360655924 | 0 |
| Diameter of bounding circle | Area | 0.916874918444294 | 0 |
| Diameter of bounding circle | Average.branch.length | 0.0271754589177456 | 0.000776620759256996 |
| Diameter of bounding circle | Circularity | -0.289545104554997 | 0 |
| Diameter of bounding circle | Density.of.foreground.pixels.in.hull.area | -0.641172321601682 | 0 |
| Diameter of bounding circle | Diameter.of.bounding.circle | 1 | NA |
| Diameter of bounding circle | Foreground.pixels | 0.821861890013473 | 0 |
| Diameter of bounding circle | Height.of.bounding.rectangle | 0.796310587331483 | 0 |
| Diameter of bounding circle | Max.min.radii.from.circle.s.center.of.mass | 0.169415871576688 | 0 |
| Diameter of bounding circle | Max.min.radii.from.hull.s.center.of.mass | 0.180931754130514 | 0 |
| Diameter of bounding circle | Maximum.branch.length | 0.42522600299919 | 0 |
| Diameter of bounding circle | Maximum.radius.from.circle.s.center.of.mass | 0.999999999850699 | 0 |
| Diameter of bounding circle | Maximum.radius.from.hull.s.center.of.mass | 0.977501711565591 | 0 |
| Diameter of bounding circle | Maximum.span.across.hull | 0.998554630794815 | 0 |
| Diameter of bounding circle | Mean.radius | 0.979034848836797 | 0 |
| Diameter of bounding circle | Mean.radius.from.circle.s.center.of.mass | 0.981397653430393 | 0 |
| Diameter of bounding circle | Perimeter | 0.974699116654495 | 0 |
| Diameter of bounding circle | Relative.variation..CV..in.radii.from.circle.s.center.of.mass | 0.181365039425767 | 0 |
| Diameter of bounding circle | Relative.variation..CV..in.radii.from.hull.s.center.of.mass | 0.15994972896523 | 0 |
| Diameter of bounding circle | Span.ratio.of.hull..major.minor.axis. | 0.256888512538767 | 0 |
| Diameter of bounding circle | Width.of.bounding.rectangle | 0.808037533311594 | 0 |
| Diameter of bounding circle | X..of.branches | 0.699795761218074 | 0 |
| Diameter of bounding circle | X..of.end.point.voxels | 0.707721437163649 | 0 |
| Diameter of bounding circle | X..of.junction.voxels | 0.662417144783249 | 0 |
| Diameter of bounding circle | X..of.junctions | 0.68645334690181 | 0 |
| Diameter of bounding circle | X..of.quadruple.points | 0.28412351882983 | 0 |
| Diameter of bounding circle | X..of.slab.voxels | 0.856607617744586 | 0 |
| Diameter of bounding circle | X..of.triple.points | 0.683594705430163 | 0 |
| Foreground pixels | Area | 0.909648635931245 | 0 |
| Foreground pixels | Average.branch.length | 0.0449106989968263 | 2.75671294680535e-08 |
| Foreground pixels | Circularity | 0.108721757655263 | 0 |
| Foreground pixels | Density.of.foreground.pixels.in.hull.area | -0.324389885530837 | 0 |
| Foreground pixels | Diameter.of.bounding.circle | 0.821861890013473 | 0 |
| Foreground pixels | Foreground.pixels | 1 | NA |
| Foreground pixels | Height.of.bounding.rectangle | 0.734341141934853 | 0 |
| Foreground pixels | Max.min.radii.from.circle.s.center.of.mass | -0.0445403228265849 | 3.58105611830695e-08 |
| Foreground pixels | Max.min.radii.from.hull.s.center.of.mass | -0.113439898597279 | 0 |
| Foreground pixels | Maximum.branch.length | 0.346314657462417 | 0 |
| Foreground pixels | Maximum.radius.from.circle.s.center.of.mass | 0.821861793241616 | 0 |
| Foreground pixels | Maximum.radius.from.hull.s.center.of.mass | 0.792855537272186 | 0 |
| Foreground pixels | Maximum.span.across.hull | 0.815113483873455 | 0 |
| Foreground pixels | Mean.radius | 0.84542190422765 | 0 |
| Foreground pixels | Mean.radius.from.circle.s.center.of.mass | 0.843214317065048 | 0 |
| Foreground pixels | Perimeter | 0.88079282107545 | 0 |
| Foreground pixels | Relative.variation..CV..in.radii.from.circle.s.center.of.mass | -0.0514627884621109 | 1.91710869401618e-10 |
| Foreground pixels | Relative.variation..CV..in.radii.from.hull.s.center.of.mass | -0.12643948779324 | 0 |
| Foreground pixels | Span.ratio.of.hull..major.minor.axis. | -0.0832079357437125 | 0 |
| Foreground pixels | Width.of.bounding.rectangle | 0.731479752471551 | 0 |
| Foreground pixels | X..of.branches | 0.705474120975976 | 0 |
| Foreground pixels | X..of.end.point.voxels | 0.680442412593973 | 0 |
| Foreground pixels | X..of.junction.voxels | 0.675144317695844 | 0 |
| Foreground pixels | X..of.junctions | 0.697994312913483 | 0 |
| Foreground pixels | X..of.quadruple.points | 0.307402430913355 | 0 |
| Foreground pixels | X..of.slab.voxels | 0.86158164430702 | 0 |
| Foreground pixels | X..of.triple.points | 0.692797359359377 | 0 |
| Height of bounding rectangle | Area | 0.82294288024086 | 0 |
| Height of bounding rectangle | Average.branch.length | -0.0234802792356412 | 0.00368606323742737 |
| Height of bounding rectangle | Circularity | -0.0595082601861066 | 1.77191594730175e-13 |
| Height of bounding rectangle | Density.of.foreground.pixels.in.hull.area | -0.584041391003146 | 0 |
| Height of bounding rectangle | Diameter.of.bounding.circle | 0.796310587331483 | 0 |
| Height of bounding rectangle | Foreground.pixels | 0.734341141934853 | 0 |
| Height of bounding rectangle | Height.of.bounding.rectangle | 1 | NA |
| Height of bounding rectangle | Max.min.radii.from.circle.s.center.of.mass | -0.0122078584495928 | 0.131141418086985 |
| Height of bounding rectangle | Max.min.radii.from.hull.s.center.of.mass | -0.0188783876271763 | 0.0195637749300235 |
| Height of bounding rectangle | Maximum.branch.length | 0.323004796840563 | 0 |
| Height of bounding rectangle | Maximum.radius.from.circle.s.center.of.mass | 0.796310444680658 | 0 |
| Height of bounding rectangle | Maximum.radius.from.hull.s.center.of.mass | 0.778002690552097 | 0 |
| Height of bounding rectangle | Maximum.span.across.hull | 0.790068041691915 | 0 |
| Height of bounding rectangle | Mean.radius | 0.812311678836164 | 0 |
| Height of bounding rectangle | Mean.radius.from.circle.s.center.of.mass | 0.812325406156178 | 0 |
| Height of bounding rectangle | Perimeter | 0.82714544485697 | 0 |
| Height of bounding rectangle | Relative.variation..CV..in.radii.from.circle.s.center.of.mass | -0.0141890273719586 | 0.0793227682495066 |
| Height of bounding rectangle | Relative.variation..CV..in.radii.from.hull.s.center.of.mass | -0.0330367843930348 | 4.38361694825584e-05 |
| Height of bounding rectangle | Span.ratio.of.hull..major.minor.axis. | 0.0166481829014438 | 0.0395163008648667 |
| Height of bounding rectangle | Width.of.bounding.rectangle | 0.425217807875554 | 0 |
| Height of bounding rectangle | X..of.branches | 0.661592395657146 | 0 |
| Height of bounding rectangle | X..of.end.point.voxels | 0.669618694947446 | 0 |
| Height of bounding rectangle | X..of.junction.voxels | 0.62512704337344 | 0 |
| Height of bounding rectangle | X..of.junctions | 0.648522743683108 | 0 |
| Height of bounding rectangle | X..of.quadruple.points | 0.276143318234118 | 0 |
| Height of bounding rectangle | X..of.slab.voxels | 0.780951894231432 | 0 |
| Height of bounding rectangle | X..of.triple.points | 0.644570080378894 | 0 |
| Max/min radii from circle's center of mass | Area | -0.0741931089804992 | 0 |
| Max/min radii from circle's center of mass | Average.branch.length | 0.0735483702868901 | 0 |
| Max/min radii from circle's center of mass | Circularity | -0.547967836888909 | 0 |
| Max/min radii from circle's center of mass | Density.of.foreground.pixels.in.hull.area | 0.089194721694009 | 0 |
| Max/min radii from circle's center of mass | Diameter.of.bounding.circle | 0.169415871576688 | 0 |
| Max/min radii from circle's center of mass | Foreground.pixels | -0.0445403228265849 | 3.58105611830695e-08 |
| Max/min radii from circle's center of mass | Height.of.bounding.rectangle | -0.0122078584495928 | 0.131141418086985 |
| Max/min radii from circle's center of mass | Max.min.radii.from.circle.s.center.of.mass | 1 | NA |
| Max/min radii from circle's center of mass | Max.min.radii.from.hull.s.center.of.mass | 0.68542291166334 | 0 |
| Max/min radii from circle's center of mass | Maximum.branch.length | 0.0629301578497894 | 6.66133814775094e-15 |
| Max/min radii from circle's center of mass | Maximum.radius.from.circle.s.center.of.mass | 0.169416033466413 | 0 |
| Max/min radii from circle's center of mass | Maximum.radius.from.hull.s.center.of.mass | 0.158125247696923 | 0 |
| Max/min radii from circle's center of mass | Maximum.span.across.hull | 0.184908722692119 | 0 |
| Max/min radii from circle's center of mass | Mean.radius | 0.0435028049299023 | 7.37132617167902e-08 |
| Max/min radii from circle's center of mass | Mean.radius.from.circle.s.center.of.mass | 0.0425270268080409 | 1.43235591387736e-07 |
| Max/min radii from circle's center of mass | Perimeter | 0.0263691968166292 | 0.00110924633183385 |
| Max/min radii from circle's center of mass | Relative.variation..CV..in.radii.from.circle.s.center.of.mass | 0.915805546678529 | 0 |
| Max/min radii from circle's center of mass | Relative.variation..CV..in.radii.from.hull.s.center.of.mass | 0.60442572878505 | 0 |
| Max/min radii from circle's center of mass | Span.ratio.of.hull..major.minor.axis. | 0.618403626910029 | 0 |
| Max/min radii from circle's center of mass | Width.of.bounding.rectangle | 0.023487831316039 | 0.00367507951661405 |
| Max/min radii from circle's center of mass | X..of.branches | -0.0750417124357828 | 0 |
| Max/min radii from circle's center of mass | X..of.end.point.voxels | -0.073941335485816 | 0 |
| Max/min radii from circle's center of mass | X..of.junction.voxels | -0.0725218800800832 | 0 |
| Max/min radii from circle's center of mass | X..of.junctions | -0.0730609472482384 | 0 |
| Max/min radii from circle's center of mass | X..of.quadruple.points | -0.0486082485349146 | 1.80781256631235e-09 |
| Max/min radii from circle's center of mass | X..of.slab.voxels | -0.0553382340885388 | 7.48467954281296e-12 |
| Max/min radii from circle's center of mass | X..of.triple.points | -0.0701059351697314 | 0 |
| Max/min radii from hull's center of mass | Area | -0.10558614371317 | 0 |
| Max/min radii from hull's center of mass | Average.branch.length | 0.158925276292152 | 0 |
| Max/min radii from hull's center of mass | Circularity | -0.717623498345842 | 0 |
| Max/min radii from hull's center of mass | Density.of.foreground.pixels.in.hull.area | 0.0391494815513112 | 1.27972973507084e-06 |
| Max/min radii from hull's center of mass | Diameter.of.bounding.circle | 0.180931754130514 | 0 |
| Max/min radii from hull's center of mass | Foreground.pixels | -0.113439898597279 | 0 |
| Max/min radii from hull's center of mass | Height.of.bounding.rectangle | -0.0188783876271763 | 0.0195637749300235 |
| Max/min radii from hull's center of mass | Max.min.radii.from.circle.s.center.of.mass | 0.68542291166334 | 0 |
| Max/min radii from hull's center of mass | Max.min.radii.from.hull.s.center.of.mass | 1 | NA |
| Max/min radii from hull's center of mass | Maximum.branch.length | 0.154895775112819 | 0 |
| Max/min radii from hull's center of mass | Maximum.radius.from.circle.s.center.of.mass | 0.180931801285188 | 0 |
| Max/min radii from hull's center of mass | Maximum.radius.from.hull.s.center.of.mass | 0.241519587056931 | 0 |
| Max/min radii from hull's center of mass | Maximum.span.across.hull | 0.194832085399412 | 0 |
| Max/min radii from hull's center of mass | Mean.radius | 0.0514474299553653 | 1.9410317797508e-10 |
| Max/min radii from hull's center of mass | Mean.radius.from.circle.s.center.of.mass | 0.0729308082441077 | 0 |
| Max/min radii from hull's center of mass | Perimeter | 0.0242994162754071 | 0.00265421793320053 |
| Max/min radii from hull's center of mass | Relative.variation..CV..in.radii.from.circle.s.center.of.mass | 0.684236175497981 | 0 |
| Max/min radii from hull's center of mass | Relative.variation..CV..in.radii.from.hull.s.center.of.mass | 0.894607591184648 | 0 |
| Max/min radii from hull's center of mass | Span.ratio.of.hull..major.minor.axis. | 0.730741695643127 | 0 |
| Max/min radii from hull's center of mass | Width.of.bounding.rectangle | 0.0277681406024391 | 0.000594031549063567 |
| Max/min radii from hull's center of mass | X..of.branches | -0.178284241725058 | 0 |
| Max/min radii from hull's center of mass | X..of.end.point.voxels | -0.166899261134277 | 0 |
| Max/min radii from hull's center of mass | X..of.junction.voxels | -0.173626064246733 | 0 |
| Max/min radii from hull's center of mass | X..of.junctions | -0.175833124815153 | 0 |
| Max/min radii from hull's center of mass | X..of.quadruple.points | -0.104877418657893 | 0 |
| Max/min radii from hull's center of mass | X..of.slab.voxels | -0.135165988108168 | 0 |
| Max/min radii from hull's center of mass | X..of.triple.points | -0.170139772631169 | 0 |
| Maximum branch length | Area | 0.376334472296143 | 0 |
| Maximum branch length | Average.branch.length | 0.528742866862097 | 0 |
| Maximum branch length | Circularity | -0.176828487074485 | 0 |
| Maximum branch length | Density.of.foreground.pixels.in.hull.area | -0.254264893886761 | 0 |
| Maximum branch length | Diameter.of.bounding.circle | 0.42522600299919 | 0 |
| Maximum branch length | Foreground.pixels | 0.346314657462417 | 0 |
| Maximum branch length | Height.of.bounding.rectangle | 0.323004796840563 | 0 |
| Maximum branch length | Max.min.radii.from.circle.s.center.of.mass | 0.0629301578497894 | 6.66133814775094e-15 |
| Maximum branch length | Max.min.radii.from.hull.s.center.of.mass | 0.154895775112819 | 0 |
| Maximum branch length | Maximum.branch.length | 1 | NA |
| Maximum branch length | Maximum.radius.from.circle.s.center.of.mass | 0.425225732947963 | 0 |
| Maximum branch length | Maximum.radius.from.hull.s.center.of.mass | 0.441642908116047 | 0 |
| Maximum branch length | Maximum.span.across.hull | 0.423028916060197 | 0 |
| Maximum branch length | Mean.radius | 0.415254170900131 | 0 |
| Maximum branch length | Mean.radius.from.circle.s.center.of.mass | 0.422179413729055 | 0 |
| Maximum branch length | Perimeter | 0.412966941909257 | 0 |
| Maximum branch length | Relative.variation..CV..in.radii.from.circle.s.center.of.mass | 0.0626269624448275 | 9.10382880192628e-15 |
| Maximum branch length | Relative.variation..CV..in.radii.from.hull.s.center.of.mass | 0.154591977764823 | 0 |
| Maximum branch length | Span.ratio.of.hull..major.minor.axis. | 0.11597090966609 | 0 |
| Maximum branch length | Width.of.bounding.rectangle | 0.368953414739361 | 0 |
| Maximum branch length | X..of.branches | 0.0165431765965601 | 0.0407777709127846 |
| Maximum branch length | X..of.end.point.voxels | 0.0457716990855284 | 1.48873786631754e-08 |
| Maximum branch length | X..of.junction.voxels | 0.00865350214513194 | 0.284589252692996 |
| Maximum branch length | X..of.junctions | 0.0096203951980501 | 0.234189804870143 |
| Maximum branch length | X..of.quadruple.points | 0.00957289446852036 | 0.236507644018474 |
| Maximum branch length | X..of.slab.voxels | 0.261737182946652 | 0 |
| Maximum branch length | X..of.triple.points | 0.0102052073181881 | 0.206964859245472 |
| Maximum radius from circle's center of mass | Area | 0.916874814041282 | 0 |
| Maximum radius from circle's center of mass | Average.branch.length | 0.0271751948169098 | 0.000776712640470079 |
| Maximum radius from circle's center of mass | Circularity | -0.289545252982585 | 0 |
| Maximum radius from circle's center of mass | Density.of.foreground.pixels.in.hull.area | -0.641172288864286 | 0 |
| Maximum radius from circle's center of mass | Diameter.of.bounding.circle | 0.999999999850699 | 0 |
| Maximum radius from circle's center of mass | Foreground.pixels | 0.821861793241616 | 0 |
| Maximum radius from circle's center of mass | Height.of.bounding.rectangle | 0.796310444680658 | 0 |
| Maximum radius from circle's center of mass | Max.min.radii.from.circle.s.center.of.mass | 0.169416033466413 | 0 |
| Maximum radius from circle's center of mass | Max.min.radii.from.hull.s.center.of.mass | 0.180931801285188 | 0 |
| Maximum radius from circle's center of mass | Maximum.branch.length | 0.425225732947963 | 0 |
| Maximum radius from circle's center of mass | Maximum.radius.from.circle.s.center.of.mass | 1 | NA |
| Maximum radius from circle's center of mass | Maximum.radius.from.hull.s.center.of.mass | 0.977501681135971 | 0 |
| Maximum radius from circle's center of mass | Maximum.span.across.hull | 0.998554636218992 | 0 |
| Maximum radius from circle's center of mass | Mean.radius | 0.979034802168741 | 0 |
| Maximum radius from circle's center of mass | Mean.radius.from.circle.s.center.of.mass | 0.981397595887087 | 0 |
| Maximum radius from circle's center of mass | Perimeter | 0.974699045934234 | 0 |
| Maximum radius from circle's center of mass | Relative.variation..CV..in.radii.from.circle.s.center.of.mass | 0.181365154705451 | 0 |
| Maximum radius from circle's center of mass | Relative.variation..CV..in.radii.from.hull.s.center.of.mass | 0.159949810396574 | 0 |
| Maximum radius from circle's center of mass | Span.ratio.of.hull..major.minor.axis. | 0.25688875996382 | 0 |
| Maximum radius from circle's center of mass | Width.of.bounding.rectangle | 0.808037567307144 | 0 |
| Maximum radius from circle's center of mass | X..of.branches | 0.699795888858169 | 0 |
| Maximum radius from circle's center of mass | X..of.end.point.voxels | 0.707721585393467 | 0 |
| Maximum radius from circle's center of mass | X..of.junction.voxels | 0.662417208599931 | 0 |
| Maximum radius from circle's center of mass | X..of.junctions | 0.686453469992875 | 0 |
| Maximum radius from circle's center of mass | X..of.quadruple.points | 0.284123621689345 | 0 |
| Maximum radius from circle's center of mass | X..of.slab.voxels | 0.856607631216895 | 0 |
| Maximum radius from circle's center of mass | X..of.triple.points | 0.683594828068555 | 0 |
| Maximum radius from hull's center of mass | Area | 0.892105690533823 | 0 |
| Maximum radius from hull's center of mass | Average.branch.length | 0.0481442091057528 | 2.57358845345834e-09 |
| Maximum radius from hull's center of mass | Circularity | -0.310504818970604 | 0 |
| Maximum radius from hull's center of mass | Density.of.foreground.pixels.in.hull.area | -0.635620954417605 | 0 |
| Maximum radius from hull's center of mass | Diameter.of.bounding.circle | 0.977501711565591 | 0 |
| Maximum radius from hull's center of mass | Foreground.pixels | 0.792855537272186 | 0 |
| Maximum radius from hull's center of mass | Height.of.bounding.rectangle | 0.778002690552097 | 0 |
| Maximum radius from hull's center of mass | Max.min.radii.from.circle.s.center.of.mass | 0.158125247696923 | 0 |
| Maximum radius from hull's center of mass | Max.min.radii.from.hull.s.center.of.mass | 0.241519587056931 | 0 |
| Maximum radius from hull's center of mass | Maximum.branch.length | 0.441642908116047 | 0 |
| Maximum radius from hull's center of mass | Maximum.radius.from.circle.s.center.of.mass | 0.977501681135971 | 0 |
| Maximum radius from hull's center of mass | Maximum.radius.from.hull.s.center.of.mass | 1 | NA |
| Maximum radius from hull's center of mass | Maximum.span.across.hull | 0.975761236869373 | 0 |
| Maximum radius from hull's center of mass | Mean.radius | 0.955237058474136 | 0 |
| Maximum radius from hull's center of mass | Mean.radius.from.circle.s.center.of.mass | 0.965114431773615 | 0 |
| Maximum radius from hull's center of mass | Perimeter | 0.952960920810686 | 0 |
| Maximum radius from hull's center of mass | Relative.variation..CV..in.radii.from.circle.s.center.of.mass | 0.160137940333613 | 0 |
| Maximum radius from hull's center of mass | Relative.variation..CV..in.radii.from.hull.s.center.of.mass | 0.246475996470464 | 0 |
| Maximum radius from hull's center of mass | Span.ratio.of.hull..major.minor.axis. | 0.256631424228537 | 0 |
| Maximum radius from hull's center of mass | Width.of.bounding.rectangle | 0.791393702759121 | 0 |
| Maximum radius from hull's center of mass | X..of.branches | 0.66431268206944 | 0 |
| Maximum radius from hull's center of mass | X..of.end.point.voxels | 0.673493102738194 | 0 |
| Maximum radius from hull's center of mass | X..of.junction.voxels | 0.629355944772793 | 0 |
| Maximum radius from hull's center of mass | X..of.junctions | 0.651560238230033 | 0 |
| Maximum radius from hull's center of mass | X..of.quadruple.points | 0.267396954187571 | 0 |
| Maximum radius from hull's center of mass | X..of.slab.voxels | 0.824098717249763 | 0 |
| Maximum radius from hull's center of mass | X..of.triple.points | 0.649464164724829 | 0 |
| Maximum span across hull | Area | 0.908497282079629 | 0 |
| Maximum span across hull | Average.branch.length | 0.0280627452134027 | 0.000518957588333535 |
| Maximum span across hull | Circularity | -0.297703467082943 | 0 |
| Maximum span across hull | Density.of.foreground.pixels.in.hull.area | -0.634501429491596 | 0 |
| Maximum span across hull | Diameter.of.bounding.circle | 0.998554630794815 | 0 |
| Maximum span across hull | Foreground.pixels | 0.815113483873455 | 0 |
| Maximum span across hull | Height.of.bounding.rectangle | 0.790068041691915 | 0 |
| Maximum span across hull | Max.min.radii.from.circle.s.center.of.mass | 0.184908722692119 | 0 |
| Maximum span across hull | Max.min.radii.from.hull.s.center.of.mass | 0.194832085399412 | 0 |
| Maximum span across hull | Maximum.branch.length | 0.423028916060197 | 0 |
| Maximum span across hull | Maximum.radius.from.circle.s.center.of.mass | 0.998554636218992 | 0 |
| Maximum span across hull | Maximum.radius.from.hull.s.center.of.mass | 0.975761236869373 | 0 |
| Maximum span across hull | Maximum.span.across.hull | 1 | NA |
| Maximum span across hull | Mean.radius | 0.973309257212305 | 0 |
| Maximum span across hull | Mean.radius.from.circle.s.center.of.mass | 0.976059355372195 | 0 |
| Maximum span across hull | Perimeter | 0.968053812971967 | 0 |
| Maximum span across hull | Relative.variation..CV..in.radii.from.circle.s.center.of.mass | 0.198592877243307 | 0 |
| Maximum span across hull | Relative.variation..CV..in.radii.from.hull.s.center.of.mass | 0.174643331347674 | 0 |
| Maximum span across hull | Span.ratio.of.hull..major.minor.axis. | 0.281643458253785 | 0 |
| Maximum span across hull | Width.of.bounding.rectangle | 0.802101602096958 | 0 |
| Maximum span across hull | X..of.branches | 0.693187194800088 | 0 |
| Maximum span across hull | X..of.end.point.voxels | 0.700951368485575 | 0 |
| Maximum span across hull | X..of.junction.voxels | 0.656177474467146 | 0 |
| Maximum span across hull | X..of.junctions | 0.680048189184638 | 0 |
| Maximum span across hull | X..of.quadruple.points | 0.28079068032621 | 0 |
| Maximum span across hull | X..of.slab.voxels | 0.848886788247582 | 0 |
| Maximum span across hull | X..of.triple.points | 0.677341549877694 | 0 |
| Mean radius | Area | 0.952420643414535 | 0 |
| Mean radius | Average.branch.length | 0.00678113912890915 | 0.401733229240904 |
| Mean radius | Circularity | -0.192322144772804 | 0 |
| Mean radius | Density.of.foreground.pixels.in.hull.area | -0.678325274391104 | 0 |
| Mean radius | Diameter.of.bounding.circle | 0.979034848836797 | 0 |
| Mean radius | Foreground.pixels | 0.84542190422765 | 0 |
| Mean radius | Height.of.bounding.rectangle | 0.812311678836164 | 0 |
| Mean radius | Max.min.radii.from.circle.s.center.of.mass | 0.0435028049299023 | 7.37132617167902e-08 |
| Mean radius | Max.min.radii.from.hull.s.center.of.mass | 0.0514474299553653 | 1.9410317797508e-10 |
| Mean radius | Maximum.branch.length | 0.415254170900131 | 0 |
| Mean radius | Maximum.radius.from.circle.s.center.of.mass | 0.979034802168741 | 0 |
| Mean radius | Maximum.radius.from.hull.s.center.of.mass | 0.955237058474136 | 0 |
| Mean radius | Maximum.span.across.hull | 0.973309257212305 | 0 |
| Mean radius | Mean.radius | 1 | NA |
| Mean radius | Mean.radius.from.circle.s.center.of.mass | 0.996236054293991 | 0 |
| Mean radius | Perimeter | 0.989920644345006 | 0 |
| Mean radius | Relative.variation..CV..in.radii.from.circle.s.center.of.mass | 0.0309430329761952 | 0.000129625140868228 |
| Mean radius | Relative.variation..CV..in.radii.from.hull.s.center.of.mass | 0.0189752406554482 | 0.0189460845331746 |
| Mean radius | Span.ratio.of.hull..major.minor.axis. | 0.134709442476949 | 0 |
| Mean radius | Width.of.bounding.rectangle | 0.823771071234386 | 0 |
| Mean radius | X..of.branches | 0.734318223494847 | 0 |
| Mean radius | X..of.end.point.voxels | 0.742911771896499 | 0 |
| Mean radius | X..of.junction.voxels | 0.695023756756056 | 0 |
| Mean radius | X..of.junctions | 0.720068119171879 | 0 |
| Mean radius | X..of.quadruple.points | 0.301698443281639 | 0 |
| Mean radius | X..of.slab.voxels | 0.888886866300003 | 0 |
| Mean radius | X..of.triple.points | 0.716553990397988 | 0 |
| Mean radius from circle's center of mass | Area | 0.948722877580332 | 0 |
| Mean radius from circle's center of mass | Average.branch.length | 0.0135702283388304 | 0.0933274601798371 |
| Mean radius from circle's center of mass | Circularity | -0.21007531574719 | 0 |
| Mean radius from circle's center of mass | Density.of.foreground.pixels.in.hull.area | -0.673994419553791 | 0 |
| Mean radius from circle's center of mass | Diameter.of.bounding.circle | 0.981397653430393 | 0 |
| Mean radius from circle's center of mass | Foreground.pixels | 0.843214317065048 | 0 |
| Mean radius from circle's center of mass | Height.of.bounding.rectangle | 0.812325406156178 | 0 |
| Mean radius from circle's center of mass | Max.min.radii.from.circle.s.center.of.mass | 0.0425270268080409 | 1.43235591387736e-07 |
| Mean radius from circle's center of mass | Max.min.radii.from.hull.s.center.of.mass | 0.0729308082441077 | 0 |
| Mean radius from circle's center of mass | Maximum.branch.length | 0.422179413729055 | 0 |
| Mean radius from circle's center of mass | Maximum.radius.from.circle.s.center.of.mass | 0.981397595887087 | 0 |
| Mean radius from circle's center of mass | Maximum.radius.from.hull.s.center.of.mass | 0.965114431773615 | 0 |
| Mean radius from circle's center of mass | Maximum.span.across.hull | 0.976059355372195 | 0 |
| Mean radius from circle's center of mass | Mean.radius | 0.996236054293991 | 0 |
| Mean radius from circle's center of mass | Mean.radius.from.circle.s.center.of.mass | 1 | NA |
| Mean radius from circle's center of mass | Perimeter | 0.98960948246191 | 0 |
| Mean radius from circle's center of mass | Relative.variation..CV..in.radii.from.circle.s.center.of.mass | 0.0252225064239138 | 0.00181232541757614 |
| Mean radius from circle's center of mass | Relative.variation..CV..in.radii.from.hull.s.center.of.mass | 0.0473695196951422 | 4.60763160958777e-09 |
| Mean radius from circle's center of mass | Span.ratio.of.hull..major.minor.axis. | 0.14603235585528 | 0 |
| Mean radius from circle's center of mass | Width.of.bounding.rectangle | 0.822854537741712 | 0 |
| Mean radius from circle's center of mass | X..of.branches | 0.726649517518614 | 0 |
| Mean radius from circle's center of mass | X..of.end.point.voxels | 0.734619882626655 | 0 |
| Mean radius from circle's center of mass | X..of.junction.voxels | 0.687900972051311 | 0 |
| Mean radius from circle's center of mass | X..of.junctions | 0.712711182617409 | 0 |
| Mean radius from circle's center of mass | X..of.quadruple.points | 0.297822595303561 | 0 |
| Mean radius from circle's center of mass | X..of.slab.voxels | 0.882671493394647 | 0 |
| Mean radius from circle's center of mass | X..of.triple.points | 0.709378230542683 | 0 |
| Perimeter | Area | 0.978815558151304 | 0 |
| Perimeter | Average.branch.length | 0.000113153617280275 | 0.988836376582857 |
| Perimeter | Circularity | -0.119019524586443 | 0 |
| Perimeter | Density.of.foreground.pixels.in.hull.area | -0.675527881586245 | 0 |
| Perimeter | Diameter.of.bounding.circle | 0.974699116654495 | 0 |
| Perimeter | Foreground.pixels | 0.88079282107545 | 0 |
| Perimeter | Height.of.bounding.rectangle | 0.82714544485697 | 0 |
| Perimeter | Max.min.radii.from.circle.s.center.of.mass | 0.0263691968166292 | 0.00110924633183385 |
| Perimeter | Max.min.radii.from.hull.s.center.of.mass | 0.0242994162754071 | 0.00265421793320053 |
| Perimeter | Maximum.branch.length | 0.412966941909257 | 0 |
| Perimeter | Maximum.radius.from.circle.s.center.of.mass | 0.974699045934234 | 0 |
| Perimeter | Maximum.radius.from.hull.s.center.of.mass | 0.952960920810686 | 0 |
| Perimeter | Maximum.span.across.hull | 0.968053812971967 | 0 |
| Perimeter | Mean.radius | 0.989920644345006 | 0 |
| Perimeter | Mean.radius.from.circle.s.center.of.mass | 0.98960948246191 | 0 |
| Perimeter | Perimeter | 1 | NA |
| Perimeter | Relative.variation..CV..in.radii.from.circle.s.center.of.mass | 0.0227556252161271 | 0.00488979167757164 |
| Perimeter | Relative.variation..CV..in.radii.from.hull.s.center.of.mass | 0.00464940367003265 | 0.565342692010518 |
| Perimeter | Span.ratio.of.hull..major.minor.axis. | 0.0685617947499011 | 0 |
| Perimeter | Width.of.bounding.rectangle | 0.827739979346128 | 0 |
| Perimeter | X..of.branches | 0.763636114848252 | 0 |
| Perimeter | X..of.end.point.voxels | 0.770805306557271 | 0 |
| Perimeter | X..of.junction.voxels | 0.72312969391464 | 0 |
| Perimeter | X..of.junctions | 0.74890155809533 | 0 |
| Perimeter | X..of.quadruple.points | 0.316185353394684 | 0 |
| Perimeter | X..of.slab.voxels | 0.92001543488791 | 0 |
| Perimeter | X..of.triple.points | 0.744735312630939 | 0 |
| Relative variation (CV) in radii from circle's center of mass | Area | -0.0779101836195323 | 0 |
| Relative variation (CV) in radii from circle's center of mass | Average.branch.length | 0.0817154463979024 | 0 |
| Relative variation (CV) in radii from circle's center of mass | Circularity | -0.545803098154305 | 0 |
| Relative variation (CV) in radii from circle's center of mass | Density.of.foreground.pixels.in.hull.area | 0.0859501476204992 | 0 |
| Relative variation (CV) in radii from circle's center of mass | Diameter.of.bounding.circle | 0.181365039425767 | 0 |
| Relative variation (CV) in radii from circle's center of mass | Foreground.pixels | -0.0514627884621109 | 1.91710869401618e-10 |
| Relative variation (CV) in radii from circle's center of mass | Height.of.bounding.rectangle | -0.0141890273719586 | 0.0793227682495066 |
| Relative variation (CV) in radii from circle's center of mass | Max.min.radii.from.circle.s.center.of.mass | 0.915805546678529 | 0 |
| Relative variation (CV) in radii from circle's center of mass | Max.min.radii.from.hull.s.center.of.mass | 0.684236175497981 | 0 |
| Relative variation (CV) in radii from circle's center of mass | Maximum.branch.length | 0.0626269624448275 | 9.10382880192628e-15 |
| Relative variation (CV) in radii from circle's center of mass | Maximum.radius.from.circle.s.center.of.mass | 0.181365154705451 | 0 |
| Relative variation (CV) in radii from circle's center of mass | Maximum.radius.from.hull.s.center.of.mass | 0.160137940333613 | 0 |
| Relative variation (CV) in radii from circle's center of mass | Maximum.span.across.hull | 0.198592877243307 | 0 |
| Relative variation (CV) in radii from circle's center of mass | Mean.radius | 0.0309430329761952 | 0.000129625140868228 |
| Relative variation (CV) in radii from circle's center of mass | Mean.radius.from.circle.s.center.of.mass | 0.0252225064239138 | 0.00181232541757614 |
| Relative variation (CV) in radii from circle's center of mass | Perimeter | 0.0227556252161271 | 0.00488979167757164 |
| Relative variation (CV) in radii from circle's center of mass | Relative.variation..CV..in.radii.from.circle.s.center.of.mass | 1 | NA |
| Relative variation (CV) in radii from circle's center of mass | Relative.variation..CV..in.radii.from.hull.s.center.of.mass | 0.661421513508915 | 0 |
| Relative variation (CV) in radii from circle's center of mass | Span.ratio.of.hull..major.minor.axis. | 0.648803998030366 | 0 |
| Relative variation (CV) in radii from circle's center of mass | Width.of.bounding.rectangle | 0.0142407239545315 | 0.0782345760473135 |
| Relative variation (CV) in radii from circle's center of mass | X..of.branches | -0.0795914446244367 | 0 |
| Relative variation (CV) in radii from circle's center of mass | X..of.end.point.voxels | -0.0769498548315971 | 0 |
| Relative variation (CV) in radii from circle's center of mass | X..of.junction.voxels | -0.0768297475910305 | 0 |
| Relative variation (CV) in radii from circle's center of mass | X..of.junctions | -0.0781523370079931 | 0 |
| Relative variation (CV) in radii from circle's center of mass | X..of.quadruple.points | -0.0475437913725715 | 4.04500166695243e-09 |
| Relative variation (CV) in radii from circle's center of mass | X..of.slab.voxels | -0.0568526193128191 | 1.98152605435098e-12 |
| Relative variation (CV) in radii from circle's center of mass | X..of.triple.points | -0.0757751523151772 | 0 |
| Relative variation (CV) in radii from hull's center of mass | Area | -0.11677557245703 | 0 |
| Relative variation (CV) in radii from hull's center of mass | Average.branch.length | 0.166462517943933 | 0 |
| Relative variation (CV) in radii from hull's center of mass | Circularity | -0.678997159078028 | 0 |
| Relative variation (CV) in radii from hull's center of mass | Density.of.foreground.pixels.in.hull.area | 0.0435792410880904 | 6.99334978815358e-08 |
| Relative variation (CV) in radii from hull's center of mass | Diameter.of.bounding.circle | 0.15994972896523 | 0 |
| Relative variation (CV) in radii from hull's center of mass | Foreground.pixels | -0.12643948779324 | 0 |
| Relative variation (CV) in radii from hull's center of mass | Height.of.bounding.rectangle | -0.0330367843930348 | 4.38361694825584e-05 |
| Relative variation (CV) in radii from hull's center of mass | Max.min.radii.from.circle.s.center.of.mass | 0.60442572878505 | 0 |
| Relative variation (CV) in radii from hull's center of mass | Max.min.radii.from.hull.s.center.of.mass | 0.894607591184648 | 0 |
| Relative variation (CV) in radii from hull's center of mass | Maximum.branch.length | 0.154591977764823 | 0 |
| Relative variation (CV) in radii from hull's center of mass | Maximum.radius.from.circle.s.center.of.mass | 0.159949810396574 | 0 |
| Relative variation (CV) in radii from hull's center of mass | Maximum.radius.from.hull.s.center.of.mass | 0.246475996470464 | 0 |
| Relative variation (CV) in radii from hull's center of mass | Maximum.span.across.hull | 0.174643331347674 | 0 |
| Relative variation (CV) in radii from hull's center of mass | Mean.radius | 0.0189752406554482 | 0.0189460845331746 |
| Relative variation (CV) in radii from hull's center of mass | Mean.radius.from.circle.s.center.of.mass | 0.0473695196951422 | 4.60763160958777e-09 |
| Relative variation (CV) in radii from hull's center of mass | Perimeter | 0.00464940367003265 | 0.565342692010518 |
| Relative variation (CV) in radii from hull's center of mass | Relative.variation..CV..in.radii.from.circle.s.center.of.mass | 0.661421513508915 | 0 |
| Relative variation (CV) in radii from hull's center of mass | Relative.variation..CV..in.radii.from.hull.s.center.of.mass | 1 | NA |
| Relative variation (CV) in radii from hull's center of mass | Span.ratio.of.hull..major.minor.axis. | 0.676141195094325 | 0 |
| Relative variation (CV) in radii from hull's center of mass | Width.of.bounding.rectangle | 0.00649549688439771 | 0.421854759586934 |
| Relative variation (CV) in radii from hull's center of mass | X..of.branches | -0.190133893063063 | 0 |
| Relative variation (CV) in radii from hull's center of mass | X..of.end.point.voxels | -0.178280302020069 | 0 |
| Relative variation (CV) in radii from hull's center of mass | X..of.junction.voxels | -0.182106906902988 | 0 |
| Relative variation (CV) in radii from hull's center of mass | X..of.junctions | -0.1877640943637 | 0 |
| Relative variation (CV) in radii from hull's center of mass | X..of.quadruple.points | -0.109067704761233 | 0 |
| Relative variation (CV) in radii from hull's center of mass | X..of.slab.voxels | -0.147072581510514 | 0 |
| Relative variation (CV) in radii from hull's center of mass | X..of.triple.points | -0.182261489676345 | 0 |
| Span ratio of hull (major/minor axis) | Area | -0.0802447334970643 | 0 |
| Span ratio of hull (major/minor axis) | Average.branch.length | 0.102182140868491 | 0 |
| Span ratio of hull (major/minor axis) | Circularity | -0.810826657960008 | 0 |
| Span ratio of hull (major/minor axis) | Density.of.foreground.pixels.in.hull.area | 0.0304276325112855 | 0.00016762349709043 |
| Span ratio of hull (major/minor axis) | Diameter.of.bounding.circle | 0.256888512538767 | 0 |
| Span ratio of hull (major/minor axis) | Foreground.pixels | -0.0832079357437125 | 0 |
| Span ratio of hull (major/minor axis) | Height.of.bounding.rectangle | 0.0166481829014438 | 0.0395163008648667 |
| Span ratio of hull (major/minor axis) | Max.min.radii.from.circle.s.center.of.mass | 0.618403626910029 | 0 |
| Span ratio of hull (major/minor axis) | Max.min.radii.from.hull.s.center.of.mass | 0.730741695643127 | 0 |
| Span ratio of hull (major/minor axis) | Maximum.branch.length | 0.11597090966609 | 0 |
| Span ratio of hull (major/minor axis) | Maximum.radius.from.circle.s.center.of.mass | 0.25688875996382 | 0 |
| Span ratio of hull (major/minor axis) | Maximum.radius.from.hull.s.center.of.mass | 0.256631424228537 | 0 |
| Span ratio of hull (major/minor axis) | Maximum.span.across.hull | 0.281643458253785 | 0 |
| Span ratio of hull (major/minor axis) | Mean.radius | 0.134709442476949 | 0 |
| Span ratio of hull (major/minor axis) | Mean.radius.from.circle.s.center.of.mass | 0.14603235585528 | 0 |
| Span ratio of hull (major/minor axis) | Perimeter | 0.0685617947499011 | 0 |
| Span ratio of hull (major/minor axis) | Relative.variation..CV..in.radii.from.circle.s.center.of.mass | 0.648803998030366 | 0 |
| Span ratio of hull (major/minor axis) | Relative.variation..CV..in.radii.from.hull.s.center.of.mass | 0.676141195094325 | 0 |
| Span ratio of hull (major/minor axis) | Span.ratio.of.hull..major.minor.axis. | 1 | NA |
| Span ratio of hull (major/minor axis) | Width.of.bounding.rectangle | 0.055054349419587 | 9.5652374909605e-12 |
| Span ratio of hull (major/minor axis) | X..of.branches | -0.126754352604664 | 0 |
| Span ratio of hull (major/minor axis) | X..of.end.point.voxels | -0.122364182325642 | 0 |
| Span ratio of hull (major/minor axis) | X..of.junction.voxels | -0.120934710649621 | 0 |
| Span ratio of hull (major/minor axis) | X..of.junctions | -0.123971118900431 | 0 |
| Span ratio of hull (major/minor axis) | X..of.quadruple.points | -0.0722421443840065 | 0 |
| Span ratio of hull (major/minor axis) | X..of.slab.voxels | -0.10181994175478 | 0 |
| Span ratio of hull (major/minor axis) | X..of.triple.points | -0.119926020441094 | 0 |
| Width of bounding rectangle | Area | 0.807279424177353 | 0 |
| Width of bounding rectangle | Average.branch.length | 0.0186191609364788 | 0.0213043169789211 |
| Width of bounding rectangle | Circularity | -0.106510868227047 | 0 |
| Width of bounding rectangle | Density.of.foreground.pixels.in.hull.area | -0.554196624245954 | 0 |
| Width of bounding rectangle | Diameter.of.bounding.circle | 0.808037533311594 | 0 |
| Width of bounding rectangle | Foreground.pixels | 0.731479752471551 | 0 |
| Width of bounding rectangle | Height.of.bounding.rectangle | 0.425217807875554 | 0 |
| Width of bounding rectangle | Max.min.radii.from.circle.s.center.of.mass | 0.023487831316039 | 0.00367507951661405 |
| Width of bounding rectangle | Max.min.radii.from.hull.s.center.of.mass | 0.0277681406024391 | 0.000594031549063567 |
| Width of bounding rectangle | Maximum.branch.length | 0.368953414739361 | 0 |
| Width of bounding rectangle | Maximum.radius.from.circle.s.center.of.mass | 0.808037567307144 | 0 |
| Width of bounding rectangle | Maximum.radius.from.hull.s.center.of.mass | 0.791393702759121 | 0 |
| Width of bounding rectangle | Maximum.span.across.hull | 0.802101602096958 | 0 |
| Width of bounding rectangle | Mean.radius | 0.823771071234386 | 0 |
| Width of bounding rectangle | Mean.radius.from.circle.s.center.of.mass | 0.822854537741712 | 0 |
| Width of bounding rectangle | Perimeter | 0.827739979346128 | 0 |
| Width of bounding rectangle | Relative.variation..CV..in.radii.from.circle.s.center.of.mass | 0.0142407239545315 | 0.0782345760473135 |
| Width of bounding rectangle | Relative.variation..CV..in.radii.from.hull.s.center.of.mass | 0.00649549688439771 | 0.421854759586934 |
| Width of bounding rectangle | Span.ratio.of.hull..major.minor.axis. | 0.055054349419587 | 9.5652374909605e-12 |
| Width of bounding rectangle | Width.of.bounding.rectangle | 1 | NA |
| Width of bounding rectangle | X..of.branches | 0.618387936993836 | 0 |
| Width of bounding rectangle | X..of.end.point.voxels | 0.622279772552397 | 0 |
| Width of bounding rectangle | X..of.junction.voxels | 0.587799270607362 | 0 |
| Width of bounding rectangle | X..of.junctions | 0.607050627414771 | 0 |
| Width of bounding rectangle | X..of.quadruple.points | 0.259090641138724 | 0 |
| Width of bounding rectangle | X..of.slab.voxels | 0.753325541487284 | 0 |
| Width of bounding rectangle | X..of.triple.points | 0.603963087727479 | 0 |
