# Supplementary material for: Development of a High-Throughput Pipeline to Characterize Microglia Morphological States at a Single-Cell Resolution
Source: eNeuro. 2024 Jul 26;11(7):ENEURO.0014-24.2024. doi: 10.1523/ENEURO.0014-24.2024 (PMC11289588; doi:10.1523/ENEURO.0014-24.2024)
Supplement: Table 3-3 — Spearman’s correlation of morphology measures to principal components and p-values for correlations, related to Fig. 3-1B. Download Table 3-3, DOC file. [file eneuro-11-ENEURO.0014-24.2024-s005.doc]

| **measure** | **PC** | **correlation** | **pvalues** |
| --- | --- | --- | --- |
| # of branches | PC1 | -0.871480555005847 | 0 |
| # of branches | PC2 | -0.293736379280793 | 0 |
| # of branches | PC3 | -0.248188056041827 | 0 |
| # of end point voxels | PC1 | -0.858664406455125 | 0 |
| # of end point voxels | PC2 | -0.25653050119569 | 0 |
| # of end point voxels | PC3 | -0.179598650299587 | 0 |
| # of junction voxels | PC1 | -0.833345318275591 | 0 |
| # of junction voxels | PC2 | -0.29271346418242 | 0 |
| # of junction voxels | PC3 | -0.261883847897882 | 0 |
| # of junctions | PC1 | -0.857945660673511 | 0 |
| # of junctions | PC2 | -0.294280534761895 | 0 |
| # of junctions | PC3 | -0.258323155749275 | 0 |
| # of quadruple points | PC1 | -0.387375603410933 | 0 |
| # of quadruple points | PC2 | -0.183311539380111 | 0 |
| # of quadruple points | PC3 | -0.150932397288395 | 0 |
| # of slab voxels | PC1 | -0.964797062376466 | 0 |
| # of slab voxels | PC2 | -0.152915549774359 | 0 |
| # of slab voxels | PC3 | 0.0423863028176816 | 1.57452409776937e-07 |
| # of triple points | PC1 | -0.849449274507994 | 0 |
| # of triple points | PC2 | -0.284725333842969 | 0 |
| # of triple points | PC3 | -0.251619918003768 | 0 |
| Area | PC1 | -0.970332743285711 | 0 |
| Area | PC2 | -0.061298215398869 | 3.28626015289046e-14 |
| Area | PC3 | 0.230548509675001 | 0 |
| Average branch length | PC1 | 0.164264116883958 | 0 |
| Average branch length | PC2 | 0.419315205099746 | 0 |
| Average branch length | PC3 | 0.75052137459093 | 0 |
| Circularity | PC1 | 0.0695107266896089 | 0 |
| Circularity | PC2 | -0.824779523419996 | 0 |
| Circularity | PC3 | 0.201094545028387 | 0 |
| Density of foreground pixels in hull area | PC1 | 0.692403006499036 | 0 |
| Density of foreground pixels in hull area | PC2 | 0.0356677828501929 | 1.02469854899034e-05 |
| Density of foreground pixels in hull area | PC3 | -0.0596527799089705 | 1.54987134237672e-13 |
| Diameter of bounding circle | PC1 | -0.946757911232905 | 0 |
| Diameter of bounding circle | PC2 | 0.265076464599541 | 0 |
| Diameter of bounding circle | PC3 | 0.122914783026015 | 0 |
| Foreground pixels | PC1 | -0.859834417519362 | 0 |
| Foreground pixels | PC2 | -0.0567176593666753 | 2.23376872554581e-12 |
| Foreground pixels | PC3 | 0.266440951092268 | 0 |
| Height of bounding rectangle | PC1 | -0.819114528986685 | 0 |
| Height of bounding rectangle | PC2 | 0.0246445404810762 | 0.00230463746620502 |
| Height of bounding rectangle | PC3 | 0.158704143650433 | 0 |
| Max/min radii from circle's center of mass | PC1 | -0.0185377440844915 | 0.0218781163671422 |
| Max/min radii from circle's center of mass | PC2 | 0.730926422831284 | 0 |
| Max/min radii from circle's center of mass | PC3 | -0.414706340567187 | 0 |
| Max/min radii from hull's center of mass | PC1 | 0.0110235814687755 | 0.172832169280346 |
| Max/min radii from hull's center of mass | PC2 | 0.865254564172484 | 0 |
| Max/min radii from hull's center of mass | PC3 | -0.298820346641885 | 0 |
| Maximum branch length | PC1 | -0.319624609916512 | 0 |
| Maximum branch length | PC2 | 0.346665701577124 | 0 |
| Maximum branch length | PC3 | 0.616691815537366 | 0 |
| Maximum radius from circle's center of mass | PC1 | -0.946757913453376 | 0 |
| Maximum radius from circle's center of mass | PC2 | 0.26507652325091 | 0 |
| Maximum radius from circle's center of mass | PC3 | 0.122914415682652 | 0 |
| Maximum radius from hull's center of mass | PC1 | -0.921302314943042 | 0 |
| Maximum radius from hull's center of mass | PC2 | 0.300307903158382 | 0 |
| Maximum radius from hull's center of mass | PC3 | 0.133131357202536 | 0 |
| Maximum span across hull | PC1 | -0.940904596100212 | 0 |
| Maximum span across hull | PC2 | 0.281318368040241 | 0 |
| Maximum span across hull | PC3 | 0.112812233725542 | 0 |
| Mean radius | PC1 | -0.964468805890706 | 0 |
| Mean radius | PC2 | 0.13206278796186 | 0 |
| Mean radius | PC3 | 0.190013205519431 | 0 |
| Mean radius from circle's center of mass | PC1 | -0.962002260926287 | 0 |
| Mean radius from circle's center of mass | PC2 | 0.149096332956416 | 0 |
| Mean radius from circle's center of mass | PC3 | 0.192902710828745 | 0 |
| Perimeter | PC1 | -0.978132117122523 | 0 |
| Perimeter | PC2 | 0.0880167995680848 | 0 |
| Perimeter | PC3 | 0.195316833213491 | 0 |
| Relative variation (CV) in radii from circle's center of mass | PC1 | -0.016250172611905 | 0.0444794848094063 |
| Relative variation (CV) in radii from circle's center of mass | PC2 | 0.746715751812176 | 0 |
| Relative variation (CV) in radii from circle's center of mass | PC3 | -0.421031365741703 | 0 |
| Relative variation (CV) in radii from hull's center of mass | PC1 | 0.0278941170624692 | 0.000560770101591501 |
| Relative variation (CV) in radii from hull's center of mass | PC2 | 0.835451774423931 | 0 |
| Relative variation (CV) in radii from hull's center of mass | PC3 | -0.274267464493687 | 0 |
| Span ratio of hull (major/minor axis) | PC1 | -0.0435101204017764 | 7.33431182453614e-08 |
| Span ratio of hull (major/minor axis) | PC2 | 0.822427741454663 | 0 |
| Span ratio of hull (major/minor axis) | PC3 | -0.291601609696578 | 0 |
| Width of bounding rectangle | PC1 | -0.805921112208541 | 0 |
| Width of bounding rectangle | PC2 | 0.0851501300453201 | 0 |
| Width of bounding rectangle | PC3 | 0.192987874767246 | 0 |
