# Supplementary material for: Development of a High-Throughput Pipeline to Characterize Microglia Morphological States at a Single-Cell Resolution
Source: eNeuro. 2024 Jul 26;11(7):ENEURO.0014-24.2024. doi: 10.1523/ENEURO.0014-24.2024 (PMC11289588; doi:10.1523/ENEURO.0014-24.2024)
Supplement: Table 3-4 — Spearman’s correlation of morphology measures to principal components and p-values for correlations, related to Fig. 3B. Download Table 3-4, DOC file. [file eneuro-11-ENEURO.0014-24.2024-s006.doc]

| **measure** | **PC** | **correlation** | **pvalues** |
| --- | --- | --- | --- |
| # of branches | PC1 | 0.920237132053563 | 0 |
| # of branches | PC2 | 0.192273703666935 | 0 |
| # of branches | PC3 | -0.211568307499127 | 0 |
| # of end point voxels | PC1 | 0.906330134164387 | 0 |
| # of end point voxels | PC2 | 0.164456962074272 | 0 |
| # of end point voxels | PC3 | -0.146599505005082 | 0 |
| # of junction voxels | PC1 | 0.892884739371969 | 0 |
| # of junction voxels | PC2 | 0.195401009976912 | 0 |
| # of junction voxels | PC3 | -0.224859064098417 | 0 |
| # of junctions | PC1 | 0.909671233350955 | 0 |
| # of junctions | PC2 | 0.193784046398299 | 0 |
| # of junctions | PC3 | -0.221484091110553 | 0 |
| # of quadruple points | PC1 | 0.492020140859873 | 0 |
| # of quadruple points | PC2 | 0.148899965777247 | 0 |
| # of quadruple points | PC3 | -0.160838818957785 | 0 |
| # of slab voxels | PC1 | 0.977936901128987 | 0 |
| # of slab voxels | PC2 | 0.0934245250682899 | 0 |
| # of slab voxels | PC3 | 0.0329881069874277 | 6.48414655302076e-12 |
| # of triple points | PC1 | 0.902221530667145 | 0 |
| # of triple points | PC2 | 0.187063419234673 | 0 |
| # of triple points | PC3 | -0.216387689984879 | 0 |
| Area | PC1 | 0.983055882335522 | 0 |
| Area | PC2 | 0.0252278327171489 | 1.50296634693348e-07 |
| Area | PC3 | 0.177091676525324 | 0 |
| Average branch length | PC1 | -0.131677627526092 | 0 |
| Average branch length | PC2 | -0.337251862426824 | 0 |
| Average branch length | PC3 | 0.783666032310473 | 0 |
| Circularity | PC1 | 0.0430699819151156 | 0 |
| Circularity | PC2 | 0.842094739155755 | 0 |
| Circularity | PC3 | 0.131435299896654 | 0 |
| Density of foreground pixels in hull area | PC1 | -0.605275639361569 | 0 |
| Density of foreground pixels in hull area | PC2 | 0.0267967230539497 | 2.41991862104385e-08 |
| Density of foreground pixels in hull area | PC3 | -0.0903046444084214 | 0 |
| Diameter of bounding circle | PC1 | 0.955286625020026 | 0 |
| Diameter of bounding circle | PC2 | -0.251975079312947 | 0 |
| Diameter of bounding circle | PC3 | 0.112880000888593 | 0 |
| Foreground pixels | PC1 | 0.910869748040197 | 0 |
| Foreground pixels | PC2 | 0.0394200109178905 | 2.22044604925031e-16 |
| Foreground pixels | PC3 | 0.175924255989513 | 0 |
| Height of bounding rectangle | PC1 | 0.859673593607054 | 0 |
| Height of bounding rectangle | PC2 | -0.0915036213957373 | 0 |
| Height of bounding rectangle | PC3 | 0.14169755468279 | 0 |
| Max/min radii from circle's center of mass | PC1 | -0.0142109727338963 | 0.00309372627888971 |
| Max/min radii from circle's center of mass | PC2 | -0.764968663078815 | 0 |
| Max/min radii from circle's center of mass | PC3 | -0.393880170677139 | 0 |
| Max/min radii from hull's center of mass | PC1 | -0.053324248145669 | 0 |
| Max/min radii from hull's center of mass | PC2 | -0.887881194427616 | 0 |
| Max/min radii from hull's center of mass | PC3 | -0.262001251083863 | 0 |
| Maximum branch length | PC1 | 0.363596307270013 | 0 |
| Maximum branch length | PC2 | -0.292154345495207 | 0 |
| Maximum branch length | PC3 | 0.632704309241877 | 0 |
| Maximum radius from circle's center of mass | PC1 | 0.955286624596387 | 0 |
| Maximum radius from circle's center of mass | PC2 | -0.251975099437734 | 0 |
| Maximum radius from circle's center of mass | PC3 | 0.112879941815907 | 0 |
| Maximum radius from hull's center of mass | PC1 | 0.938453897837612 | 0 |
| Maximum radius from hull's center of mass | PC2 | -0.277613529317917 | 0 |
| Maximum radius from hull's center of mass | PC3 | 0.124746857846987 | 0 |
| Maximum span across hull | PC1 | 0.950766038399995 | 0 |
| Maximum span across hull | PC2 | -0.265858948305012 | 0 |
| Maximum span across hull | PC3 | 0.105575163989028 | 0 |
| Mean radius | PC1 | 0.96974197448933 | 0 |
| Mean radius | PC2 | -0.140444755388277 | 0 |
| Mean radius | PC3 | 0.166463841426797 | 0 |
| Mean radius from circle's center of mass | PC1 | 0.967740654181372 | 0 |
| Mean radius from circle's center of mass | PC2 | -0.152980596812294 | 0 |
| Mean radius from circle's center of mass | PC3 | 0.168756185032873 | 0 |
| Perimeter | PC1 | 0.982810497680131 | 0 |
| Perimeter | PC2 | -0.0983272017184234 | 0 |
| Perimeter | PC3 | 0.160971591283634 | 0 |
| Relative variation (CV) in radii from circle's center of mass | PC1 | -0.0207270140568113 | 1.59642867232357e-05 |
| Relative variation (CV) in radii from circle's center of mass | PC2 | -0.778666320382796 | 0 |
| Relative variation (CV) in radii from circle's center of mass | PC3 | -0.40267968526188 | 0 |
| Relative variation (CV) in radii from hull's center of mass | PC1 | -0.0565084851578885 | 0 |
| Relative variation (CV) in radii from hull's center of mass | PC2 | -0.863743377318137 | 0 |
| Relative variation (CV) in radii from hull's center of mass | PC3 | -0.246272447480937 | 0 |
| Span ratio of hull (major/minor axis) | PC1 | -0.0289964181361475 | 1.56920076932465e-09 |
| Span ratio of hull (major/minor axis) | PC2 | -0.840185525942318 | 0 |
| Span ratio of hull (major/minor axis) | PC3 | -0.230020442952741 | 0 |
| Width of bounding rectangle | PC1 | 0.855618442685151 | 0 |
| Width of bounding rectangle | PC2 | -0.0354316648986081 | 1.61204383175573e-13 |
| Width of bounding rectangle | PC3 | 0.16626466135632 | 0 |
