# Supplementary material for: Development of a High-Throughput Pipeline to Characterize Microglia Morphological States at a Single-Cell Resolution
Source: eNeuro. 2024 Jul 26;11(7):ENEURO.0014-24.2024. doi: 10.1523/ENEURO.0014-24.2024 (PMC11289588; doi:10.1523/ENEURO.0014-24.2024)
Supplement: Table 4-1 — Analysis of Deviance (Type II Wald chisquare tests) on models fit for each brain region separately: percentage ∼ Cluster*Treatment*Antibody + (1|MouseID). Significance denoted at Pr(>Chisq) < 0.05, related to Fig. 4A. Download Table 4-1, DOC file. [file eneuro-11-ENEURO.0014-24.2024-s007.doc]

| **Variable** | **Chisq** | **Df** | **Pr(>Chisq)** | **BrainRegion** | **Significant** |
| --- | --- | --- | --- | --- | --- |
| Cluster | 49.7285140665214 | 3 | 9.12677015946535e-11 | FC | significant |
| Treatment | 0.0845433206487355 | 1 | 0.771232323945939 | FC | ns |
| Antibody | 0.0818161670256428 | 2 | 0.959917358124465 | FC | ns |
| Cluster:Treatment | 202.155753796447 | 3 | 1.4432831254405e-43 | FC | significant |
| Cluster:Antibody | 86.420605428497 | 6 | 1.67588066751616e-16 | FC | significant |
| Treatment:Antibody | 0.538869286006519 | 2 | 0.763811198297073 | FC | ns |
| Cluster:Treatment:Antibody | 17.6762500588745 | 6 | 0.00709430350874582 | FC | significant |
| Cluster | 125.632490851815 | 3 | 4.72235673659333e-27 | HC | significant |
| Treatment | 1.89046324142923 | 1 | 0.169149681875713 | HC | ns |
| Antibody | 0.0530320317021195 | 2 | 0.973832446955209 | HC | ns |
| Cluster:Treatment | 407.066599489392 | 3 | 6.52299157607702e-88 | HC | significant |
| Cluster:Antibody | 206.593153498556 | 6 | 7.48887797164022e-42 | HC | significant |
| Treatment:Antibody | 1.28423394586188 | 2 | 0.526177340971443 | HC | ns |
| Cluster:Treatment:Antibody | 50.6835411765993 | 6 | 3.4284217109684e-09 | HC | significant |
| Cluster | 1387.9805313473 | 3 | 1.19481710492323e-300 | STR | significant |
| Treatment | 0.0482442826350013 | 1 | 0.826147009889138 | STR | ns |
| Antibody | 0.173531587168085 | 2 | 0.916891812433355 | STR | ns |
| Cluster:Treatment | 661.629269993377 | 3 | 4.38461862540658e-143 | STR | significant |
| Cluster:Antibody | 649.183180169491 | 6 | 5.70143283350703e-137 | STR | significant |
| Treatment:Antibody | 5.01522754735809 | 2 | 0.0814623952103403 | STR | ns |
| Cluster:Treatment:Antibody | 77.4019297971236 | 6 | 1.22809532950723e-14 | STR | significant |
