# Supplementary material for: Development of a High-Throughput Pipeline to Characterize Microglia Morphological States at a Single-Cell Resolution
Source: eNeuro. 2024 Jul 26;11(7):ENEURO.0014-24.2024. doi: 10.1523/ENEURO.0014-24.2024 (PMC11289588; doi:10.1523/ENEURO.0014-24.2024)
Supplement: Table 4-2 — Tests between treatments across clusters and antibodies (∼Treatment|Cluster|Antibody), bonferroni-corrected for each brain region. Significance denoted at adjusted p-values (or q-values) < 0.05, related to Fig. 4A. Download Table 4-2, DOC file. [file eneuro-11-ENEURO.0014-24.2024-s008.doc]

| **contrast** | **Cluster** | **Antibody** | **estimate** | **SE** | **df** | **z.ratio** | **p.adjust** | **Significant** | **Brain Region** |
| --- | --- | --- | --- | --- | --- | --- | --- | --- | --- |
| PBS - 2xLPS | Ameboid | Cx3cr1 | 0.408984303167918 | 0.182158529848517 | Inf | 2.24521082547179 | 0.297055217666745 | ns | FC |
| PBS - 2xLPS | Hypertrophic | Cx3cr1 | -1.05981698349712 | 0.168260028652653 | Inf | -6.2986853858497 | 3.60216674821854e-09 | significant | FC |
| PBS - 2xLPS | Ramified | Cx3cr1 | 0.463212336476226 | 0.139003170141467 | Inf | 3.332386851356 | 0.0103325370568986 | significant | FC |
| PBS - 2xLPS | Rod-like | Cx3cr1 | 0.0621153963441361 | 0.147880052828592 | Inf | 0.420039046213583 | 1 | ns | FC |
| PBS - 2xLPS | Ameboid | Iba1 | 0.57030426541011 | 0.171403438621293 | Inf | 3.32726268502797 | 0.0105244386996013 | significant | FC |
| PBS - 2xLPS | Hypertrophic | Iba1 | -1.25871426104411 | 0.168067402255251 | Inf | -7.48934203869262 | 8.30636829655013e-13 | significant | FC |
| PBS - 2xLPS | Ramified | Iba1 | 0.576820843641583 | 0.148019978249408 | Inf | 3.89691209567442 | 0.00116912353693907 | significant | FC |
| PBS - 2xLPS | Rod-like | Iba1 | 0.0580636397772473 | 0.145425463937151 | Inf | 0.399267351158946 | 1 | ns | FC |
| PBS - 2xLPS | Ameboid | P2ry12 | 0.991251961745804 | 0.150627441321449 | Inf | 6.58081922556465 | 5.61435785862224e-10 | significant | FC |
| PBS - 2xLPS | Hypertrophic | P2ry12 | -1.05062276954499 | 0.172887708777594 | Inf | -6.07690839894543 | 1.47026044065641e-08 | significant | FC |
| PBS - 2xLPS | Ramified | P2ry12 | -0.0045744601750782 | 0.148767846900953 | Inf | -0.0307489842084211 | 1 | ns | FC |
| PBS - 2xLPS | Rod-like | P2ry12 | -0.183824092030003 | 0.156223261983146 | Inf | -1.17667554560367 | 1 | ns | FC |
| PBS - 2xLPS | Ameboid | Cx3cr1 | 0.4641043746017 | 0.134127199699362 | Inf | 3.4601809002347 | 0.00647775129477646 | significant | HC |
| PBS - 2xLPS | Hypertrophic | Cx3cr1 | -1.43545619005833 | 0.141185906171085 | Inf | -10.1671351552532 | 3.3355677625e-23 | significant | HC |
| PBS - 2xLPS | Ramified | Cx3cr1 | 0.50478060384512 | 0.103678093424382 | Inf | 4.86872961464403 | 1.34781555583662e-05 | significant | HC |
| PBS - 2xLPS | Rod-like | Cx3cr1 | 0.231274819257456 | 0.108983486303338 | Inf | 2.12210883595464 | 0.405943194885458 | ns | HC |
| PBS - 2xLPS | Ameboid | Iba1 | 0.393700534316134 | 0.130471935272532 | Inf | 3.01751126396466 | 0.0305831483169448 | significant | HC |
| PBS - 2xLPS | Hypertrophic | Iba1 | -0.988900486041881 | 0.113997367600096 | Inf | -8.67476597802638 | 4.97282144755315e-17 | significant | HC |
| PBS - 2xLPS | Ramified | Iba1 | 0.354008712108927 | 0.110409150122965 | Inf | 3.20633490715815 | 0.0161324906962545 | significant | HC |
| PBS - 2xLPS | Rod-like | Iba1 | 0.419587414357389 | 0.112272746584578 | Inf | 3.73721519354925 | 0.00223283600803949 | significant | HC |
| PBS - 2xLPS | Ameboid | P2ry12 | 1.11178818730698 | 0.110982233691799 | Inf | 10.0177131989832 | 1.52900285215326e-22 | significant | HC |
| PBS - 2xLPS | Hypertrophic | P2ry12 | -1.25771325596076 | 0.124932229387118 | Inf | -10.0671641107403 | 9.26053731106909e-23 | significant | HC |
| PBS - 2xLPS | Ramified | P2ry12 | -0.198771564423353 | 0.118567245605471 | Inf | -1.67644582960761 | 1 | ns | HC |
| PBS - 2xLPS | Rod-like | P2ry12 | 0.22110414672808 | 0.119971661844477 | Inf | 1.84296977576842 | 0.784001081872961 | ns | HC |
| PBS - 2xLPS | Ameboid | Cx3cr1 | 0.541913702849729 | 0.0872975315681917 | Inf | 6.2076635285663 | 6.45337650436003e-09 | significant | STR |
| PBS - 2xLPS | Hypertrophic | Cx3cr1 | -0.915584387868001 | 0.0998507739380866 | Inf | -9.16952720302116 | 5.70117816953855e-19 | significant | STR |
| PBS - 2xLPS | Ramified | Cx3cr1 | -0.173660237239669 | 0.0633520254162726 | Inf | -2.74119471474802 | 0.073459461879648 | ns | STR |
| PBS - 2xLPS | Rod-like | Cx3cr1 | 0.379152974781167 | 0.0728534254751204 | Inf | 5.2043259779274 | 2.33642372908557e-06 | significant | STR |
| PBS - 2xLPS | Ameboid | Iba1 | 0.520827436327169 | 0.083615105512004 | Inf | 6.22886777619861 | 5.63781704161309e-09 | significant | STR |
| PBS - 2xLPS | Hypertrophic | Iba1 | -0.691882136217206 | 0.0810864689810153 | Inf | -8.53264601248323 | 1.71645416578275e-16 | significant | STR |
| PBS - 2xLPS | Ramified | Iba1 | -0.154439301948555 | 0.0655371171845701 | Inf | -2.35651656012901 | 0.221366941289771 | ns | STR |
| PBS - 2xLPS | Rod-like | Iba1 | 0.372930463502995 | 0.072771302206184 | Inf | 5.12469135767786 | 3.57630641178836e-06 | significant | STR |
| PBS - 2xLPS | Ameboid | P2ry12 | 1.10551815647492 | 0.0708104615532885 | Inf | 15.6123563132399 | 7.19762733899298e-54 | significant | STR |
| PBS - 2xLPS | Hypertrophic | P2ry12 | -0.996366177798989 | 0.0948015457455048 | Inf | -10.5100203795594 | 9.32121132107904e-25 | significant | STR |
| PBS - 2xLPS | Ramified | P2ry12 | -0.620381924503334 | 0.0686221982642784 | Inf | -9.04054285923796 | 1.87074808471697e-18 | significant | STR |
| PBS - 2xLPS | Rod-like | P2ry12 | 0.128757288171868 | 0.0784243229624148 | Inf | 1.64180299310426 | 1 | ns | STR |
