# Supplementary material for: Development of a High-Throughput Pipeline to Characterize Microglia Morphological States at a Single-Cell Resolution
Source: eNeuro. 2024 Jul 26;11(7):ENEURO.0014-24.2024. doi: 10.1523/ENEURO.0014-24.2024 (PMC11289588; doi:10.1523/ENEURO.0014-24.2024)
Supplement: Table 4-3 — Analysis of Deviance (Type II Wald chisquare tests) on models fit for each brain region separately for area measure: Value ∼ Treatment*Antibody + (1|MouseID). Significance denoted at Pr(>Chisq) < 0.05, related to Fig. 4C. Download Table 4-3, DOC file. [file eneuro-11-ENEURO.0014-24.2024-s009.doc]

| **Variable** | **Chisq** | **Df** | **Pr(>Chisq)** | **measure** | **Significant** | **BrainRegion** |
| --- | --- | --- | --- | --- | --- | --- |
| Treatment | 0.0728989347212746 | 1 | 0.787161771287461 | Area | ns | FC |
| Antibody | 164.595611883593 | 2 | 1.81349769005245e-36 | Area | significant | FC |
| Treatment:Antibody | 46.4892817293655 | 2 | 8.03490485862884e-11 | Area | significant | FC |
| Treatment | 0.537819506001404 | 1 | 0.463337801301243 | Area | ns | HC |
| Antibody | 854.624137597437 | 2 | 2.63467147861659e-186 | Area | significant | HC |
| Treatment:Antibody | 189.850770736727 | 2 | 5.94879416328605e-42 | Area | significant | HC |
| Treatment | 4.22677436863528 | 1 | 0.0397909941486395 | Area | significant | STR |
| Antibody | 1376.90615698023 | 2 | 1.02006292350603e-299 | Area | significant | STR |
| Treatment:Antibody | 255.691032377862 | 2 | 3.00191079846891e-56 | Area | significant | STR |
