# Supplementary material for: Development of a High-Throughput Pipeline to Characterize Microglia Morphological States at a Single-Cell Resolution
Source: eNeuro. 2024 Jul 26;11(7):ENEURO.0014-24.2024. doi: 10.1523/ENEURO.0014-24.2024 (PMC11289588; doi:10.1523/ENEURO.0014-24.2024)
Supplement: Table 4-4 — Tests between treatments across antibodies (∼Treatment|Antibody), Bonferroni-corrected for each brain region. Significance denoted at adjusted p-values (or q-values) < 0.05, related to Fig. 4C. Download Table 4-4, DOC file. [file eneuro-11-ENEURO.0014-24.2024-s010.doc]

| **contrast** | **Antibody** | **estimate** | **SE** | **df** | **t.ratio** | **p.adjust** | **measure** | **Significant** | **Brain Region** |
| --- | --- | --- | --- | --- | --- | --- | --- | --- | --- |
| PBS - 2xLPS | Cx3cr1 | 401.099989480351 | 425.465523599753 | 5.99578807919021 | 0.942732059901703 | 1 | Area | ns | FC |
| PBS - 2xLPS | Iba1 | 664.495874369117 | 425.465523599753 | 5.99578807919021 | 1.56180897748657 | 0.508177738725191 | Area | ns | FC |
| PBS - 2xLPS | P2ry12 | -1374.81242222092 | 425.465523599753 | 5.99578807919021 | -3.23131334024198 | 0.0536959269547625 | Area | ns | FC |
| PBS - 2xLPS | Cx3cr1 | 207.179063188387 | 317.334307145939 | 3.61069930981128 | 0.652873195626805 | 1 | Area | ns | HC |
| PBS - 2xLPS | Iba1 | 641.994040397404 | 317.334307145939 | 3.61069930981128 | 2.02308425512328 | 0.36194869238607 | Area | ns | HC |
| PBS - 2xLPS | P2ry12 | -1514.90548387325 | 317.334307145939 | 3.61069930981128 | -4.7738471692459 | 0.0339563133144813 | Area | significant | HC |
| PBS - 2xLPS | Cx3cr1 | -122.033376895666 | 397.093955886329 | 4.37292064684652 | -0.307316127799736 | 1 | Area | ns | STR |
| PBS - 2xLPS | Iba1 | -140.980699311271 | 397.093955886329 | 4.37292064684652 | -0.355031088288908 | 1 | Area | ns | STR |
| PBS - 2xLPS | P2ry12 | -2131.54573868532 | 397.093955886329 | 4.37292064684652 | -5.36786245947167 | 0.0134987015353636 | Area | significant | STR |
